# Genetics of the phenotypic evolution in sheep: a molecular look at diversity-driving genes

**DOI:** 10.1186/s12711-022-00753-3

**Published:** 2022-09-09

**Authors:** Peter Kalds, Shiwei Zhou, Yawei Gao, Bei Cai, Shuhong Huang, Yulin Chen, Xiaolong Wang

**Affiliations:** 1 Key Laboratory of Animal Genetics, Breeding and Reproduction of Shaanxi Province, College of Animal Science and Technology, Northwest A&F University, Yangling, 712100 China; 2 Department of Animal and Poultry Production, Faculty of Environmental Agricultural Sciences, Arish University, El-Arish, 45511 Egypt; 3 College of Veterinary Medicine, Northwest A&F University, Yangling, 712100 China; 4 International Joint Agriculture Research Center for Animal Bio-Breeding, Ministry of Agriculture and Rural Affairs, Yangling, 712100 China

## Abstract

**Background:**

After domestication, the evolution of phenotypically-varied sheep breeds has generated rich biodiversity. This wide phenotypic variation arises as a result of hidden genomic changes that range from a single nucleotide to several thousands of nucleotides. Thus, it is of interest and significance to reveal and understand the genomic changes underlying the phenotypic variation of sheep breeds in order to drive selection towards economically important traits.

**Review:**

Various traits contribute to the emergence of variation in sheep phenotypic characteristics, including coat color, horns, tail, wool, ears, udder, vertebrae, among others. The genes that determine most of these phenotypic traits have been investigated, which has generated knowledge regarding the genetic determinism of several agriculturally-relevant traits in sheep. In this review, we discuss the genomic knowledge that has emerged in the past few decades regarding the phenotypic traits in sheep, and our ultimate aim is to encourage its practical application in sheep breeding. In addition, in order to expand the current understanding of the sheep genome, we shed light on research gaps that require further investigation.

**Conclusions:**

Although significant research efforts have been conducted in the past few decades, several aspects of the sheep genome remain unexplored. For the full utilization of the current knowledge of the sheep genome, a wide practical application is still required in order to boost sheep productive performance and contribute to the generation of improved sheep breeds. The accumulated knowledge on the sheep genome will help advance and strengthen sheep breeding programs to face future challenges in the sector, such as climate change, global human population growth, and the increasing demand for products of animal origin.

**Supplementary Information:**

The online version contains supplementary material available at 10.1186/s12711-022-00753-3.

## Background

**Fig. 1:**
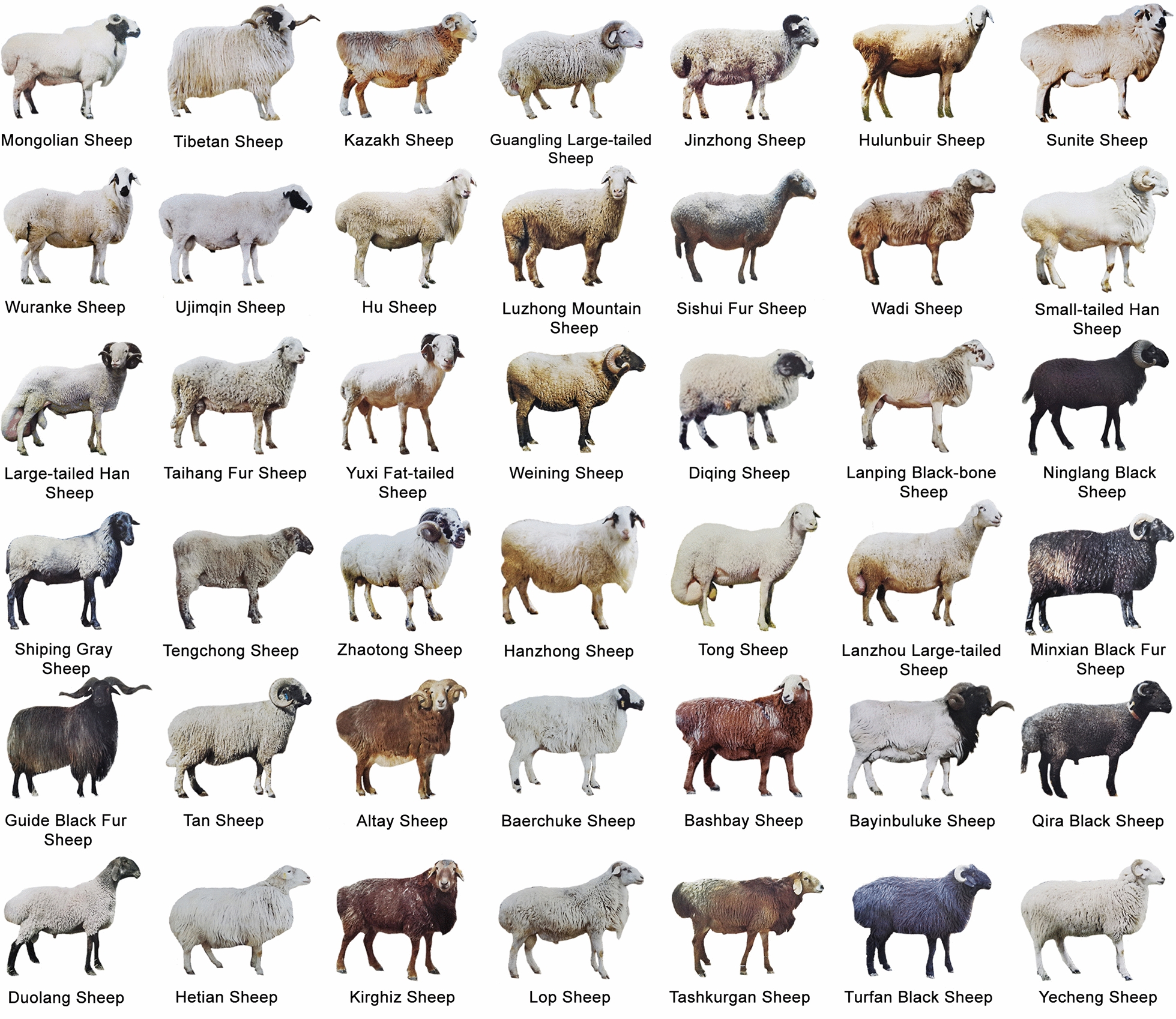
Phenotype variation in sheep breeds. A representative example of phenotypic diversity in the Chinese sheep population (approximately 42 different indigenous breeds). Moreover, several imported sheep breeds (approximately 8) can be found in China, and over 21 improved and crossed breeds were generated by breeding local and imported breeds to yield multipurpose breeds for improved meat and wool production, as well as other productive traits. Images used herein were adapted with permission from *Animal Genetic Resources in China: Sheep and Goats* [217]

One of the most intriguing questions in the biological sciences is how a genome can result in wonderfully varied phenotypes? Throughout and following sheep domestication, various genotypes have been generated. Certain genomic patterns have been selected naturally or artificially to give rise to various phenotypes [1–3], which have culminated in the generation of distinct currently known sheep breeds (Fig. 1). Among the main phenotypic traits that vary widely in sheep, are included tails, horns, and coat colors. For instance, large-fat tails in sheep appeared as an adaptive response to harsh environmental conditions and food scarcity [4]. However, the thin-tail phenotype has currently become more desirable, considering that the fat-tail phenotype carries several negative implications, which likely influence animal locomotion, mating, food conversion, breeding costs, among others. Sheep horns are commonly used for protection against predators and as sexual weaponry. In modern breeding practices, the hornless (or polled) phenotype has become increasingly desirable as a way to avoid the need for dehorning [5], which is usually performed to protect animals and producers from accidental injuries. In addition, coat color serves in the adaptation to environmental conditions and avoiding predators. Currently, selection for specific sheep coat color patterns is performed based on the production system, e.g., white color is desirable in several wool-producing sheep breeds [6]. Combined with other phenotypic traits, coat color is considered a determinant of sheep breeds. Several phenotypic traits are significantly associated with production and animal welfare parameters. Hence, understanding the genetic background of these phenotypic traits is of great importance for a rapid and direct selection and improvement of sheep breeds. The sequencing of the sheep genome and the elucidation of its content and variations have offered an unprecedented opportunity for a better understanding of the underlying genetic traits controlling sheep phenotype [7–10].

Recent advances in sheep genomics have helped in identifying genes that are implicated in determining major phenotypic traits in sheep. For instance, it has been reported that the *PDGFD*, *BMP2*, *HOXB13*, and *TBXT* genes regulate sheep tail phenotype [11–14], *RXFP2* and *HOXD1* regulate horn and multi-horn traits [5, 15], whereas *ASIP*, *MC1R*, and *TYRP1* regulate coat color patterns [16–18]. However, the causative genomic variations within or around some of these genes are currently unknown. Given the importance of the genes controlling sheep phenotypic traits that are linked with adaption, production, and welfare traits, in this review, we collectively discuss the genomic history of these traits and describe genes and variants that are currently known for these traits and highlight traits which need further investigation to elucidate their genetic determinism. We discuss potential genes and their variants that control main phenotypic traits in sheep, including tail, horns, coat color, bone color, fat color, wool length, fleece type, ear size, number of nipples, number of thoracic and lumbar vertebrae, body height, and stature. We anticipate that exploring genes that control significant phenotypic traits in sheep will foment selective breeding programs and genetic-based strategies for the rapid improvement of productivity and welfare of sheep populations.

## Main text

### The long-standing conundrum of sheep tails: a genomic perspective

**Fig. 2:**
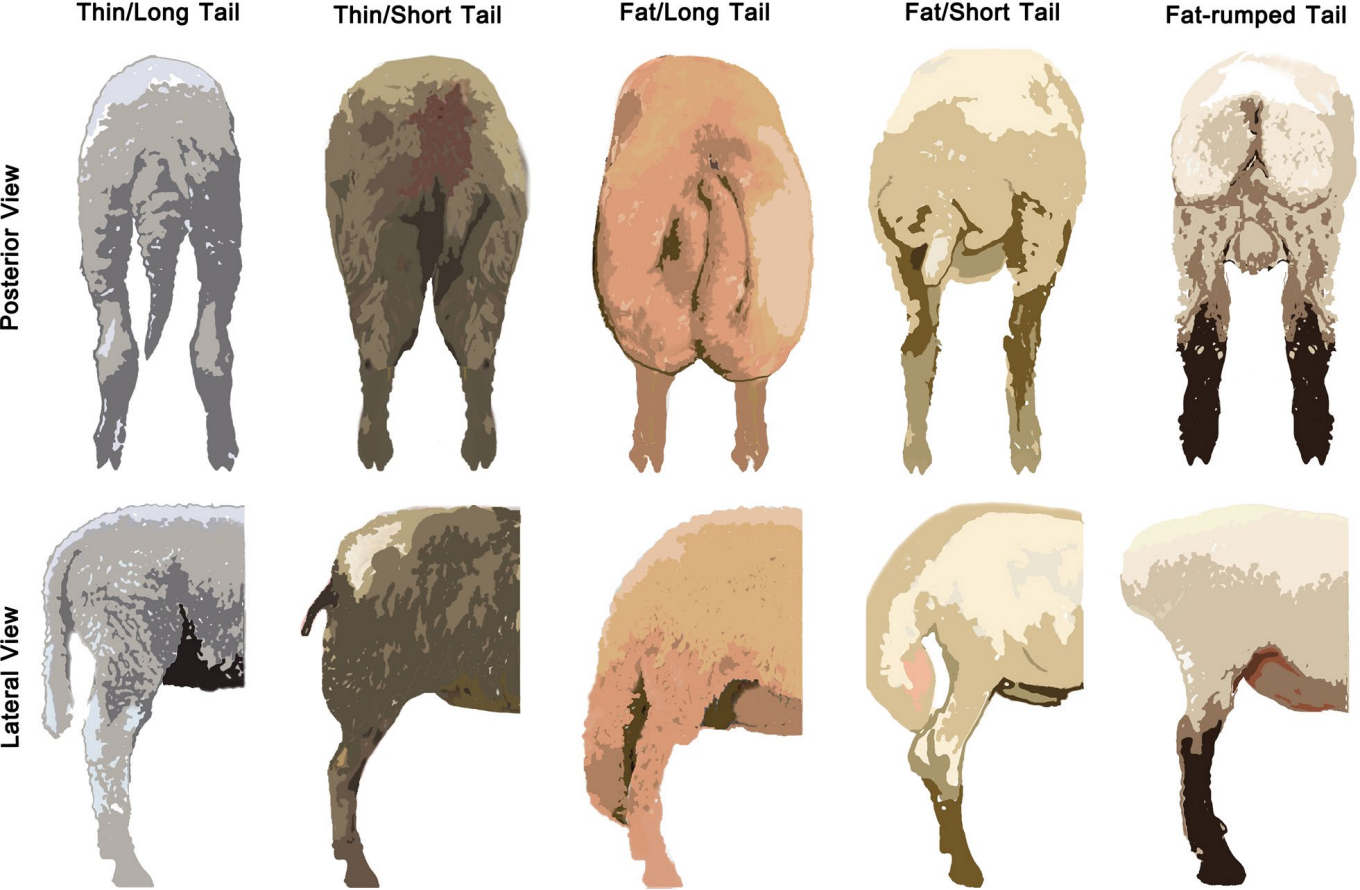
Phenotype variation in sheep tails. Representative examples of common sheep tail patterns. The figure was conceived by Peter Kalds and designed by Guangyuan Cao

Sheep tail phenotypic patterns vary among different sheep breeds in terms of length, directionality, and fat accumulation level [19] (Fig. 2). The genetic basis of sheep tail phenotypic variations has been elucidated based on genomic studies. Moradi et al. [4] first applied a genomic scan approach to identify genomic loci that underlie the phenotypic differences between fat- and thin-tailed Iranian sheep breeds, and revealed seven genomic regions that overlap with quantitative trait loci (QTL) shown to affect fat and carcass yield. In addition, three genomic regions located on *Ovis aries* chromosome 5 (OAR5), OAR7, and OARX were suggested to be associated with the sheep tail phenotype [4]. A subsequent genome-wide analysis by Wei et al. [11] using ten representatives of Chinese sheep breeds revealed the association of *PPP1CC* and *PDGFD* with significant differences between fat- and thin-tailed animals. Specifically, *PDGFD* was positively selected in fat-tailed sheep breeds. In another study, *BMP2* and *VRTN* were identified as the genes that are most probably involved in the determination of sheep tail phenotype [12].

Subsequently, Yuan et al. [20] proposed that the *HOXA11*, *BMP2*, *PPP1CC*, *SP3*, *SP9*, *WDR92*, *PROKR1*, and *ETAA1* genes potentially play an important role in determining the fat-tailed phenotype. An integrated analysis of genome-wide association study (GWAS) and X-chromosome-wide association analysis (XWAS) in small-tailed and large-tailed Han sheep revealed a set of genes (namely, *CREB1*, *STEAP4*, *CTBP1*, and *NRIP1*) that could be potentially associated with lipid metabolism or adipocyte development [21]. A candidate genomic region, i.e., OARX 88–89 Mb, was also identified, which was shown to contain significant single nucleotide polymorphisms (SNPs) that could be associated with tail phenotype [21]. In addition, by analyzing genotypic data of 11 Ethiopian indigenous sheep breeds and two populations of thin-tailed sheep from Sudan, a set of potential genes associated with sheep tail phenotype was proposed [22]. This includes putative candidate regions that spanned genes affecting growth traits and fat deposition (*NPR2*, *HINT2*, *SPAG8*, and *INSR*), tail formation (*ALX4*, *HOXB13*, and *BMP4*), embryonic development of tendons, bones, and cartilages (*EYA2* and *SULF2*), lipogenesis and intracellular long-chain fatty acids transport (*FABP3*) [22]. In another study, genomic data of three Iranian sheep breeds (i.e., Baluchi, Lori-Bakhtiari, and Zel) were also analyzed, which revealed that the *RPS6KA3*, *HOXB13*, *HOXB9*, *ACAN*, and *CHRDL1* genes as potentially influencing the skeletal system and tail traits when comparing fat-tailed Baluchi sheep and thin-tailed Zel sheep; conversely, *NPR2* and *EN1* were suggested as potentially influencing energy metabolism, skeletal system, and tail traits when comparing fat-tailed Lori-Bakhtiari sheep and thin-tailed Zel sheep [23].

Pan et al. [24] analyzed the whole-genome sequences of 89 sheep and identified two significant signals on OAR13 and OAR15. The sweep signal on OAR13 was consistent with previous studies [11, 12]. In particular, Moioli et al. [12] suggested that the *BMP2* gene could be potentially causative for the sheep fat tail formation, whereas Wei et al. [11] suggested the *PPP1CC* gene, which is a paralogue of the original *PPP1CC* gene on OAR13 (NCBI gene identifier: *LOC101117953*). The other signal on OAR15 contained the *PDGFD* gene, which was previously highlighted by Wei et al. [11]. The newly characterized *LOC101117953* has been suggested to originate from a retro-copy (*r-PPP1CC*) of the *PPP1CC* gene via clear intron losses [24]. Since *r-PPP1CC* is a promoter-less non-functional pseudo-gene, it was assumed to be less likely the causative gene. However, the locus can potentially serve as a regulatory element, which changes the expressional patterns of the distant *BMP2* gene [24]. Mastrangelo et al. [25] compared the genomic data of two fat- and thin-tailed sheep breeds, and again revealed previously reported genes, i.e., *VRTN* and *BMP2*, with a potential role in determining variations in the number of vertebrae and tail formation, respectively, and *PDGFRA* and *PDGFD*, which seem to be involved in preadipocyte differentiation. Moreover, Mastrangelo et al. [26] also reported a set of genes, namely, *ANAPC1*, *MSRB3*, *CXXC5*, *PSD2*, *SLIT2*, *EPHA5*, *CHP1*, *OIP5*, *PCDH9*, *CDS2*, *BMP2*, and *OAS2*, which could be associated with fat deposition in sheep breeds from Africa and Eurasia.

Recently, a number of large-scale genomic studies have been performed to elucidate the phenotypic differences in sheep tail formation. Li et al. [27] investigated the genetic background of the configuration of sheep tails by performing separate pairwise-population selection tests and analyzing genotype patterns located in the promoter region; in this study, the *PDGFD* gene was consistently selected by multiple comparisons and its expression varied between fat- and thin-tailed sheep breeds. Dong et al. [28] highlighted a 6.8-kb region within the first intron of the *PDGFD* gene that contained the most differentiated SNPs between fat- and thin-tailed sheep breeds and that likely harbors regulatory mutation(s) determining the fat-tail configuration. Furthermore, it has been demonstrated that expression of *PDGFD* was high on days 60 and 70 of embryonic development in the tail adipose tissue, which suggests a potential role of *PDGFD* in the initiation and/or commitment of preadipocytes; in contrast, expression of *PDGFD* decreased between days 80 and 90 of embryonic development when fat started to accumulate in the tail, thus suggesting a negative association with fat maturation [28].

Baazaoui et al. [29] employed a combination of runs of homozygosity (ROH) hotpots analysis and an *F*_ST_-outlier approach to identify genomic regions under selection in Tunisian sheep breeds. An outstanding selection signal harbored several relevant genes linked with lipid metabolic processes (i.e., *CDS2*, *PROKR1*, and *BMP2*). The study also emphasized the potential involvement of *BMP2* in the sheep tail formation [29]. Genes linked with the skeletal tail phenotype, including *HOXB13*, *NR5A1*, *NR6A1*, *KDM6B*, and *NR3C1*, have also been proposed to play a major role in the adaptation of African sheep [30]. In addition, allele-specific expression (ASE) in thin-tailed Texel sheep and fat-rumped Kazakh sheep was investigated [31]. In this study, 2405 ASE genes were described from adipose tissue; among these, *PDGFD* and *LYPLA1* were highlighted as possibly linked with lipid-related biological processes. In addition, seven ASE SNPs were found in the *PDGFD* gene, and a distinct genotype pattern located in the upstream region of *PDGFD* was detected by selective sweep analysis between mouflon, fat-tailed, and thin-tailed sheep. Moreover, potential exon splicing events within the *PDGFD* gene in thin-tailed sheep have been described [31]. More recently, Li et al. [13] emphasized the significance of both *PDGFD* and *BMP2* signals in sheep tail formation, and described a new variant based on a 169-bp insertion close to the 5′-UTR region of *HOXB13*, which is likely to be associated with the long tail phenotype. This variant was further confirmed as a 167-bp segment with a linked missense mutation (*c.23C*>*G*) in Merinolandschaf sheep [32]. It is likely that multiple genes may contribute to the configuration of sheep tails since tail phenotype and related fat deposition levels vary greatly between different sheep breeds from different geographical regions.

**Fig. 3:**
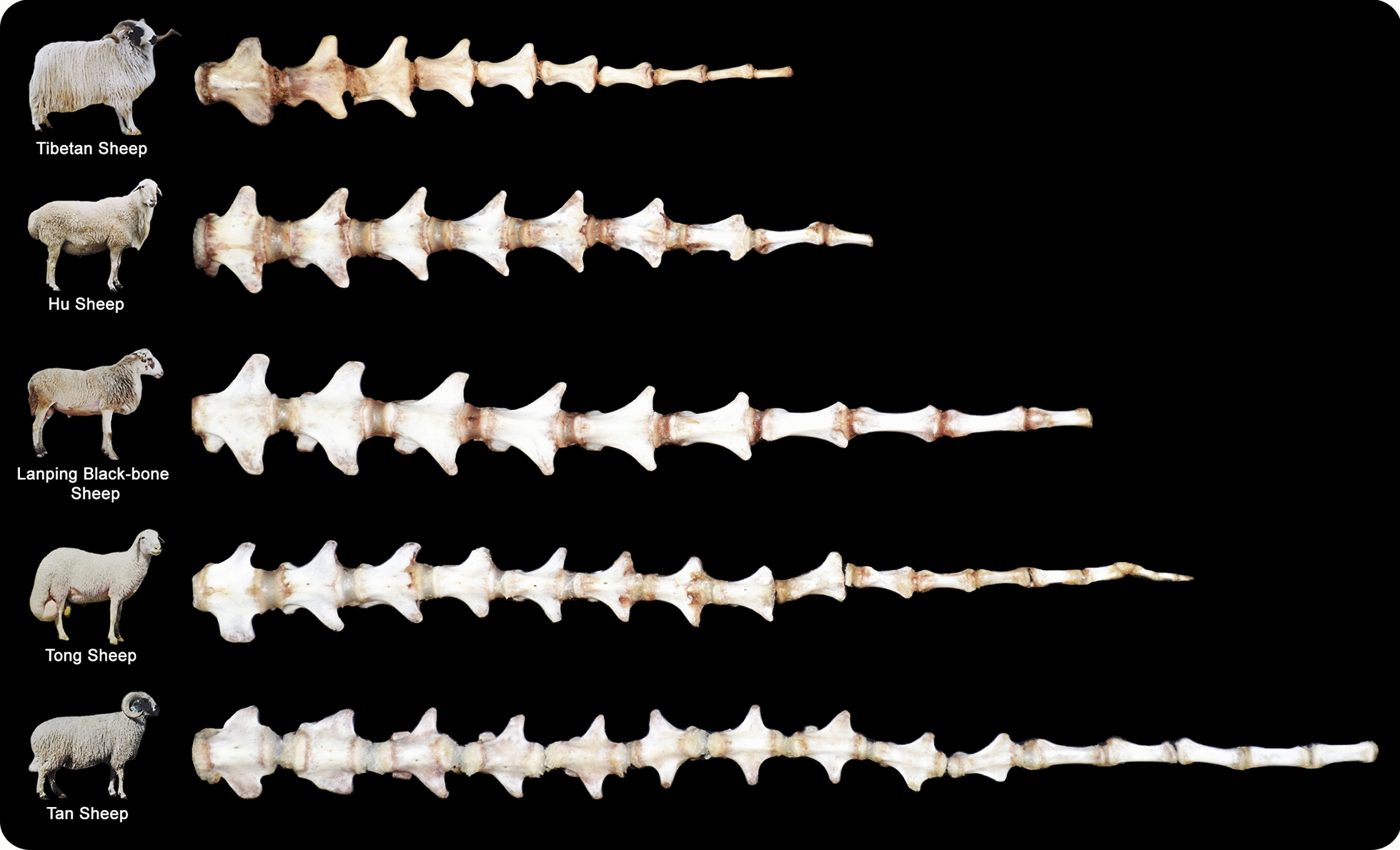
Phenotype variation in sheep caudal vertebrae. Representative examples of caudal vertebrae among Chinese sheep breeds. From up to bottom, Tibetan (thin/short tail), Hu (fat/short tail), Lanping Black-bone (thin/moderate length tail), Tong (fat/long tail), and Tan sheep (fat/long tail)

Although the level of fat deposition mainly determines sheep tail phenotype, the length and number of caudal vertebrae have also been considered as important factors that influence tail morphology (Fig. 3). Zhi et al. [14] analyzed genomic differences between two lines of Chinese Hulunbuir sheep of short tail and normal tail, which revealed the *TBXT* gene (also known as *T*; *Brachyury*) as a candidate for determining the number of caudal vertebrae in sheep. Subsequently, Han et al. [33] confirmed the previously reported missense mutation (*c.334G*>*T*) with a completely linked synonymous variant (*c.333G*>*C*) to be associated with the characteristic short caudal vertebrae in fat-rumped sheep breeds. Interestingly, the functional role of the *TBXT* gene in forming the sheep tail length was validated using the CRISPR/Cas9 system [34]. Collectively, the abovementioned findings provide significant information obtained in research studies from the past few decades to understand the genetic causes of phenotypic variations in the configuration of sheep tails. These genomic studies identified sets of genes that could potentially be associated with phenotypic variations in sheep tails (see Additional file 1: Fig. S1a), and narrowed down the candidate genes and genomic regions involved in sheep tail formation (e.g., *BMP2*, *LOC101117953*, *PDGFD*, *HOXB13*, and *TBXT*). Further genomic investigations of sheep tails are shown in Additional file 2: Table S1. Moreover, sheep tail formation has been investigated using transcriptomic [mRNAs (see Additional file 2: Table S2 and Additional file 1: Fig. S1b), miRNAs (see Additional file 2: Table S3), and lncRNAs (see Additional file 2: Table S4)] and proteomic (see Additional file 2: Table S5) approaches, as well as direct gene expression and potential SNP associations (see Additional file 2: Table S6). These studies further identified potential genes, regulatory factors, and molecular pathways that might contribute to the formation and phenotype variation of sheep tails.

### Development of sheep horns: the remainders of their wild past

#### The *RXFP2* gene and its role in sheep horn development

**Fig. 4:**
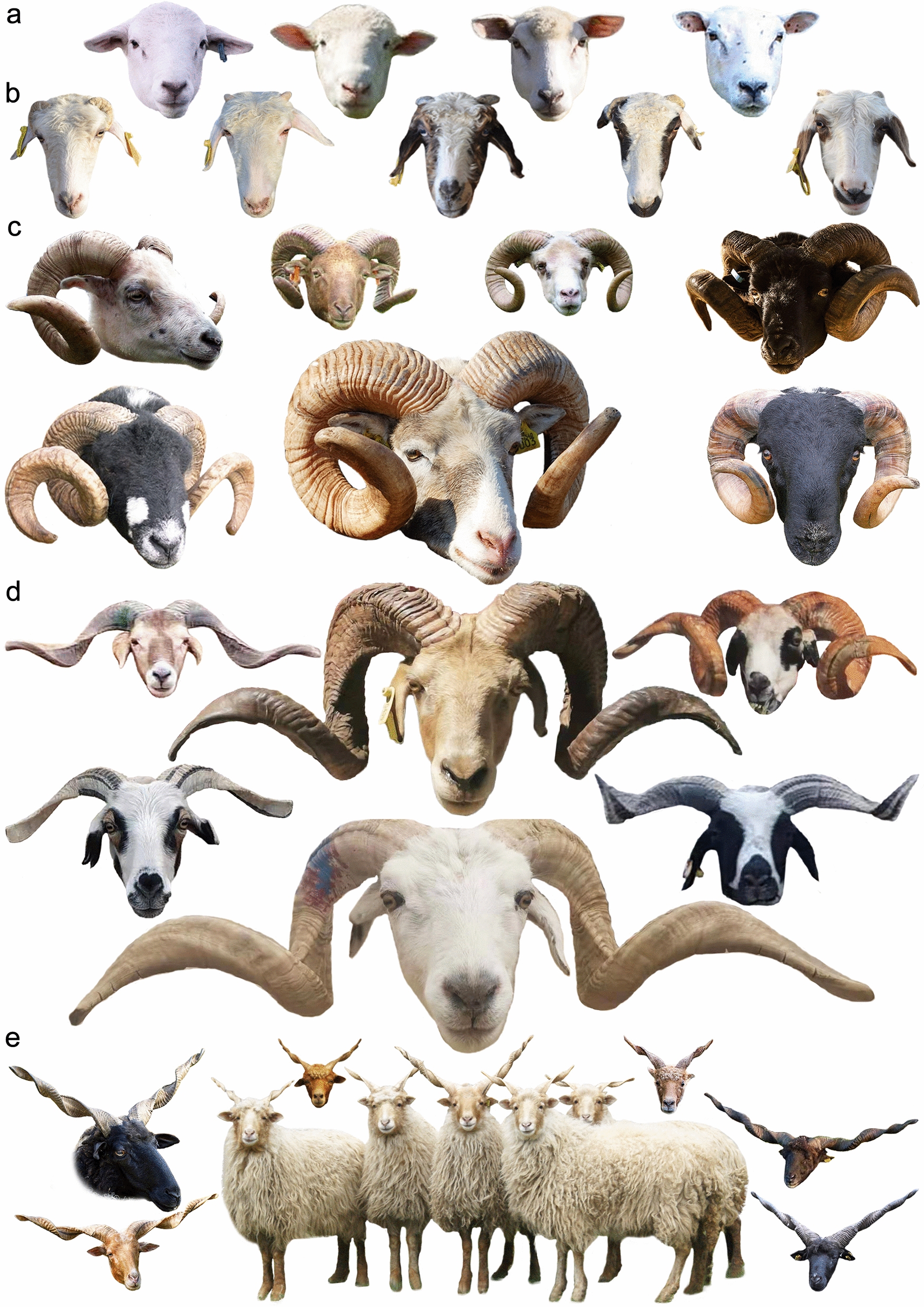
Phenotype variation in sheep horns. **a** Polled phenotype, **b** scurred phenotype, **c** horned phenotype (curled tightly close to the face), **d** horned phenotype (spiral and horizontal extension; found in Chinese Tibetan sheep), and **e** horned phenotype (multi-spiral and up-forwarded; found in Hungarian Racka sheep). Photo credits and courtesies are shown in Additional file 2: Table S14

**Fig. 5:**
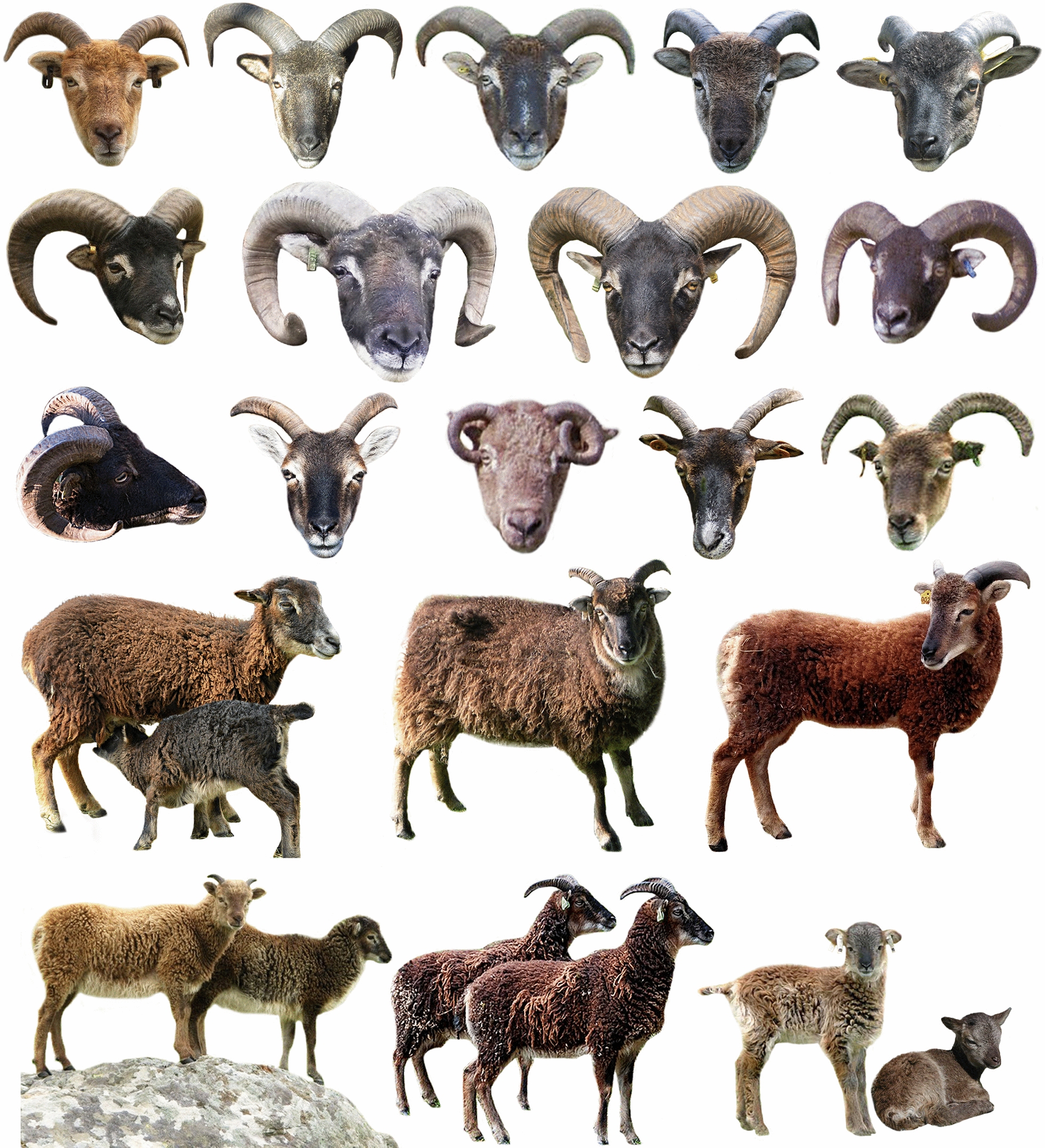
Phenotype of Soay sheep at different developmental stages. Soay sheep are a well-studied sheep population which have significantly contributed to the understanding of the genomic background of various important phenotypic traits. Photo credits are shown in Additional file 2: Table S14

Horns (cranial appendages or headgear) [35] are typically used by male animals in mating conflicts for dominance [18]. Sheep can be horned, polled (hornless), or scurred (i.e., vestigial and deformed horns) (Fig. 4). Hornedness and polledness phenotypes have varying patterns among different sheep breeds. In certain cases, horns are found in both sexes, but their size is larger in males; males can be horned but females polled; and both sexes can be polled [36]. In other cases, horned and polled male and female representatives can be found within the same breed. Early attempts to understand the genetics of sheep horn development date from over a century ago [37–41]. Since then, several efforts have been made to understand the mode of inheritance of this unique phenotypic trait in sheep [42–48]. With the emergence of genomic-based approaches, the underlying genetic basis of this phenotypic trait in sheep has been progressively revealed. In 1996, Montgomery et al. [49] refuted the existence of a genetic locus determining horn traits in sheep on OAR1 as seen in cattle. However, a horn-determining locus was mapped on OAR10 in sheep that originate from Merino and Romney crosses. Subsequently, using a free-living population of Soay sheep (Fig. 5) in St. Kilda, Beraldi et al. [18] also mapped the horn locus on OAR10 at a location similar to that previously described in modern domestic sheep. In addition, Pickering et al. [50] localized the horn locus within a 14-Mb region using six genetic markers. Subsequently, by using fine-mapping with additional genetic markers, the horn locus was localized within a 50-kb region. This study also reported a large (approximately 3 kb) retrotransposed insertion in the non-coding 3′-UTR region of a then unnamed candidate gene, which was found exclusively in polled individuals [50].

Johnston et al. [51] further narrowed the horn locus down to a ~ 7.4-cM interval on OAR10 in a Soay sheep population. Subsequently, Johnston et al. [5] performed a GWAS using ~ 36,000 SNPs and determined that the *RXFP2* gene was the main candidate for determining horn phenotype in sheep. Consistently, when comparing the genomes of global sheep breeds using the OvineSNP50K BeadChip, the strongest SNP under selection was determined to be located immediately adjacent to *RXFP2*, which suggested that *RXFP2* can be potentially targeted by breeding for horn removal, being likely one of the oldest morphological changes occurring during sheep domestication [8]. Johnston et al. [52] later described two *RXFP2* alleles in Soay sheep, namely, *Ho*^+^ and *Ho*^*P*^, with the *Ho*^+^ allele conferring larger, normal horns and the *Ho*^*P*^ allele conferring smaller horns. *Ho*^+^*Ho*^+^, *Ho*^+^*Ho*^*P*^, and *Ho*^*P*^*Ho*^*P*^ males were horned, and approximately half of the *Ho*^*P*^*Ho*^*P*^ males were scurred. In contrast, the *Ho*^+^*Ho*^+^ genotype led to horned, *Ho*^+^*Ho*^*P*^ to scurred, and *Ho*^*P*^*Ho*^*P*^ to polled females. In addition, it has been proposed that the *Ho*^+^ allele that confers larger horns is associated with higher reproductive success, whereas the smaller horn *Ho*^*P*^ allele is associated with increased survival. Collectively, these pieces of evidence suggest a trade-off between reproduction and survival for this allele in male Soay sheep. Several subsequent genomic studies highlighted the role of *RXFP2* in determining horn phenotype when comparing different regional sheep populations. Pan et al. [53] described *RXFP2* as a consistently strong marker of positive selection based on a GWAS in two semi-feral sheep populations from the Qinghai-Tibetan Plateau. In addition, the expression of *RXFP2* in sheep has been studied in 13 tissues, and expression was exclusively in horn-related tissues [53]. Moreover, an introgression of genomic regions into the *RXFP2* locus in Tibetan sheep from Argali sheep has been suggested, in which two missense variants (*c.1999C*>*T* and *c.1957C*>*T*) were also detected [54]. Recently, the same region was suggested to be introgressed from Iranian mouflon into Tibetan sheep [55]. Nevertheless, in this region, three major highly divergent haplogroups were found in haplotype patterns across sheep breeds with different horn phenotypes [55].

Wiedemar et al. [56] described a 1833-bp genomic insertion (namely, an *EEF1A1-like* insertion) in the flanking 3′-region of the sheep *RXFP2* gene that is potentially associated with polledness. In this study, this region was genotyped in seven Swiss sheep breeds. It was shown that horned individuals were homozygous for the absence of the insertion, whereas polled individuals were homozygous for its presence. It has been speculated that when the *EEF1A1-like* insertion is present, *EEF1A1* transcripts bind to the 3′-UTR of the *RXFP2* mRNA, leading to post-transcriptional downregulation [56, 57]. In Bündner Oberländer sheep with both polled and horned phenotypes, horned individuals were homozygous for the absence of the insertion; however, two polled individuals were found to be heterozygous for the insertion. Later, Lühken et al. [58] reported that the actual length of the *EEF1A1-like* insertion was 1780 bp. By genotyping this insertion in a large number of sheep breeds and individuals with different horn statuses, it was concluded that this insertion cannot be considered as the exclusive determinant of polledness in sheep, and thus could not be considered as a universal marker for determining the genetic causality of horns in sheep. The insertion was shown to segregate with horn status (such as polled or horned) only to a certain extent and within certain sheep breeds. In other breeds with variable horn statuses in both sexes or with a sex-dependent horn status, the segregation of this potential insertion is weaker. It has been suggested that this insertion is among the potential various causative factors for polledness in sheep [59]. He et al. [60] performed PCR amplification of the DNA of 49 Chinese sheep with three horn statuses (polled, two-horned, and four-horned), and found the *EEF1A1-like* insertion in 68.4% (13/19) of the polled individuals and 43.3% (13/30) of the horned individuals. These findings suggest a yet unverified association between the *EEF1A1-like* insertion and the polledness phenotype in sheep.

Large-scale investigations have also been performed to elucidate the origins of mammalian horns [61, 62]. Wang et al. [61] identified 624 horn-related genes that are highly co-expressed in bone, skin, testis, and brain tissues. In addition, 201 genes were commonly found in both horn and antler tissues, and these genes were enriched in bone and skin development, and neurogenesis pathways. This study provided important insights into the origin of horns and further proposed a number of genes potentially associated with horn development regulation. Among these genes, *SOX10*, *SNAI1*, and *TFAP2A* were highly expressed in fetal sheep horn buds but not in adjacent skin tissues. In addition, this study also suggested a single evolutionary origin for the ruminant headgear, as well as proposed that the horn-stimulating gene *RXFP2* is under convergent pseudogenization in secondarily headgearless lineages [61]. Furthermore, using transcriptomic data, seven highly expressed keratin genes, namely, *KRT1*, *KRT2*, *KRT3*, *KRT5*, *KRT10*, *KRT14*, and *KRT84*, were found to be associated with the formation of keratinous sheath [62]. Type II a-keratin proteins expressed by the above-mentioned genes (except *KRT10* and *KRT14*) have been suggested to play an essential role in the development of the horn keratinous sheath [62]. Further investigations on the sheep horn phenotype are shown in Additional file 2: Table S7. Collectively, these findings highlight the contribution of a gene set to the determination of sheep horn status; in particular, the prominent role of *RXFP2* with the presence/absence of horns in sheep has been suggested. Although a significant association between *RXFP2* and horn development has been indicated, the exact variants and the underlying mechanisms by which *RXFP2* contributes to phenotypic differences in horn status in sheep have not yet been elucidated.

#### Beyond the horned or polled dichotomy: polyceraty in sheep

**Fig. 6:**
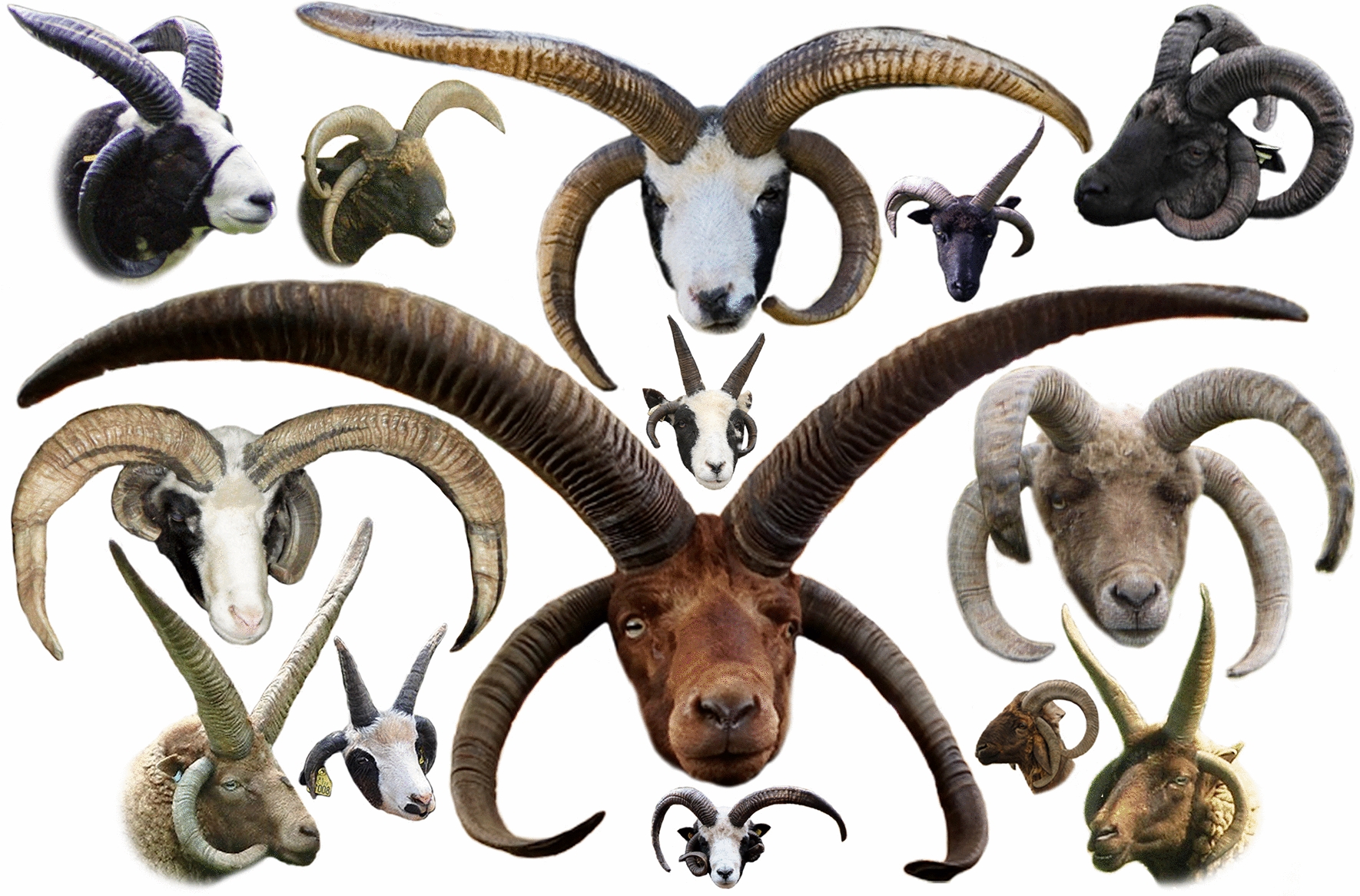
Polyceraty (multi-horned phenotype) in sheep. Representative examples of different sheep breeds. Photo credits are shown in Additional file 2: Table S14

**Fig. 7:**
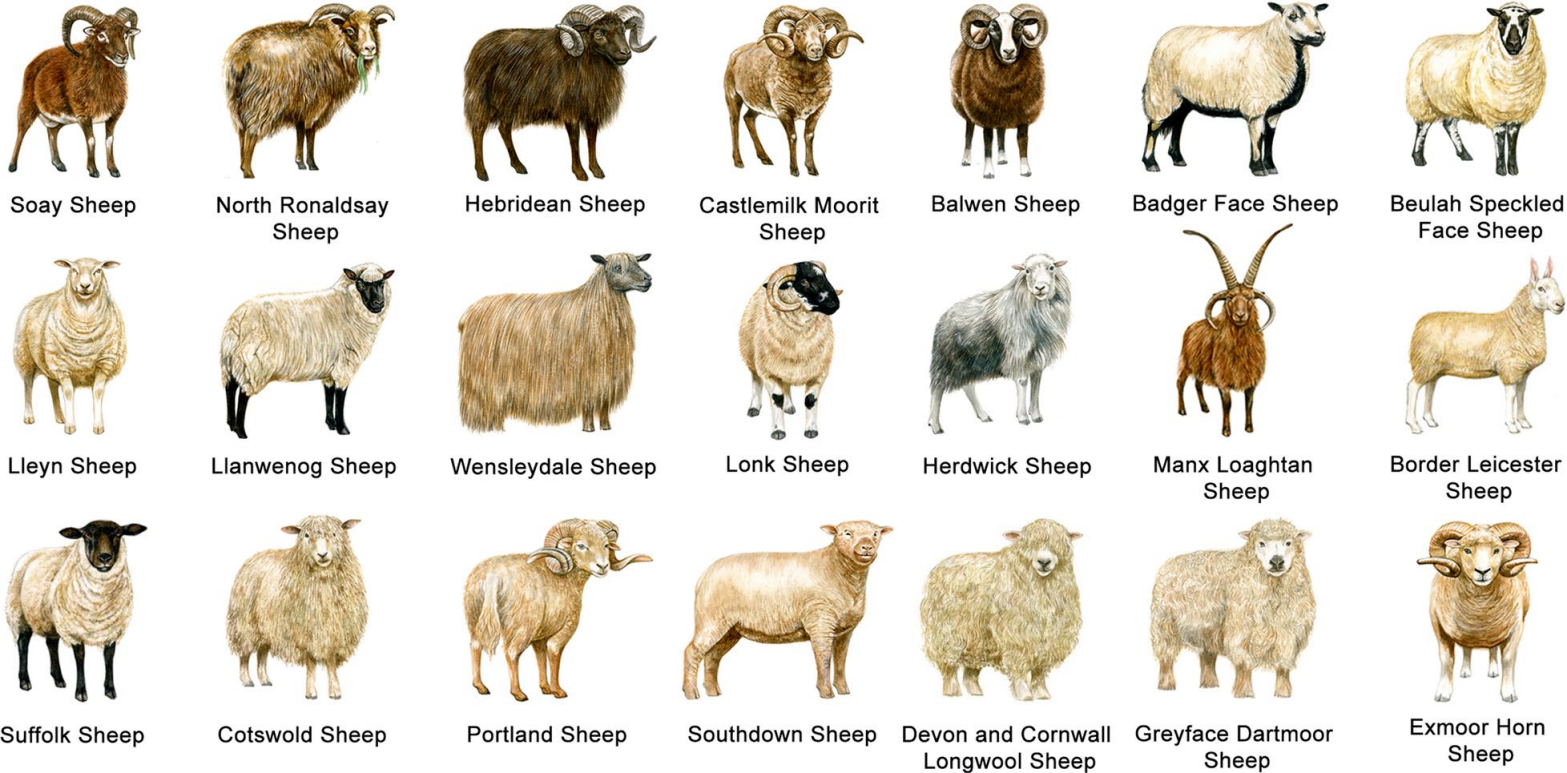
An example of a sheep population from the United Kingdom with different horn patterns. Polledness (hornlessness), hornedness (a pair of horns), and polyceraty (multi-hornedness; more than a pair of horns) are shown. Figure copyright and published with the consent of Fiona Osbaldstone (fi@fionaosbaldstoneillustrator.co.uk)

Polyceraty is related to the condition where the animal carries multiple horns (Fig. 6), and which is considered rare in sheep [63]. Among native sheep populations, only few breeds carry multiple horns (Fig. 7), and polycerate sheep usually carry four or six horns. Ren et al. [64] performed a GWAS comparing 24 two-horned and 22 four-horned Chinese Sishui Fur sheep, which enabled the identification of a potential genomic region (132.0–133.1 Mb) in OAR2 that contained the top ten SNPs associated with the polycerate phenotype. In humans and mice, this genomic region includes the *HOXD* gene cluster, which comprises nine genes, including *HOXD1*, *HOXD3*, *HOXD4*, and *HOXD8*-*HOXD13*, as well as other functional genes, such as *EVX2* and *KIAA171*, which was shown to have a potential association with the formation of limbs and genital buds [64]. Subsequently, Kijas et al. [65] performed a GWAS mapping 125 two- and four-horned individuals from Jacob and Navajo-Churro sheep breeds to investigate the genetic cause of four-hornedness as well as examine whether it was also related with eyelid abnormality at various degrees of severity. This study confirmed that OAR2 harbors a strong marker linked with polyceraty (131.9–132.6 Mb). The *MTX2* gene and the *HOXD* gene cluster (especially *HOXD1*) were the closest to the genetic marker that was most strongly associated with polyceraty. In Jacob and Navajo-Churro sheep breeds, eyelid abnormality was strictly observed in polled individuals and in polycerate individuals, which suggests an absence of eyelid abnormality in two-horned animals and a potential developmental link between eyelid abnormality and polyceraty [65]; thus, this study proposed a pleiotropic effect for the development of the horn trait in sheep. Greyvenstein et al. [66] conducted a GWAS on 43 Damara sheep (19 four-horned, 7 three-horned, 16 two-horned, and one polled), and confirmed that the proposed genomic region (128–135 Mb) spanning nine embryonic development *HOXD* genes is causal for the polyceraty phenotype.

He et al. [60] performed a GWAS on 34 two-horned and 32 four-horned individuals from three Chinese breeds (namely, Altay, Mongolian, and Sishui Fur). The potential genomic region (132.6–132.7 Mb) on OAR2 was similar to that previously identified in polycerate sheep breeds, i.e., Sishui Fur, Jacob, Navajo-Churro, and Damara, which suggests a common genomic cause for the polyceraty phenotype among different sheep breeds. In a subsequent study, the same research group performed another GWAS using 72 polycerate and 24 two-horned Tibetan sheep, to map the genomic regions of typical (four horns) and atypical polycerate (three, five, and six horns) phenotypes [67]. The same candidate genomic region (131.990–133.525 Mb) was identified, thus suggesting that this region affects both typical and atypical polycerate phenotypes. In a more recent study, Allais-Bonnet et al. [15] re-visited the Illumina OvineHD Beadchip genotyping data of 111 cases and 87 control sheep previously reported [65, 66], and identified the *polycerate* locus between the positions 132,717,593 and 133,151,166 bp on OAR2. Subsequently, by comparing whole-genome sequences of 11 polycerate specimens and 1179 control sheep from various populations, a four-nucleotide deletion variant (g.132,832,249_132,832,252del) was revealed at position +4 to +7 bp after the first exon of the *HOXD1* gene. Moreover, this potential variant further showed a perfect association when 236 animals from various populations, containing polycerate specimens, were investigated [15]. This variant was proposed to influence the splicing of *HOXD1* precursor RNAs, thus generating the multi-horn phenotype in sheep.

### Sheep coat color: a major breed determinant

#### The *ASIP* gene

Sheep coat color has been investigated since a long time [68–73] and its variations have sparked the curiosity of a wide spectrum of researchers and specialists to understand its biological and genetic basis [6, 74, 75]. Coat colors in sheep vary widely among and within breeds (see Additional file 1: Fig. S2). In Australian Merino sheep, a uniform white fleece is a desirable trait, and the small proportion of colored-fleece lambs born annually in these flocks leads to significant financial loss for sheep breeders and wool processors. To reveal genes linked to pigmentation in the sheep genome, Parsons et al. [16] used comparative mapping information to investigate the ovine homologous position of the *Agouti* gene. The study showed that the locus responsible for recessive pigmentation in Australian Merino sheep is positioned within OAR13 in a region homologous to murine chromosome 2 and human chromosome 20 regions that contain the *Agouti* gene locus. In a subsequent report [76], the same authors used the available sequencing data of human and mouse homologs to isolate the coding region of the ovine *Agouti* gene, which revealed that the isolated ovine sequence has an authentic agouti homolog at the nucleotide and amino acid levels to other previously reported mammalian species. In addition, by developing a linkage map for loci responsible for certain phenotypic traits in Soay sheep, the ‘coat pattern’ (i.e., intensified coat color in the ‘self’ individuals vs. the contrast in color between the belly and the rest of the body in ‘wild’ individuals) mapped to OAR13 and close to the candidate *Agouti* locus [18].

Smit et al. [77] amplified a 142-bp segment in exon 2 of the *Agouti* gene (*ASIP*) using 361 samples obtained from sheep. Bands of 142 bp were present in all animals and bands of 137 bp (agouti-like sequence) were shown in 173 of the investigated sheep. The sequences of the 142-bp amplicons were identical to the previously published ovine agouti sequence [76] and a 5-bp deletion (D_5_; positions 98–102 from the start codon in the normal agouti transcript) was identified in the 137-bp amplicon. Subsequently, D_5_ was surveyed in ten sheep breeds, and the presence of D_5_ was observed in white-faced sheep breeds but not in black-faced sheep breeds [77]. Royo et al. [77] reanalyzed the potential of D_5_ by sequencing the *ASIP* exon 2 and intron 2 using samples obtained from 188 individuals of the rare Xalda sheep breed, and the deletion (*c.100_104delAGGAA*) was verified in the surveyed individuals. All Xalda individuals carrying the deletion in a homozygous mode were phenotypically black; however, other black-coated Xalda individuals (91%) were not homozygous, which suggests that the homozygous deletion could not be the only determinant of black coat color in sheep. In addition, mRNA levels of *ASIP* was evaluated in 24 (14 black-coated and 10 white-coated) Xalda individuals, which revealed that *ASIP* expression in black-coated individuals was significantly lower than in white-coated individuals, and that D_5_ did not influence *ASIP* expression [78].

The role of *ASIP* in determining coat color pattern was primarily proposed by classical genetics. The dominant white or tan (*A*^*Wt*^) *ASIP* allele is responsible for the yellow/red phenotype (caused by phaeomelanin), whereas the recessive allele non-*agouti* (*A*^*a*^) is responsible for the black/brown phenotype (caused by eumelanin). In addition to other patterns such as badgerface (*A*^*b*^) where the dorsal region is pale and the ventral region is darker. To reveal the genomic basis of these patterns, Norris et al. [79] performed sequence analysis of genomic DNA, bacterial artificial chromosome clones, and RT-PCR products from a group of sheep breeds with different coat colors. Using these molecular tools, the study proposed a model for the evolution of the ovine *ASIP* locus. This includes (i) a 190-kb tandem duplication encompassing the *ASIP* and *AHCY* genes and the *ITCH* promoter region, which was identified as the genetic determinant of the white coat color of dominant white/tan (*A*^*Wt*^) agouti sheep; (ii) a single copy of *ASIP* with a silenced *ASIP* promoter was identified as the genetic cause of the recessive black sheep; and (iii) a single copy of wild-type *ASIP* (*A*^+^) with a functional promoter was identified as the genetic cause of the ancient Barbary sheep coat color (tan body and pale belly) [79]. Notably, a 9-bp deletion (D_9_) was detected in this study in exon 2 of *ASIP*. Collectively, the findings of this study showed the complexity of the mechanism underlying *ASIP* regulation and provided novel insights into its role in determining sheep coat color [79]. In addition, the copy number variation (CNV) in the *Agouti* locus was also used as a model for a segregation analysis to estimate allelic copy number [80].

Li et al. [81] performed a GWAS to identify the genetic determinants of the white vs. non-white coat color variation in a Finnsheep population. Among the 35 significant SNPs identified, 25 were located in or adjacent to five previously reported genes (*ASIP*, *TYRP1*, *KIT*, *MC1R*, and *MITF*) known to be involved in sheep coat color development. In addition, the *s66432.1* SNP in the *ASIP* gene showed the strongest association signal with the development of coat color patterns between white and non-white individuals. In particular, the white coat color pattern was associated with the *A* allele, whereas the non-white coat color pattern was associated with the *G* allele. Additional studies on the sheep *ASIP* gene are reported in Additional file 2: Table S8. Interestingly, with the emergence of gene-editing endonucleases, the *ASIP* gene was targeted using the CRISPR/Cas9-based knockout approach [82, 83]. Among the generated Merino sheep, three individuals harboring a 4-bp deletion in the *ASIP* gene showed (i) a badger-face with black body coat color (*n* = 2) and (ii) brown coat color with light ventral pigmentation (*n* = 1), whereas (iii) two individuals with a 20-bp deletion showed a black-white spotted coat color [83] (see Additional file 1: Fig. S3). Collectively, these findings led to the conclusion that the CRISPR/Cas9-mediated *ASIP* disruption together with D_9_/D_5_ mutations and *ASIP* duplications contributed to the emergence of coat color in *ASIP*-targeted white Merino sheep. Thus, the manipulation of the *ASIP* gene using gene-editing tools enabled the determination of its functional role in sheep coat pigmentation.

#### The *MC1R* gene

It was expected that various loci or genes would epistatically interact to generate a wide variety of sheep coat colors. In addition to the *Agouti* locus that encodes *ASIP*, the *Extension* locus encoding the *MC1R* gene was shown to play a role in the formation of coat color in sheep [84]. It plays the opposite role to *ASIP*, i.e., its high functional expression is responsible for the dark coat color phenotype. Våge et al. [17] analyzed the ovine *MC1R* gene in Norwegian Dala sheep, which comprise individuals with completely white or black coat colors. The study identified the complete co-segregation of *c.218 T*>*A* (p.73Met>Lys) and *c.361G*>*A* (p.121Asp > Asn) mutations with dominant black coat color (the dominant *E*^*D*^ allele indicates the black coat pattern and the wild-type *E*^+^ indicates the white coat pattern). When these two mutations are induced in the mice *MC1* receptor gene, the recapitulated p.73Met>Lys mutation alone was able to constitutively activate the receptor, whereas the p.121Asp >Asn mutation lacked this functionality, being thus proposedly required for high-affinity ligand binding [17]. In addition, Våge et al. [85] confirmed the co-segregation of these two mutations (the dominant *E*^*D*^ allele) with the black coat color pattern in Damara, Black Merino, and Black Corriedale. Moreover, Royo et al. [78] detected the presence of the dominant black *E*^*D*^ allele in Karakul, Black Merino, and Black Castellana sheep.

In addition, a GWAS was performed using the OvineSNP50K BeadChip to determine the major pigmentation gene using 42 samples obtained from Manchega and Rasa Aragonesa sheep, in which the solid black and solid white coat colors segregate [86]. In this study, the SNP *s26449* was the most strongly associated with coat pigmentation and the closest to *MC1R* [86]. In Brazilian Morada Nova sheep, a GWAS also revealed the occurrence of *MC1R* in the highly significant SNP-containing region [87]. In this study, white-coated and red-coated animals were homozygous for the *E*^+^ allele, whereas black-coated animals carried at least one copy of the *E*^*D*^ allele [87]. In a study performed to identify pigmentation-specific genes associated with skin photosensitization, *MC1R* was also the most significant genomic region under selection, and a number of other genes were also suggested to be potentially associated with this trait, including *KIT*, *PDGFRA*, *IRF4*, *SOX10*, *PICK1*, and *EDN3* [88]. Moreover, a GWAS using the OvineSNP600K Beadchip was performed on 75 Chinese Tan sheep individuals (29 black-headed and 46 white-coated; see Additional file 1: Fig. S4), which revealed two significant SNPs (*rs409651063* and *rs408511664*) in *MC1R* that were associated with coat color development [89]. Further information from previous studies on the sheep *MC1R* gene is shown in Additional file 2: Table S9. Collectively, these results highlight the role of *MC1R* and its variants in the development of coat color pattern in sheep.

#### The *TYRP1* gene

Considering the complexity of the coat color trait, it has been suggested that more than one gene could determine the phenotypic variation of coat colors. In Soay sheep, coat color density can be classified into dark and light patterns (in a ratio of approximately 3:1). Beraldi et al. [18] developed a complete linkage map to identify the loci responsible for determining several phenotypic traits, including coat color, which revealed *TYRP1* as a strong candidate gene influencing sheep coat color. Subsequently, Gratten et al. [90] analyzed *TYRP1* in Soay sheep, and showed that a nonsynonymous substitution (*c.869G*>*T*) in exon 4 was highly associated with coat color pattern. For this SNP, light-colored sheep were homozygous for *T*, whereas dark-colored sheep were either homozygous for *G* or heterozygous. In addition, no significant difference was found when analyzing *TYRP1* expression in dark- and light-coated Soay sheep, which hindered the assessment of the potential role of this SNP in determining sheep coat color [90]. Furthermore, an association between the *TYRP1 c.869G*>*T* mutation and body weight in Soay sheep has been reported [91]. It has been shown that dark-coated sheep with *GG* and *GT* genotypes were heavier at birth than light-coated sheep with the *TT* genotype. Moreover, dark-coated sheep with the *GG* genotype showed a fitness disadvantage compared to dark-coated sheep with the *GT* genotype. In this case, it was also difficult to predict how *TYPR1* influences the body weight or fitness [91]. It has been proposed that other genetic factors in the vicinity of *TYRP1* are likely responsible for microevolutionary constraints that influence these traits. It has also been suggested that the changing ratio between dark- and light-coated Soay sheep is a result of climate change [92, 93]; however, such a claim is not well evidenced [94].

In addition, Raadsma et al. [95] performed an extended QTL study on 13 skin and fiber pigmentation traits in Awassi × Merino × Merino backcross sheep. Significant QTL signals were identified on OAR2 where *TYRP1* is located. Notably, the nonsynonymous *TYRP1* SNP proposed by Gratten et al. [90] in Soay sheep was not confirmed in the examined flock. However, this study proposed an influence of the nonsynonymous SNP (*c.2240C*>*G*) in exon 2 of *TYRP1* on coat pigmentation [95]. In addition, two mutations, *g.80608128G*>*T* and *g.80611700C*>*T* within the *TYRP1* gene have been reportedly associated with brown coat color development in American sheep populations [96]. Furthermore, a GWAS was performed using Valais Red sheep with the aim to compare brown and black individuals, which showed a strong signal in the *TYRP1* locus, and three variants were shown to be associated with the recessively inherited brown coat color, namely (i) the previously reported missense variant (*c.869G*>*T*), (ii) a frame-shift variant (*c.86_87delGA*), and (iii) a nonsense variant (*c.1066C*>*T*) [97]. Taken together, these studies provide evidence for the potential involvement of *TYRP1* in the development of coat pigmentation in sheep. Additional information on sheep coat color variation is shown in Additional file 2: Table S10, which highlights the potential contribution of a set of genes to the formation of coat color in sheep.

#### Black bone color or hyperpigmentation

Black bone color or hyperpigmentation has been observed in few sheep breeds. In this condition, the pigment is found not only in the coat but also in the skin, muscles, inner organs, and bones. In the indigenous Chinese Nanping black-boned sheep, two synonymous mutations (*c.192G*>*C* and *c.462C*>*T*) in exon 1 of the *TYR* gene, as well as two synonymous mutations (c.12A>*G* and *c.144G*>*C*) in the *MC1R* gene were detected [98]. In addition, four combinations of a synonymous (*c.90C*>*T*) mutation and a missense (*c.203C*>*T*) mutation were found in the *TYRP1* gene in both black-boned and normal sheep [98]. Two other SNPs, a synonymous (*c.102C*>*T*) and a missense (*c.215T*>*C*) were also indicated in exon 2 of *TYRP1* in black-boned sheep [98]. Patterns of these SNPs showed elevated plasma tyrosinase activity [99–101]. In general, black-boned sheep show higher levels of melanin and related pigments in tissues and organs compared to the other examined breeds with white bone color [101]. Another gene, *EDN3*, known to cause hyperpigmentation in black-boned chickens (the silky fowl), was also analyzed in sheep [102]. However, the results of this study showed non-significant differences in genotype and allele frequencies of the identified SNPs and copy number of *EDN3* gene among black-boned and non-black-boned sheep. Interestingly, a different bone pigmentation, e.g., red pigmentation, has also been described in goats [103–105]. Such pigmentation characteristics might influence carcass quality and standards and, subsequently, consumer preference.

#### Fat color

Yellow fat color is an inherited recessive trait caused by the accumulation of carotenoids in adipose tissues [106–110]. This trait is known to influence carcass quality; thus it is of great interest to understand its genetic background. Våge et al. [111] analyzed two genes (*BCMO1* and *BCO2*) known to be important for carotenoid degradation in Norwegian sheep, and highlighted the potential of a nonsense mutation (*c.196C*>*T*) to be associated with the emergence of the yellow fat phenotype. However, individuals who lacked this mutation had yellow fat, which suggested that another mechanism might be involved in the emergence of this trait. To further explore this, a more recent investigation was conducted in Norwegian spælsau sheep with aberrations in the *BCO2* mRNA, which revealed the presence of a 7.9-kb insertion of an endogenous Jaagsiekte Sheep retrovirus (enJSRV) sequence into the first intron of *BCO2* [112]. This insertion led to the inactivation of *BCO2* due to the generation of a truncated product of 58 amino acids (29 from BCO2 and 29 from enJSRV), whereas the functional BCO2 product is known to contain 576 amino acids [112]. Interestingly, the ovine *BCO2* gene was previously targeted using the CRISPR/Cas9 gene-editing system in a study aimed at generating sheep with multiplex editing in three genes (*MSTN*, *ASIP*, and *BCO2*) [82, 113]. The *BCO2* gene was targeted to functionally validate its biological role, and was used as a gene with an obvious phenotype to validate the efficiency of CRISPR/Cas9 in sheep. The study revealed that the biallelic modification of *BCO2* resulted in yellow fat compared to the monoallelic and wild-type individuals, confirming its functional role in fat color formation in sheep [113].

### Genetic aspects of other phenotypic traits

#### Wool length

Several genes are known to influence sheep wool quality and quantity, e.g., those included in the *KAP* families [114–116]. In the following sections, we will focus on genes related to the wool phenotype rather than genes that influence quantitatively wool aspects. In addition to the *KAP* families, a number of genes were shown to greatly influence fiber traits, e.g., *FGF5*. The inactivation of the *FGF5* gene was first proven in mice to cause abnormally long hair [117]. Natural variants in the sheep *FGF5* gene causing the long hair phenotype have not yet been described [118]. However, the potential of *FGF5* has been investigated using gene modification tools. In order to induce wool growth by antagonizing the *FGF5* function, transgenic Chinese Merino sheep with ectopic expression of short alternative *FGF5* transcripts (FGF5s) were generated by zygotic injection of recombinant lentivirus; wool length was significantly longer in the transgenic group, and greasy wool weight was increased in yearling [119]. In addition, direct knockout of the *FGF5* was also achieved using the CRISPR/Cas9 system, and *FGF5*-disrupted sheep showed the desired long wool phenotype [120–123]. Interestingly, similar findings were also found in goats [124–126]. Using the *FGF5*-disrupted sheep model, a crosstalk between androgen and the *Wnt/β*-*catenin* signaling pathway in influencing wool increase and active hair-follicle density was revealed [123]. This interplay has been linked to the activation of the *c-MYC* and *KRT* genes associated with the inner root sheath. Furthermore, the *Shh* pathway being located downstream of the *Wnt/β*-*catenin* signaling pathway, it was also shown to be involved in wool length [123]. Moreover, a recent study on putative SNPs within the *FGF5* full-length gene in fine-wool sheep revealed potential molecular markers associated with wool length and greasy wool weight [127]. Based on these results, *FGF5* has been highlighted as a potential gene that could lead to improving yield in wool-producing sheep breeds, thus promoting the development of the wool industry.

#### Curly fleece

**Fig. 8:**
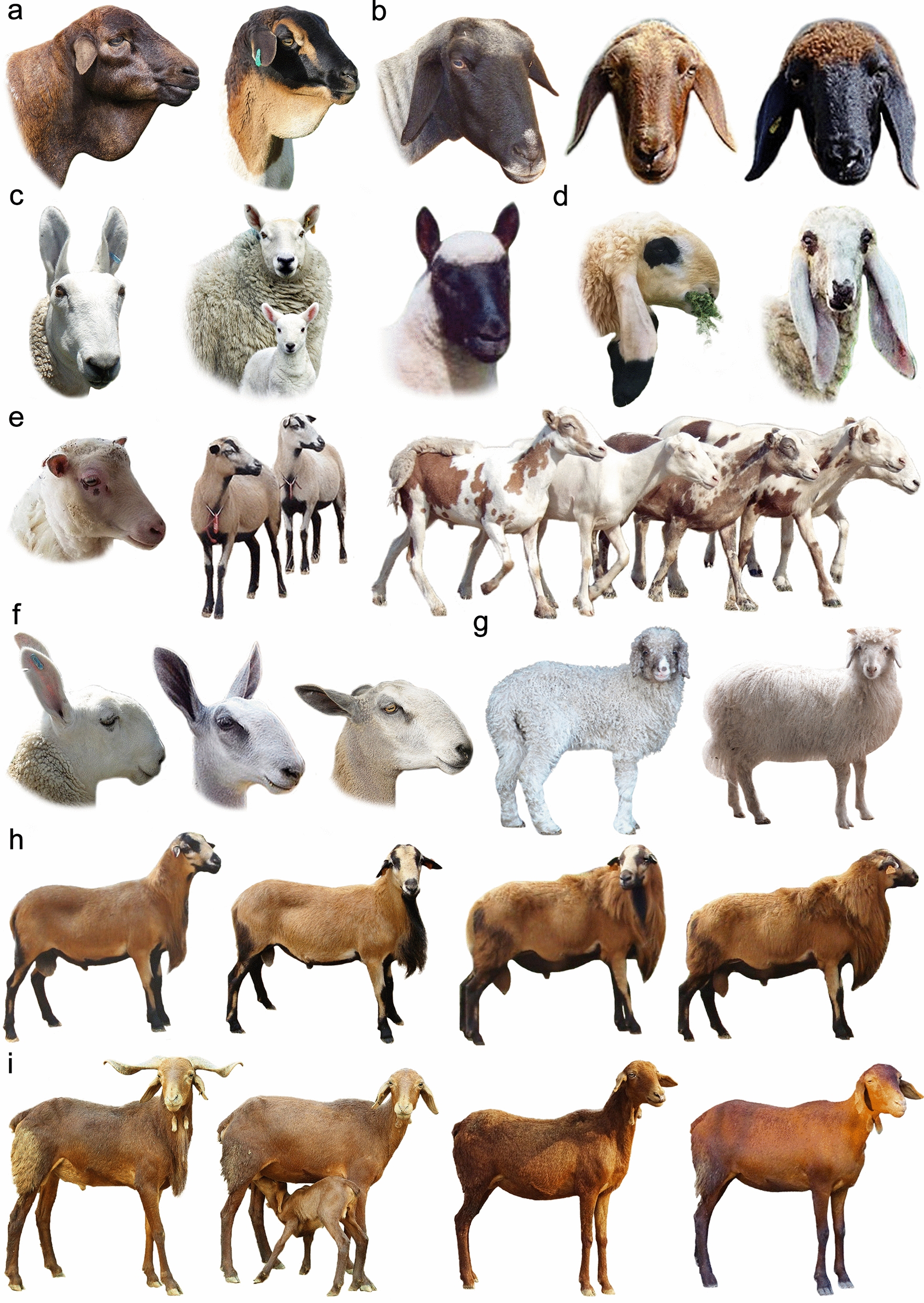
Other phenotypic traits in sheep. Representative examples of **a** dewlaps, **b** dropped ears, **c** pricked ears, **d** long ears, **e** short ears, **f** Roman noses, **g** curly fleeces, **h** manes, and **i** wattles. Photo credits and courtesies are shown in Additional file 2: Table S14

Chinese Tan sheep have curly fleece during the first months following birth (Fig. 8g), and this phenotype gradually disappears with aging. To understand this phenotypic change, a transcriptomic study involving sheep at two different developmental stages was performed, which enabled the identification of 600 differentially-expressed genes (DEG) [128]. The top 20 highly expressed transcripts belonged to genes included in *KAP* families as well as other genes, namely, *KAP3*.2, *KAP2*.3, *KRT25*, *KAP1.1*, *KRT5*, *KRT71*, *KRT14*, *KRT2.11*, *KRT83*, *KRT31*, *KRT34*, *KRT33*, *KRT27*, *KRT33B*, *TCHH*, *RPS8*, *PRR9*, *CST6*, *ASIP*, and *CO3*. Furthermore, isoform analysis revealed that the *MT3* isoform was upregulated in lamb skin compared to adult individuals, suggesting its potential involvement in the development of curly fleece phenotype [128]. In addition, using an integrated miRNA-mRNA analysis of samples obtained from sheep at these two physiological stages, 49 differentially-expressed (DE) miRNAs were identified [129]. In addition, 36 potential miRNA-mRNA target pairs were identified (including 25 DE miRNAs and 10 DEG). Among DE miRNAs, it was confirmed that *miR-432* could inhibit *KRT83* expression [129]. Moreover, using suppression subtractive hybridization analysis, 67 DEG were identified in the samples of sheep at two developmental stages, and *KRT71* was significantly highly expressed in individuals within the curly fleece group compared to the non-curling group [130]. The *MFZ1* transcription factor has also been suggested to regulate the expression of *KRT71* [130]. It has also been suggested that *KRT83* expression was higher in Chinese Tan lambs with curly fleece compared to adults, and that the expression of the *CAP1* transcription factor was low [131]. When *CAP1* was overexpressed, *KRT83* expression was repressed, which suggests that *CAP1* has a role in the regulation of *KRT83* expression [131].

In a genome-wide detection of CNV on 48 Chinese Tan sheep, a CNV region overlapping the homeobox transcription factor *DLX3* was suggested as a candidate CNV for the curly fleece phenotype [132]. SNPs (*c.118T*>*C*, *c.228T*>*C, c.688A*>*G*, and *c.1,038_1,039 insC*) within the 3′-UTR of *DLX3* were also previously described as associated with wool crimp in sheep [133]. In Chinese Merino sheep, a set of genes (namely, *CDC6*, *RARA*, *IGFBP4*, and *GHR*) was identified as candidates for crimp number, in particular *IGFBP4* [134]. In addition, the levels of *PAPPA2* expression in dermal papilla cells (DPC) of straight-wool Hu lambs was significantly higher than in DPC of curved wool Hu lambs [135]. It has been speculated that *PAPPA2* affects the proliferation of DPC by regulating *IGFBP5* expression, which influences coat phenotypic patterns [135]. In Hu sheep, *miR-143* has been speculated to influence wool curvature by targeting *CUX1* thereby inhibiting the proliferation of DPC and *KRT71*, which ultimately regulates the growth and development of hair follicles [136]. The higher expression of *CUX1* and *KRT71* was proposed to be related to wool curvature [136]. Furthermore, an analysis of DNA methylation levels in Chinese Tan sheep at three growth stages (1-, 24-, and 48-month old) revealed 51 differentially methylated genes (DMG) associated with age growth and curly fleece formation [137]. Among these DMG, *KRT71* and *CD44* were highly methylated in one-month-old lambs, whereas *ROR2* and *ZDHHC13* were highly methylated in 48-month-old sheep. Interestingly, a similar phenotype of curly fibers was also found in goats [138–141]. Further information on the curly fleece phenotype in sheep is provided in Additional file 2: Table S11. Several genes and small regulatory RNAs were proposed to be involved in the formation of the curly fleece phenotype. Understanding the genetic basis of wool-related traits in sheep is of significant importance for the genetic customization of wool characteristics and quality for economic purposes.

#### Fine vs. coarse fleece

Wool fiber diameter varies widely among sheep breeds. Fine wool fiber is characterized by a small diameter, whereas coarse wool fiber has a larger diameter compared to fine fiber. Fine wool is employed in manufacturing soft clothes, whereas coarse wool is employed in carpet weaving. As it is significantly determinant for wool characteristics, early research efforts have concentrated on understanding the genetic determinants leading to differences in wool fiber diameter [142–145]. In a GWAS using 765 Chinese Merino sheep, nine significant SNPs were suggested to be associated with wool fiber diameter; among these, four SNPs were located within previously known or predicted genes, including *TSPEAR*, *PIK3R4*, *KRTCAP3*, and *YWHAZ* [146]. These genes were found to be directly or indirectly associated with skin development, and their role in wool follicle development and fiber diameter has been proposed. To determine the wool fiber diameter coefficient of variation, three significant SNPs were detected, among which one was found to be located within the *KIF16B* gene [146]. In addition, when 18 markers under selection in fine-wool Australian Merino and coarse-wool Spanish Churra sheep breeds were analyzed, divergent variants potentially involved in phenotypic variation of wool type, growth, and meat production/quality traits were identified [147]. However, in this study, the investigated two breeds had varied phenotypic traits (wool, color, carcass, milk production, parasite resistance, among others), thus the identified markers under selection are likely not exclusively related to wool traits [147]. In addition, in another study, *BNC1* has been suggested as significantly associated with the mean fiber diameter in Chinese Merino sheep [134].

To further understand wool fiber diameter at the transcriptome level, a study was conducted using Gansu Alpine Merino sheep, which have wool of two fiber diameters (super-fine and fine), were analyzed, and 40 DEG were identified [148]. Also, when analyzing natural antisense transcripts, three of 40 DEG (*AQP8*, *Bos_d2*, and *SPRR*) had significant natural antisense transcripts and were all significantly downregulated in the investigated super-fine wool group, thus indicating the correlation between gene transcripts and natural antisense transcripts in gene expression regulation [148]. Additional information on wool diameter is provided in Additional file 2: Table S12. Collectively, the findings discussed above point towards the existence of sets of potential genes that regulate wool fiber diameter, but further studies are required to elucidate their underlying genetic mechanism.

#### Hairy vs. woolly fleece

Several modern domestic sheep breeds have been selected for short and woolly fleece coat phenotype, which differs substantially from the ancestral long and hairy fleece coat phenotype. To identify the molecular basis of this evolutionary phenotypic variation, Demars et al. [149] analyzed the Romane sheep breed in which fleece pattern segregates, to determine the causal mutation that controls fleece variation. An insertion of an antisense *EIF2S2* retrogene (*asEIF2S2*; ~ 1.5 kb) into the 3′-UTR of *IRF2BP2* was identified as leading to the production of an abnormal *IRF2BP2*/*asEIF2S2* transcript. This chimeric messenger targets the genuine sense of *EIF2S2* RNA and alters the expression of both *EIF2S2* and *IRF2BP2* mRNA by a retroposition mechanism-led RNA-RNA hybridization. It was shown that woolly sheep carried an insertion (*IRF2BP2*^*asEIF2S2*^ allele), whereas hairy sheep carried the ancestral *IRF2BP2*^*wt*^ allele [149]. This study provided the keys to understand the unique mechanism that underlies a causal variant leading to phenotype variation related to fiber folliculogenesis in sheep [150]. Lv et al. [151] identified a novel mutation in OAR25 (*g.7068586T*>*C*) in the 3′-UTR of *IRF2BP2* as a causal variant influencing fleece fiber diameter. In addition, the expression of a bindable miRNA (*oar-miR-20a-3p*) was detected exclusively in the skin tissues of coarse-wool sheep carrying the ancestral *T* allele. Furthermore, a strong selection marker in the secondary wool follicle-associated *VEGFA* gene was detected, which was shown to be co-activated by *IRF2BP2*. It has been proposed that *oar-miR-20a-3p* binds to the wild-type 3′-UTR of *IRF2BP2*, regulating its expression and subsequently inhibiting *VEGFA* expression in the skin tissues of coarse-wool sheep [151].

Interestingly, when genotyping the hairy/woolly locus in a ~ 1600-year-old naturally mummified sheep found in a salt mine (*Chehrābād*) in northwestern Iran, the ancestral “hairy” allele (likely *IRF2BP2*^*wt/wt*^ or *IRF2BP2*^*wt/asEIF2S2*^) was identified [152]. In addition, using scanning electron microscopic observations of the mummified hair fibers showed characteristics that are specific to sheep hair fibers of mouflon and medium-wool breeds. This suggested that, during that ancient period, the herd was maintained for meat or milk production rather than wool extraction, and ovicaprids were destined to be used as food source for workers in that location [152]. This study highlighted the potentiality of using a locus for the phenotypic characterization of ancestral breeds using ancient DNA. Fleece pattern in sheep influences adaptation to environmental conditions, thus it is expected that the discovered variants related to different fleece patterns will be used in selection- or molecular genetics-based strategies to customize sheep breeds in order to better adapt to future environmental changes and sustainable sheep production.

#### Ear size

Sheep ear morphology varies widely in terms of length (large, medium, small or “microtia”, and earless or “anotia”) and erectness (pricked or “erect” and dropped or “floppy”), and is considered a sheep breed determinant (Fig. 8b–e). In sheep breeds with varying ear length, such as Awassi, sheep farmers prefer to breed individuals with the normal large ear phenotype owing to their better physical constitution. In addition, it is indicated that earless Awassi sheep tend to show a more flighty behavior, which likely results from impaired hearing ability [153]. Ear phenotype has also been associated with environmental adaptation, which highlights the importance of investigating the molecular basis of the ear phenotype in sheep. A SNP within the *MSRB3* gene was to strongly segregate within the Chinese Duolang sheep with large ear phenotype and not in other breeds with normal or smaller ears, thus suggesting a role for *MSRB3* in determining the ear phenotype in sheep [11]. In a GWAS performed to investigate microtia in Jordanian Awassi sheep (in which 12 normal-eared and 8 earless individuals were included), a SNP adjacent to *GATA6* was identified [153]. *GATA6* was proposed based on its location and function, which is related to developmental processes associated with outer ear shaping (e.g., chondrogenesis, the process formation of cartilage) [153].

To further investigate genes that influence the ear phenotype in Duolang sheep, a GWAS was carried out using 115 adult individuals with different ear sizes [154]. The most significant SNP was located within the *DCC* gene which is known to be associated with ear development; two other significant SNPs (*PTPRD* and *SOX5*) showed a physical association with ear development. In this study, no association with *GATA6* was found [154]. When genotyping 40 individuals (20 with microtia and 20 normal) of the Valle del Belice sheep breed, a strong marker within intron 3 of *CLRN1* was identified [155]. *CLRN1* is associated with Usher syndrome type 3A, which is a progressive hearing and vision loss, and was thus proposed as a novel candidate gene for determining microtia in sheep [155]. In another GWAS using Chinese Altay sheep (26 microtia individuals and 29 normal-ear size individuals) combined with direct genotyping of a larger number of individuals, a 76-bp duplication in the evolutionarily conserved region (ECR) of *HMX1* was determined [156]. This gene is known to be associated with a congenital deformity of the outer ear in species such as cattle and rats, and was proposed to determine microtia in sheep [156]. Furthermore, by analyzing previously studied genes in 84 Awassi sheep (16 earless, 41 short-eared, and 27 normal-eared), the ECR 76-bp duplication of *HMX1* and a missense SNP (*g.34498242A*>*G*) in exon 7 of *GATA6* were shown to be significantly associated with microtia [157]. In addition, using a pruned dataset of 515 sheep from 17 breeds with different ear phenotypes, the most relevant marker for determining sheep ear size was found within the previously described *MSRB3* gene [158]. The strongest markers for ear erectness (dropped vs. pricked ears) were also located within the 3’-region of *MSRB3* [158]. Potential introgressive haplotypes of *MSRB3* were found to be associated with ear morphology [55]. Xu et al. [159] reported potential novel genes associated with ear size, which included *PTCH1* and *EMX2*. Information on genetic variants known to determine the ear phenotype can be used in genetic screening to improve the selection against undesirable ear phenotypes (e.g., microtia) in certain sheep breeds.

Interestingly, other regions (including e.g., the *SUPT3H*, *STXBP5L*, *DENND1A*, and *VPS13B* genes) that have been introgressed from wild *Ovis* species into domestic sheep were suggested to be potentially associated with facial shape traits (e.g., nose bridge breadth, nose morphology, chin dimples, and forehead protrusion) [55].

#### Number of nipples

The supernumerary nipple/teat phenotype (also known as polythelia; see Additional file 1: Fig. S5a–c) was observed in sheep and documented over a century ago [160–166]. Ewes with supernumerary nipples usually carry three, four, five, or six nipples, and extra nipples are extremely small and undeveloped. In the past, extra-nippled ewes were believed to be more fertile, and selective breeding for supernumerary nipples could have been developed to ultimately lead them from their rudimentary condition to fully functional milk-producing nipples [160]. However, it has been suggested that this trait has no practical value in sheep production [165], and now it is considered a morphological defect that might impede milking efficiency by increasing milking time and the risk of injuries [167], as well as negative implications for overall udder health [168]. A GWAS was performed using 144 ewes of the Chinese Wadi breed (75 cases with four teats and 69 control cases with two teats), which revealed 63 significant SNPs and a set of functional genes (*BBX*, *SEMA3D*, *IRF2BP2*, *SETBP1*, *NAV3*, *CD47*, *LRP1B*, *CERS5*, *CASK*, and *CADM2*); a significant SNP near the *BBX* and *CD47* genes was determined to be highly associated with the supernumerary nipple phenotype [169]. In addition, Gene Ontology (GO) enrichment analysis revealed a set of functional genes that are potentially involved in determining nipple number and development, including *CASK*, a gene that was characterized as a risk factor associated with breast cancer development [169].

In addition to the five genes associated with breast cancer (*LRP1B*, *GRM3*, *MACROD2*, *SETBP1*, and *GPC3*) [169], seven novel genes (*PHGDH*, *KDM3A*, *GLIS3*, *FSHR*, *CSNV2*, *CSN1S1*, and *ROBO2*) previously reported in mice were associated with mammary and nipple development in sheep [27]. Furthermore, by performing GWAS using CNV data, the associated functional gene, *GPC5*, was found to be associated with nipple number [27]. In Hu sheep, *LHFP*, *DPYSL2*, and *TDP-43* genes were also found to be related to nipple number [170]. The identification of genes and variants that control nipple number trait will likely enable the molecular characterization of sheep breeds for the desired nipple phenotype.

#### Number of thoracic and lumbar vertebrae

Vertebrae are the interlocking bones that constitute the spinal cord. Sheep, as a tetrapod, have five types of vertebrae, namely cervical, thoracic, lumbar, sacral, and coccygeal or caudal vertebrae (see Additional file 1: Fig. S6). In addition to studies performed to identify genes associated with caudal vertebrae in sheep [14, 33], it has been suggested that the number of thoracolumbar vertebrae is associated with carcass length and weight [171, 172]. Thus, it is of interest to understand the genetic basis of vertebrae-related traits. Six hundred Chinese Kazakh sheep (400 with a larger number of thoracic vertebrae and 200 with an average number of thoracic vertebrae) were investigated by DNA sequencing. Eleven putatively selected genomic regions related to the indicated phenotype were revealed, including the vertebral development-related *VRTN* gene and the *HOXA* gene family [173]. Further investigation showed that *VRTN* is the major promising gene involved in the determination of the number of thoracic vertebrae in sheep. In addition, during fetal development, the expression level of *VRTN* was shown to be significantly higher in sheep with more thoracic vertebrae compared to sheep in the control group [173].

A recent study identified genes associated with the number of ribs in 36 Hu sheep with 14 ribs (supernumerary condition) and 36 Hu sheep with 13 ribs (normal condition), and a combined effect of *CPOX* (*g.178730623T*>*G*; Oar_rambouillet_v1.0), *KCNH1* (*g.75716237C*>*G*), and *CPQ* (*g.88323841A*>*G*) was proposed to be associated with rib number [174]. Furthermore, using whole-genome resequencing of Chinese Duolang sheep with a different number of lumber vertebrate (6 vs. 7) revealed a significant region under selection (49.68–49.74 Mb; Oar_v4.0) that is contained within the *SFRP4* gene [175]. In addition, two SNPs (*rs600370085C*>*T* and *rs415133338A*>*G*) within this gene were significantly associated with multi-lumbar vertebrae [175]. Additional file 2: Tables S13 and S14 contains further information on SNPs associated with the number of thoracic and lumbar vertebrae in sheep. Increasing the understanding of genes and variants controlling this phenotype in sheep will enable the design of selective breeding programs for increasing carcass length and weight.

#### Body height and stature

Dwarfism, whether proportionate or disproportionate, is included among the conditions that influence sheep phenotype [176, 177]. Ancon sheep is one of the breeds known to be affected by dwarfism, as a result of a putative dwarf mutation, leading to individuals with elongated bodies and very short legs [178]. This phenotype was subjected to selection by breeders since such affected sheep are unable to jump fences. Ancon sheep were raised for a brief period of time, but went extinct as their phenotype was no longer desired [179, 180]. Another physical condition including chondrodysplasia, characterized by dwarfism and angular deformities of forelimbs, was reported in Texel as a result of a 1-bp deletion of *T* (*g.25513delT*) at position 107 in exon 3 of the *SLC13A1* gene [181]. Another condition of dysplasia was also reported in the Brazilian Cabugi sheep but its potential genetic basis has not been elucidated; in this context, dwarf lambs are characterized by a small size, short legs, domed heads, and other abnormalities [182]. The spider lamb syndrome that is associated with severe skeletal deformities (e.g., abnormal spines and long splayed legs) is characterized by a loss-of-function nonsynonymous T>A transversion (p.700Val>Glu) in the *FGFR3* gene [183]. Subsequently, it has been proposed that the decreased expression of *FGFR3* increased the proliferation and growth of plate chondrocytes by increasing telomerase reverse transcriptase (TERT) expression levels in vitro, suggesting a potential mechanism for skeletal overgrowth in vivo [184]. In another report, it has been proposed that heterozygosity of the inactivated *FGFR3* gene was used to improve skeletal growth [185]. Furthermore, several genomic analyses identified a selective sweep on OAR6 harboring the *LCORL* locus that is implicated in determining height and stature in sheep [186–193].

#### Generic notes on other phenotypic traits

Other phenotypic traits in sheep are not yet well understood at the genetic level. Among these are included belly bareness (referring to the naturally bare area of skin on the belly [194, 195]), breech bareness (referring to the area of naturally bare skin around the perineum [196–198]), skin wrinkles (the folds or creases in the skin [199–201]), albinism (the congenital absence of any pigmentation [202, 203]), dewlap (a fold of loose skin which hangs from the neck or the throat as found in Persian sheep; Fig. 8a), manes (the growth of long hair on the neck as found in Barbados Blackbelly sheep; Fig. 8h), wattles (skin appendages found in the cervical region [204, 205] as found in Madras Red sheep; Fig. 8i), udder conformation [206], and other wool-related traits [207, 208].

Other significant traits for which the genes involved are well known have a great influence on sheep phenotype. These include, e.g., *MSTN* that causes increased body weight by generating the double-muscle phenotype [209–211], *callipyge* that causes the muscular rump phenotype [212–214], and *SOCS2* that is associated with body weight and size [215, 216]. Since these traits are production traits rather than phenotypic traits, we have not presented them in detail in this review. Collectively, it can be stated that all the above-mentioned, well-validated genes and their variants function in an orchestrated manner to give rise to the phenotypic variation among sheep breeds.

## Concluding remarks

In the present review, several genes known to be or potentially involved in determining sheep phenotype variation have been discussed. Understanding the genetic basis of phenotypic trait variation in sheep is of great importance, since these are breed determinants and highly linked with production and welfare traits. In spite of the number of genes discussed here, the genetic link and expression patterns of several of these genes are not yet well understood. Further studies are required to identify novel genomic loci and genes for traits for which the genetic determinism has not yet been described. The identification of genes, their causative variants, and their expression characteristics will help to improve sheep breeding through genomic-based selection and modern molecular genetic tools.

## Supplementary Information

**Additional file 1: Figure S1.** High-throughput sequencing outcomes for candidate genes linked with the sheep tail phenotype pattern. Outcomes of genomic **a** and transcriptomic **b** approaches. **Figure S2.** An example of coat color variation in sheep. Coat colors and markings in Shetland sheep breed. Poster copyright and published with the consent of the *Shetland Sheep Society* (www.shetland-sheep.org.uk). **Figure S3.** CRISPR/Cas9-generated *ASIP* knockout Chinese Merino sheep. **a** Badger-face with black body coat color. **b** Brown coat color with light ventral pigmentation. **c** Black-white spotted coat color. **d** Wild-type phenotype. Figure adapted from Zhang et al. [83]. **Figure S4.** Phenotypic variations of face coat color in sheep. A representative example from Chinese Tan sheep with varied face coat colors, including white, light brown spots, dark brown spots, brown, black spots, black marks, and black. Photos were taken by P.K. at Ningxia Tianyuan Tan Sheep Farm, Hongsibu, China. **Figure S5.** Phenotypic variations of nipple (teat) number in sheep. A representative example from Chinese Tan sheep, including **a** normal nipple number (a pair of nipples) and supernumerary nipples [**b** with three nipples and **c** four nipples]. Photos were taken by P.K. **Figure S6.** Location of vertebrae in the sheep skeleton. This includes cervical, thoracic, lumbar, sacral, and coccygeal vertebrae (item source: PNGITEM).

**Additional file 2: Table S1.** Additional genomic investigations on sheep tails [159, 218–226]. **Table S2.** Sheep tails from a transcriptomic perspective: mRNA profiles [4, 12, 14, 20, 21, 219, 227–241]. **Table S3.** Sheep tails from a transcriptomic perspective: microRNA profiles [242–254]. **Table S4.** Sheep tails from a transcriptomic perspective: lncRNA profiles [255–259]. **Table S5.** Sheep tails from a proteomic perspective [260–262]. **Table S6.** Gene expression and association studies of sheep tails [241, 263–294]. **Table S7.** Additional genetic investigations on sheep horn phenotype [295–299]. **Table S8.** Additional genetic studies on sheep coat color phenotype: The *ASIP* gene [300–306]. **Table S9.** Additional genetic studies on sheep coat color phenotype: The *MC1R* gene [89, 302, 304–309]. **Table S10.** Additional genetic studies on sheep coat color variation [310–327]. **Table S11.** Additional studies on the curly fleece phenotype in sheep [132, 146, 328–346]. **Table S12.** Additional studies of the fine vs. coarse fleece phenotype in sheep [138, 333, 334, 347–358]**. Table S13.** Potential genes linked to the occurrence of the number of thoracic and lumbar vertebrae in sheep [172, 359–362]. **Table S14.**Credits and courtesies of photos used in the current review article.

## Data Availability

Not applicable.
