# Supplementary material for: Genetics of the phenotypic evolution in sheep: a molecular look at diversity-driving genes
Source: Genet Sel Evol. 2022 Sep 9;54:61. doi: 10.1186/s12711-022-00753-3 (PMC9463822; doi:10.1186/s12711-022-00753-3)
Supplement: Supplementary file 1 — Additional file 1: Figure S1. High-throughput sequencing outcomes for candidate genes linked with the sheep tail phenotype pattern. Outcomes of genomic a and transcriptomic b approaches. Figure S2. An example of coat color variation in sheep. Coat colors and markings in Shetland sheep breed. Poster copyright and published with the consent of the Shetland Sheep Society (www.shetland-sheep.org.uk). Figure S3. CRISPR/Cas9-generated ASIP knockout Chinese Merino sheep. a Badger-face with black body coat color. b Brown coat color with light ventral pigmentation. c Black-white spotted coat color. d Wild-type phenotype. Figure adapted from Zhang et al. [83]. Figure S4. Phenotypic variations of face coat color in sheep. A representative example from Chinese Tan sheep with varied face coat colors, including white, light brown spots, dark brown spots, brown, black spots, black marks, and black. Photos were taken by P.K. at Ningxia Tianyuan Tan Sheep Farm, Hongsibu, China. Figure S5. Phenotypic variations of nipple (teat) number in sheep. A representative example from Chinese Tan sheep, including a normal nipple number (a pair of nipples) and supernumerary nipples [b with three nipples and c four nipples]. Photos were taken by P.K. Figure S6. Location of vertebrae in the sheep skeleton. This includes cervical, thoracic, lumbar, sacral, and coccygeal vertebrae (item source: PNGITEM). [file 12711_2022_753_MOESM1_ESM.pdf]

Additional file 1

Genetics of the Phenotypic Evolution in Sheep: A Molecular Look at Diversity-driving Genes

Peter Kalds[1,2], Shiwei Zhou[1,3], Yawei Gao[1], Bei Cai[1], Shuhong Huang[1], Yulin Chen[1,4*] & Xiaolong Wang[1,4*]

[1]Key Laboratory of Animal Genetics, Breeding and Reproduction of Shaanxi Province, College of Animal Science and Technology, Northwest A&F University, Yangling 712100, China.

[2]Department of Animal and Poultry Production, Faculty of Environmental Agricultural Sciences, Arish University, El-Arish 45511, Egypt.

[3]College of Veterinary Medicine, Northwest A&F University, Yangling 712100, China.

[4]International Joint Agriculture Research Center for Animal Bio-breeding, Ministry of Agriculture and Rural Affairs, Yangling 712100, China.

[*]Corresponding authors: Yulin Chen chenyulin@nwafu.edu.cn; Xiaolong Wang xiaolongwang@nwafu.edu.cn.

Supplementary figures

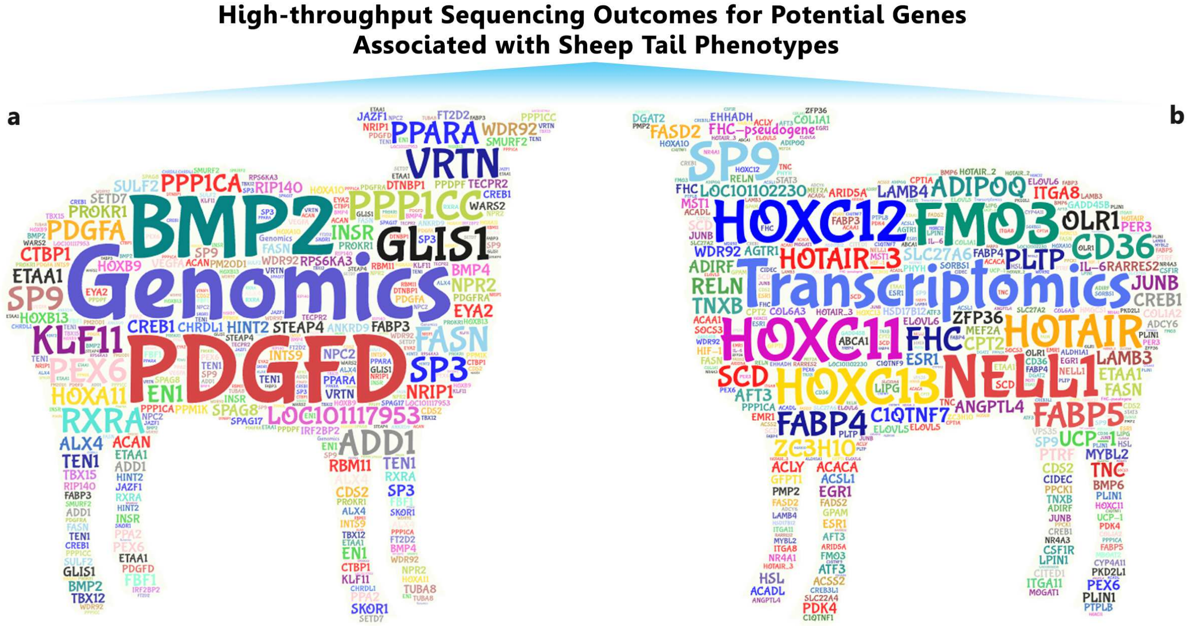

Supplementary fig. S1. High-throughput sequencing outcomes for candidate genes linked with the sheep tail phenotype pattern. Outcomes of genomic (a) and transcriptomic (b) approaches.

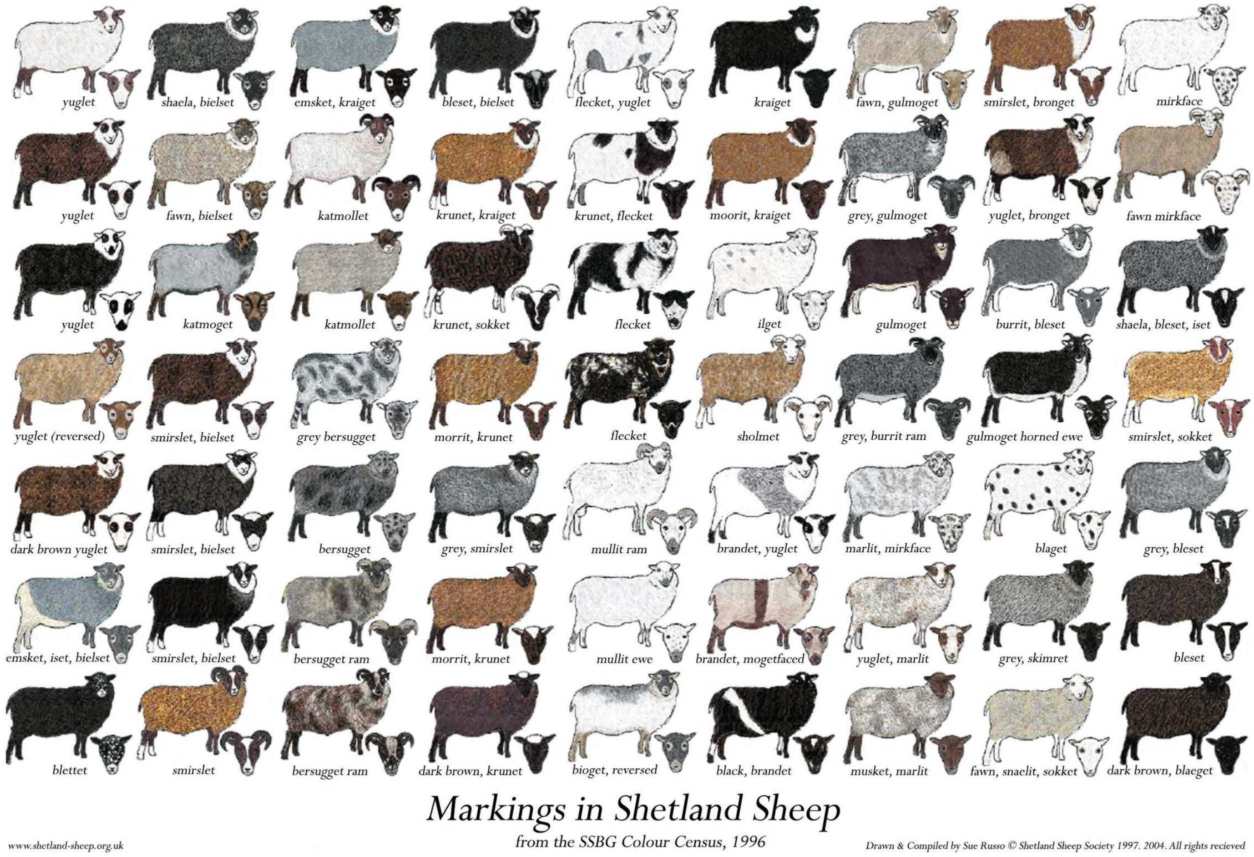

Supplementary fig. S2. An example of coat color variation in sheep. Coat colors and markings in Shetland sheep breed. Poster copyright and published with the consent of the *Shetland Sheep Society* (www.shetland-sheep.org.uk).

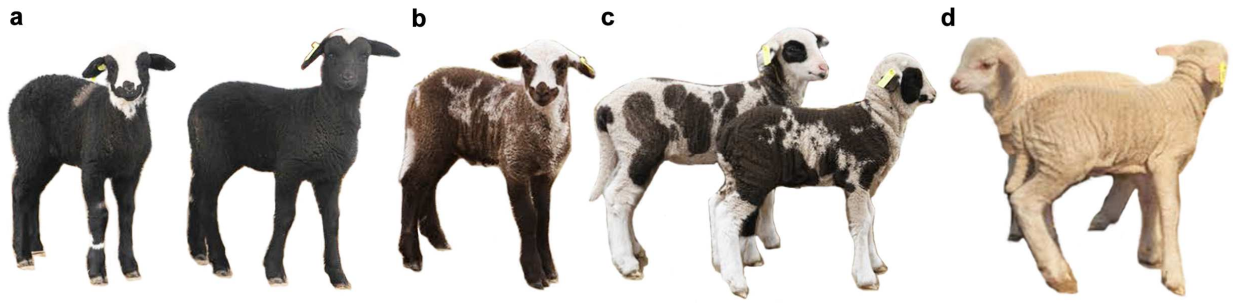

Supplementary fig. S3. CRISPR/Cas9-generated *ASIP* knockout Chinese Merino sheep. (a) Badger-face with black body coat color. (b) Brown coat color with light ventral pigmentation. (c) Black-white spotted coat color. (d) Wild-type phenotype. Figure adapted from Zhang et al. [83].

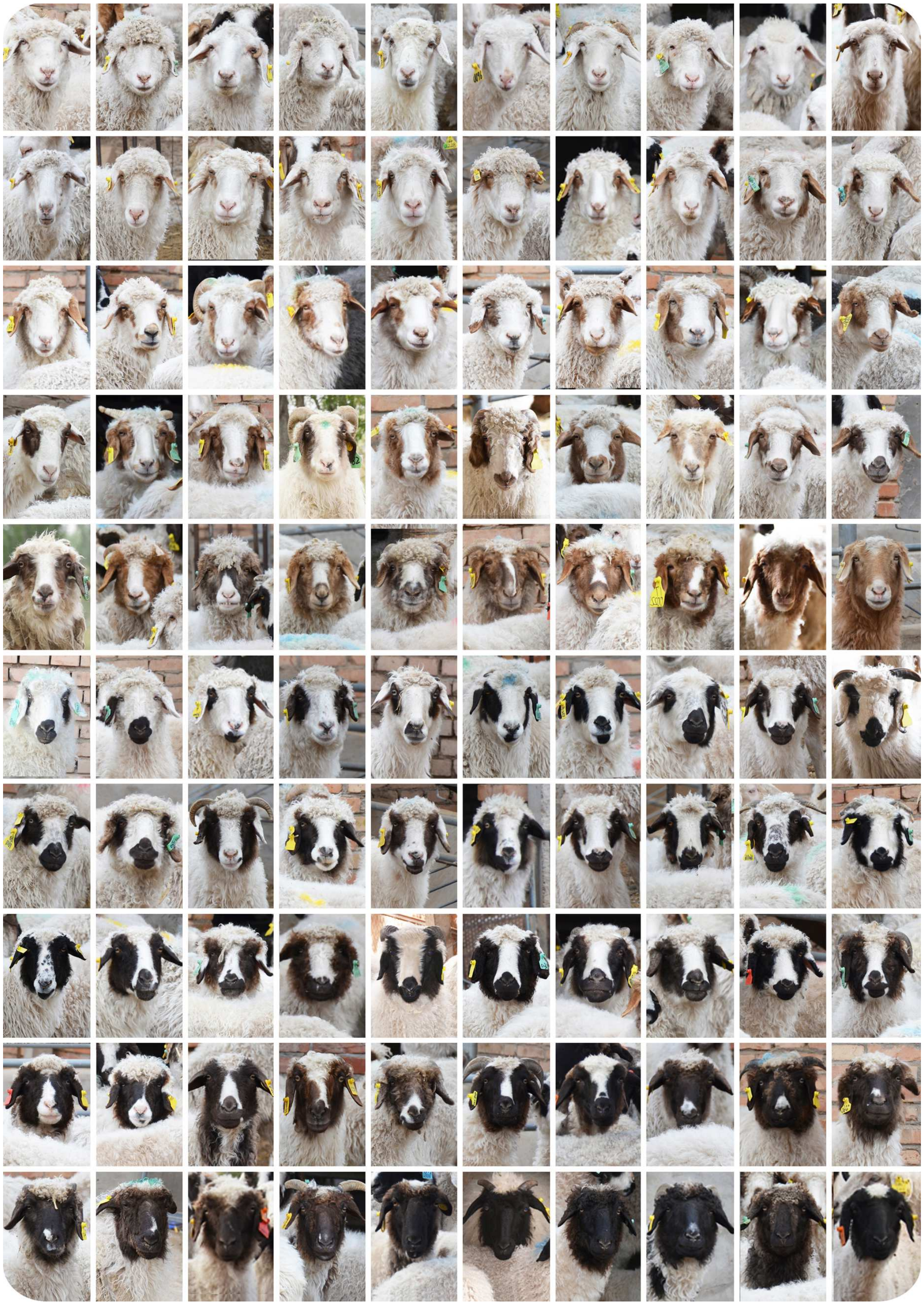

Supplementary fig. S4. Phenotypic variations of face coat color in sheep. A representative example from Chinese Tan sheep with varied face coat colors, including white, light brown spots, dark brown spots, brown, black spots, black marks, and black. Photos were taken by P.K. at Ningxia Tianyuan Tan Sheep Farm, Hongsibu, China.

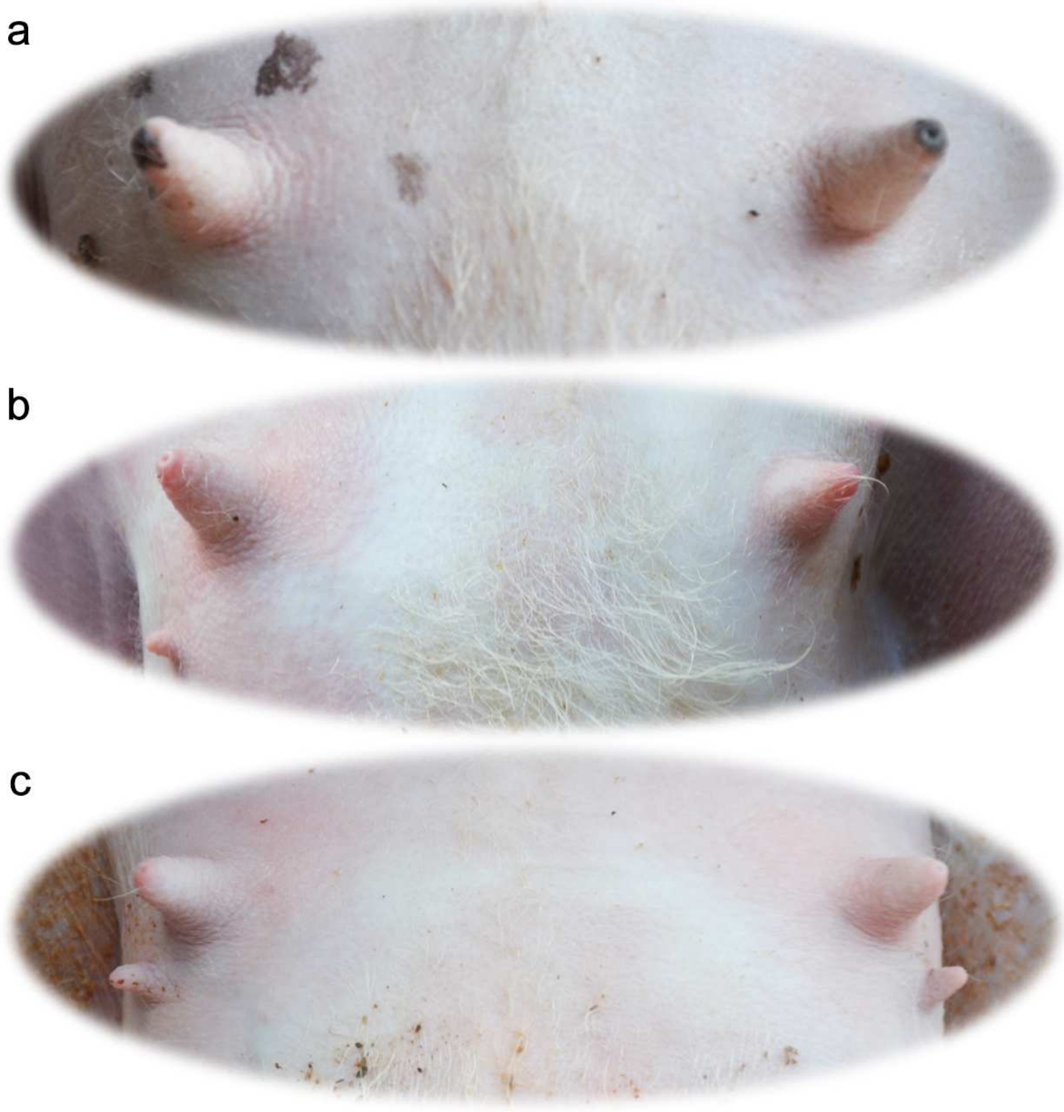

Supplementary fig. S5. Phenotypic variations of nipple (teat) number in sheep. A representative example from Chinese Tan sheep, including (a) normal nipple number (a pair of nipples) and supernumerary nipples [(b) with three nipples and (c) four nipples]. Photos were taken by P.K.

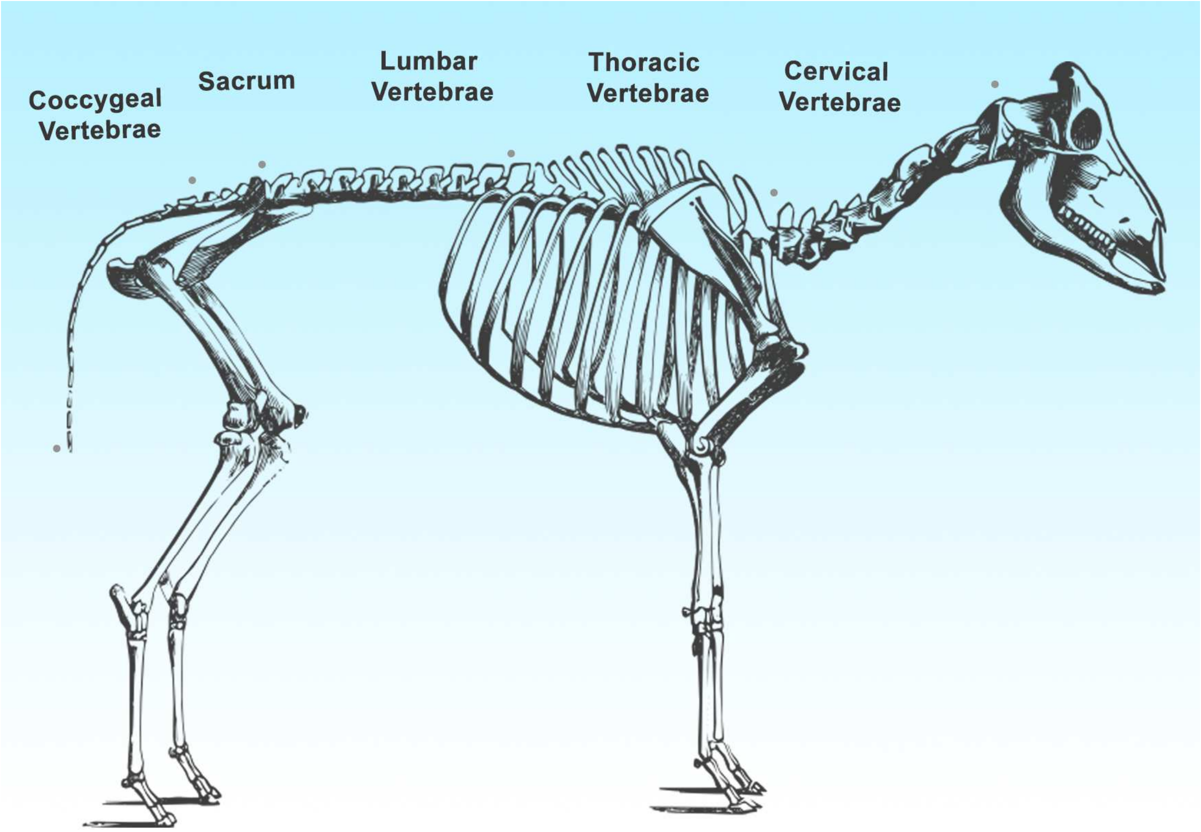

Supplementary fig. S6. Location of vertebrae in the sheep skeleton. This includes cervical, thoracic, lumbar, sacral, and coccygeal vertebrae (item source: PNGITEM).
