# Supplementary material for: Genetics of the phenotypic evolution in sheep: a molecular look at diversity-driving genes
Source: Genet Sel Evol. 2022 Sep 9;54:61. doi: 10.1186/s12711-022-00753-3 (PMC9463822; doi:10.1186/s12711-022-00753-3)
Supplement: Supplementary file 2 — Additional file 2: Table S1. Additional genomic investigations on sheep tails [159, 218–226]. Table S2. Sheep tails from a transcriptomic perspective: mRNA profiles [4, 12, 14, 20, 21, 219, 227–241]. Table S3. Sheep tails from a transcriptomic perspective: microRNA profiles [242–254]. Table S4. Sheep tails from a transcriptomic perspective: lncRNA profiles [255–259]. Table S5. Sheep tails from a proteomic perspective [260–262]. Table S6. Gene expression and association studies of sheep tails [241, 263–294]. Table S7. Additional genetic investigations on sheep horn phenotype [295–299]. Table S8. Additional genetic studies on sheep coat color phenotype: The ASIP gene [300–306]. Table S9. Additional genetic studies on sheep coat color phenotype: The MC1R gene [89, 302, 304–309]. Table S10. Additional genetic studies on sheep coat color variation [310–327]. Table S11. Additional studies on the curly fleece phenotype in sheep [132, 146, 328–346]. Table S12. Additional studies of the fine vs. coarse fleece phenotype in sheep [138, 333, 334, 347–358]. Table S13. Potential genes linked to the occurrence of the number of thoracic and lumbar vertebrae in sheep [172, 359–362]. Table S14.Credits and courtesies of photos used in the current review article. [file 12711_2022_753_MOESM2_ESM.pdf]

Additional file 2

Genetics of the Phenotypic Evolution in Sheep: A Molecular Look at Diversity-driving Genes

Peter Kalds[1,2], Shiwei Zhou[1,3], Yawei Gao[1], Bei Cai[1], Shuhong Huang[1], Yulin Chen[1,4*] & Xiaolong Wang[1,4*]

[1]Key Laboratory of Animal Genetics, Breeding and Reproduction of Shaanxi Province, College of Animal Science and Technology, Northwest A&F University, Yangling 712100, China.

[2]Department of Animal and Poultry Production, Faculty of Environmental Agricultural Sciences, Arish University, El-Arish 45511, Egypt.

[3]College of Veterinary Medicine, Northwest A&F University, Yangling 712100, China.

[4]International Joint Agriculture Research Center for Animal Bio-breeding, Ministry of Agriculture and Rural Affairs, Yangling 712100, China.

[*]Corresponding authors: Yulin Chen chenyulin@nwafu.edu.cn; Xiaolong Wang xiaolongwang@nwafu.edu.cn.

Supplementary tables

Supplementary table S1. Additional genomic investigations on sheep tails.

Article titles	Main highlights	References
**Genome-wide detection of CNVs in Chinese indigenous sheep with different types of tails using ovine high-density 600K SNP arrays**	A genome-wide copy number variation (CNV) study of three different sheep breeds (Chinese Large-tailed Han, fat-rumped Altay, and thin-tailed Tibetan) was reported. The study determined 371, 301, and 66 CNV regions with lengths of 71.35, 51.65, and 10.56 megabases (Mb) on 26 pairs of autosomal chromosomes in the investigated breeds, respectively. Additionally, a number of potential genes associated with fat synthesis, metabolism, and deposition were identified in the CNV regions using Gene Ontology (GO) enrichment analysis. In Large-tailed Han sheep, seven genes were identified (*PPARA*, *RXRA*, *KLF11*, *ADD1*, *FASN*, *PPP1CA*, and *PDGFA*), whereas, in Altay sheep, five genes were identified (*PEX6*, *RXRA*, *FASN*, *PPP1CA*, and *PDGFA*), and one gene was identified in Tibetan sheep (*RXRA*). *RXRA* was found in all three breeds, suggesting a basic role in fat metabolism, whereas the other eight genes were found in fat-tailed breeds, suggesting a potential role in tail fat deposition.	[218]
**Genome-wide association studies revealed candidate genes for tail fat deposition and body size in the Hulun Buir sheep**	A genomic analysis of Chinese Hulunbuir sheep (a breed that has two lines; small fat-tailed and big fat-tailed) highlighted *RBM11*, *SMURF2*, *TEN1*, *FBF1*, *TECPR2*, and *ANKRD9* to potentially be associated with fat tail weight; *SETD7*, *DTNBP1*, *INTS9*, and *SKOR1* to be associated with the ratio of carcass weight to tail fat weight; and *FT2D2* and *EPSTI1* to be associated with the tail phenotype.	[219]
**Genomic scan for selection signature reveals fat deposition in Chinese indigenous sheep with extreme tail types**	In three Chinese sheep breeds (Large-tailed Han, Altay, and Tibetan), *WDR92*, *TBX12*, *WARS2*, *BMP2*, *VEGFA*, *PDGFD*, *HOXA10*, *ALX4*, and *ETAA1* were proposed to have an association with the sheep tail formation and *JAZF1* to have an association with the lipid metabolism regulation.	[220]
**Genome wide association study for the identification of genes associated with tail fat deposition in**	A genome-wide analysis study using 40 Large-tailed Han, 40 Altay, and 40 Tibetan sheep suggested *SPAG17*, *TBX15*, *VRTN*, *NPC2*, *BMP2*, and *PDGFD* as candidate genes for the tail phenotype.	[221]

**Chinese sheep breeds**		
**Genome-wide association study and inbreeding depression on body size traits in Qira black sheep (*Ovis aries*)**	In the Chinese Qira black sheep, the *PARK2* gene was proposed for the tail length trait.	[222]
***GLIS1*, a potential candidate gene affect fat deposition in sheep tail**	In a genomic comparison between two groups of sheep breeds (fat-tailed; Mongolian and Small-tailed Han) and (thin-tailed; DairyMeade and East Friesian), previously reported genes, such as *PDGFD* and *LOC101117953*, were highlighted to be located within significantly divergent regions. Additionally, a new candidate gene, *GLIS1*, at OAR1 was proposed to be associated with differences in the sheep tail phenotype.	[223]
**Whole-genome selective scans detect genes associated with important phenotypic traits in sheep (*Ovis aries*)**	Using 253 samples from 13 populations and by performing F_{ST} and XP-CLR, potential novel genes associated with tail fat deposition were revealed, including *CERS6*, *BTG1*, *RYR3*, *SLC6A4*, *NNAT*, and *OGT*.	[159]
**Genome-wide identification of copy number variation and association with fat deposition in thin and fat-tailed sheep breeds**	Three Iranian sheep breeds (*n*=192; 96 fat-tailed Baluchi, 47 fat-tailed Lori-Bakhtiari, and 47 thin-tailed Zel), in addition to 70 samples of Valle del Belice, were used for the identification of CNVs linked with fat deposition. A total of 573 and 242 CNVs were detected in fat- and thin-tailed breeds, respectively. Additionally, 328 and 187 CNV regions were within or overlapping with 790 known genes. A significant copy gained region on OAR6 harboring *HGFAC* and *LRPAP1* genes that are involved in fat metabolism was also detected.	[224]
**Hitchhiking mapping of candidate regions associated with fat deposition in Iranian thin and fat tail sheep breeds suggests new insights into molecular aspects of fat tail selection**	A gene set for the sheep tail phenotype was also suggested when the Iranian fat-tailed Lori-Bakhtiari and thin-tailed Zel were further investigated, including *TCF7*, *PPP2CA*, *PTGDR*, *NID2*, *AR*, *EBP*, *CACNA1F*, *HSD15B*, *SLC35A2*, *BMP15*, *WDR13*, and *RBM3*.	[225]

**Genome-wide DNA methylation patterns of muscle and tail-fat in DairyMeade sheep and Mongolian sheep**	Additionally, a genome-wide DNA methylation analysis was performed in muscle and tail fat tissues isolated from thin-tailed DairyMeade sheep and fat-tailed Mongolian sheep. A number of differentiated methylation regions were detected, in which *CAMK2D* showed significant DNA methylation level differences between the two breeds in both muscle and tail fat tissues. It was also speculated that *CAMK2D* potentially plays a role in fat metabolism and muscle development.	[226]

Supplementary table S2. Sheep tails from a transcriptomic perspective: mRNA profiles.

Article titles	Main highlights	References
**Transcriptome profile analysis of adipose tissues from fat and short-tailed sheep**	Wang et al. 2014 first performed a transcriptome profile analysis of sheep adipose tissues to understand the mechanism underlying sheep tail fat accumulation. The study revealed 646 differently expressed genes (DEGs; 280 upregulated " ↑ " and 366 downregulated " ↓ ") between the fat-rumped Kazakh and thin-tailed Tibetan sheep breeds. *NELL1* and *FMO3* were identified as potential genes relevant to fat metabolism in adipose tissues and other potential pathways with a contribution to fat deposition were also determined.	[227]
**Genome-wide mRNA-seq profiling reveals predominant down-regulation of lipid metabolic processes in adipose tissues of Small Tail Han than Dorset sheep**	Miao et al. 2015 identified 602 DEGs (266 ↑ and 336 ↓) using the transcriptomic data of the Small-tailed Han and Dorset sheep breeds. Notably, among the significantly enriched biological processes, the 'triglyceride biosynthetic process' was the most significant and relevant to the metabolism of adipose tissues. The DEGs within this process (11 out of 12) were downregulated in Han sheep compared to Dorset sheep. The study proposed that the predominant downregulation of lipid metabolic processes in adipose tissues of Small-tailed Han than Dorset sheep could explain, at least in part, the distinguished fat deposition between the two breeds.	[228]
**Comparative transcriptome analysis reveals potentially novel roles of Homeobox genes in adipose deposition in fat-tailed sheep**	Kang et al. 2017 profiled the transcriptomes of subcutaneous, visceral, and tail adipose tissues from Chinese Tan sheep. 1,058 DEGs were identified between the three adipose types, including 218, 324, and 795 in subcutaneous/visceral, subcutaneous/tail, and visceral/tail adipose tissues, respectively. The study also revealed potentially novel roles of Homeobox (HOX) family genes in the ovine adipose deposition, including *HOXC11*, *HOXC12*, and *HOXC13*, that showed high expression in the tail fat tissues. In addition to other HOX-related and tail adipose-expressed genes, such as (*HOTAIR_2*, *HOTAIR_3*, and *SP9*), that were also highly expressed in tail adipose tissues.	[229]
**Transcriptome analysis of adipose tissues from two fat-tailed sheep breeds reveals key genes involved in fat deposition**	Li et al. 2018 analyzed the transcriptomic profiles of perirenal, subcutaneous, and tail adipose tissues obtained from Large-tailed Guangling and Small-tailed Han sheep. The study identified 2,091, 4,233, and 4,131 DEGs between the perirenal, subcutaneous, and tail fat tissues of the two breeds, respectively. Four genes (*FABP4*, *ADIPOQ*, *FABP5*, and *CD36*) were transcribed at very high levels. Additionally, another set of candidate genes,	[230]

	including *FHC*, *FHC*-pseudogene, and *ZC3H10*, were also expressed at a higher level and potentially have an association with fat deposition. Furthermore, nine fat deposition-related genes (*LOC101102230*, *PLTP*, *C1QTNF7*, *OLR1*, *SCD*, *UCP1*, *ANGPTL4*, *FASD2*, and *SLC27A6*) and five extracellular matrix–receptor (ECM)-related genes (*LAMB3*, *RELN*, *TNXB*, *ITGA8*, and *LAMB4*) were suggested to potentially be responsible for the variation in the fat deposition level.	
**Genetic variants in fat- and short-tailed sheep from high-throughput RNA-sequencing data**	Ma et al. 2018 analyzed the genetic variants of high-throughput RNA sequencing data obtained from *longissimus dorsi* muscle, perinephric, and tail adipose tissues of Lanzhou fat-tailed sheep in comparison with Small-tailed Han and Tibetan sheep breeds. About 8.2% of the identified SNPs and small insertions and deletions (indels) were located within QTL regions with potential functional roles related to tail fat traits, including 'tail fat deposition', 'fat density', 'subcutaneous fat area', 'subcutaneous fat thickness', 'subcutaneous fat weight', and 'total fat area'. The study also indicated some of the unique Lanzhou fat-tailed sheep variants (44 SNPs) to be shared with other previously investigated fat-tailed sheep breeds [4,12,20,21]. Additionally, a 1.33 Mb genomic region on OAR3 (39.58–40.91 Mb; Oar_v3.1) containing 33 of the SNPs and embedding three genes (*CREB1*, *WDR92*, and *ETAA1*) were suggested to be associated with the tail fat development.	[231]
**Deep transcriptome analysis using RNA-Seq suggests novel insights into molecular aspects of fat-tail metabolism in sheep**	In another sheep tail transcriptomic study performed in Iranian fat-tailed Lori-Bakhtiari and thin-tailed Zel, 264 DEGs (80 ↑ and 184 ↓) were identified. Of these DEGs, 13 and 34 genes, have previously been reported in similar comparative studies as important DEGs for fat deposition in both sheep and cattle, respectively. Additionally, a set of potential genes, including *ZFP36*, *JUNB*, *RARRES2*, *ADIRF*, *AFT3*, *ARID5A*, *MYBL2*, *GADD45B*, *SCD*, *FASN*, *ACACA*, *CPT2*, *ELOVL5*, *ELOVL6*, *ACSL1*, *EHHADH*, *HSD17B12*, *ACLY*, *COL1A1*, *COL1A2*, *ITGA11*, *TNC*, *COL6A3*, *ESR1*, and *MEF2A*, was suggested to play direct or indirect roles in fatty acid metabolism. The differential expression of such related genes may contribute to the promotion of fat deposition in fat-tailed sheep breeds.	[232]
**A transcriptomic study of the tail fat deposition in two types of Hulun Buir sheep**	The Hulunbuir sheep lines with distinct two tail morphologies and similar genetic backgrounds were previously used to genomically investigate the tail length [14] and size [219]. Fan et al. 2019 used this breed with different tail sizes (large and small) and sexes (male and	[233]

**according to tail size and sex**	female) to investigate the differences in gene expression profiles that affect fat metabolism in tail adipose tissues. A total of 373 DEGs between large-tailed (LTHS) and small-tailed Hulunbuir sheep (STHS) regardless of sex were identified. Among the identified DEGs, 11 genes were associated with fat metabolism, including *ACSL1*, *PLIN1*, *ELOVL5*, *ELOVL6*, *ACACA*, *PTRF*, *PDK4*, *ACLY*, *ACADL*, *AGTR1*, and *EGR1*. Additionally, 775 and 578 DEGs were identified in other comparisons between ♂ LTHS *vs.* ♂ STHS and ♀ LTHS *vs.* ♀ STHS, respectively. Furthermore, 47 and 109 overlapping genes were revealed between ♂ LTHS *vs.* ♀ LTHS and ♂ STHS *vs.* ♀ STHS, respectively. This indicates the potential influence of sex differences on fat metabolism levels.	
**Transcriptomic analyses revealed common tailed and perirenal adipose differentially expressed genes in four Chinese indigenous sheep breeds**	Yuan et al. 2019 further performed a differential expression analysis using RNA sequencing data of the *longissimus dorsi* muscle, perirenal, and tail adipose tissues of the Short-tailed Hu sheep. 1,336, 427, and 665 DEGs were identified in three types of tissues, respectively. Of these DEGs, 336 and 600 genes were uniquely expressed in perirenal and tail adipose tissues, respectively. By integrating the results of previously published RNA sequencing of Small-tailed Han, Large-tailed Guangling, and Tan sheep breeds [229,230], nine and 13 common candidate genes were revealed for the perirenal and tail adipose-specific deposition, respectively. Of these genes, *WT1*, *TCF21*, *PPFIBP1*, and *WNT10B* were highly expressed in perirenal adipose tissues, while *PKD2L1* and *MST1* may have potential roles in maintaining unsaturated fatty acid content in tail adipose tissues. The study also validated the correlation of five previously reported genes with fat deposition, including *CITED1*, *HOTAIR_2*, *HOTAIR_3*, *HOXC12*, and *HOXC13*. Additionally, in the examined sheep breeds, *PPP1CA* and *PEX6* were commonly highly expressed in tail adipose tissues among 42 candidate genes identified in previous genomic investigations.	[234]
**RNA-Seq based genetic variant discovery provides new insights into controlling fat deposition in the tail of sheep**	Bakhtiarizadeh et al. 2020 reported another study using RNA sequencing generated genetic variants. 112,344 SNPs were called from RNA sequencing data of Lori-Bakhtiari and Zel. 2,774 and 10,470 were breed-specific SNPs in the two breeds, respectively. Of the breed-specific SNPs, 724 and 2,905 were located within QTL regions. A number of these QTLs were related to 'total fat area' and 'tail fat deposition'. Furthermore, a set of potential genes, such as *DGAT2*, *ACSL1*, *ACACA*, *ADIPOQ*, *ACLY*, *FASN*,	[235]

	CPT2, *SCD*, *ADCY6*, *PER3*, *CSF1R*, *SLC22A4*, *GFPT1*, *CDS2*, *BMP6*, *ACSS2*, *ELOVL6*, *HOXA10*, and *FABP4*, were proposed to be strongly linked to fat deposition.	
**Molecular mechanisms of fat deposition: *IL-6* is a hub gene in fat lipolysis, comparing thin-tailed with fat-tailed sheep breeds**	Farhadi et al. 2021 performed a transcriptomic comparison in tail adipose tissues between Iranian fat-tailed Ghezel and thin-tailed Zel. 332 DEGs (78 ↑ and 254 ↓) were determined. DEGs that are related to fat metabolism, such as *IL-6*, *NR4A1*, *SOCS3*, *ATF3*, *CREB3L1*, *HIF-1α*, *HMGCS1*, *JUNB*, *LIPG*, *NR4A3*, *FOSL1*, *VPS35*, *VPS26A*, *LTF*, *LBP*, and *MBOAT2*, were highlighted to contribute to the genetic and morphologic diversity of the investigated sheep breeds. DEGs such as *IL-6*, *LIPG*, and *SAA1* were associated with fat lipolysis, whereas *LTF*, *LBP*, *MOGAT1*, and *MBOAT2* were associated with fat deposition. Additionally, the functional enrichment analysis showed pathways associated with fat deposition, including 'fatty acid metabolism', 'fatty acid biosynthesis', and 'HIF-1 signaling pathway'. The study also highlighted the upregulated DEG, *IL-6*, in the thin-tailed Zel to play a potential role in the lipolysis of tail fat. The expression pattern of the *IL-6* gene indicated in this study is inconsistent with that recently reported [236].	[237]
**Identification of key genes in sheep fat tail evolution based on RNA-seq**	Wang et al. 2021 performed a transcriptomic analysis between small-tailed F2 crossed sheep (wild Argali × Chinese fat-rumped Bashby) and typical fat-rumped Bashby sheep, and 873 DEGs were revealed. The study also validated the expression profiles of seven genes (*SCD*, *ESR1*, *EMR1*, *PHYH*, *STAT3*, *GPAM*, and *ALDH1A1*) out of 873 DEGs that have a potential association with fat metabolism using qPCR. The expressional levels of *SCD*, *PHYH*, and *CPAM* were lower in the tail fat tissues of F2 crossed sheep, while the expressional levels of *ESR1* and *EMR1* were higher in the fat tail tissues of Bashby sheep. This suggests that these genes may directly or indirectly influence the fat deposition level in sheep tails.	[238]
**Comparative transcriptome analysis of key genes and pathways activated in response to fat deposition in two sheep breeds with distinct tail phenotype**	Zhang et al. 2021 performed a transcriptomic investigation of sheep tails using the Chinese fat-rumped Altay and thin-tailed Xinjiang Fine Wool that were raised under the same environmental conditions. 21,527 genes were identified and among them, 3,965 DEGs (707 ↑ and 3,258 ↓) displayed significant expression variations in tail adipose tissues. GO analysis revealed that 198 DEGs (72 ↑ and 126 ↓) were related to fat metabolism. Among the 198 DEGs, 22 genes were significantly up or downregulated in the tail adipose tissues of Altay sheep, suggesting potential roles of these genes in the tail fat deposition. Additionally, the	[239]

	expressional levels of *ABCA1*, *PLIN1*, *SORBS1*, *ANGPTL4*, *LPIN1*, *ELOVL5*, *ACACA*, *FASN*, *CIDEC*, *FABP3*, and *SLC27A2* were markedly higher in Altay than in Xinjiang Fine Wool. Whereas, the expressional levels of *CYP4A11*, *FADS2*, *PTPLB*, *ACAA1*, *PPCK1*, *PMP2*, *HSL*, *CPT1A*, *C1QTNF1*, *ACADL*, and *C1QTNF9* were significantly higher in Xinjiang Fine Wool than in Altay. Furthermore, the SNP distributions in the coding regions of the 22 candidate genes were investigated, and seven potential SNPs were examined in populations of three sheep breeds (Altay, Xinjiang Fine Wool, and short fat-tailed Hu). The g.18167532T/C (Oar_v3.1) mutation in *ABCA1* and the g.57036072G/T mutation in *SLC27A2* showed significantly different distributions and were suggested to be associated with the sheep tail phenotype.	
**Transcriptome profiling of developing ovine fat tail tissue reveals an important role for *MTFP1* in regulation of adipogenesis**	Han et al. 2022 using the transcriptomic data of sheep tail tissues at different developmental stages, proposed a functional role for *MTFP1* in sheep tail formation. The *MTFP1* expression was most significantly differentiated between the 70-day old embryonic tail fat tissues of fat-tailed Tan sheep and thin-tailed Suffolk sheep. Additionally, the *MTFP1* knockdown resulted in inhibition of cell proliferation and promotion of fat deposition in primary adipose-derived stem cells isolated from 80-day old embryonic fat tail tissues.	[240]
**Meta-analysis of RNA-Seq datasets highlights novel genes/pathways involved in fat deposition in fat-tail of sheep**	Hosseini et al. 2022 performed a RNA-Seq meta-analysis on sheep fat-tail transcriptomes using six datasets. A total of 500 DEGs (221 ↑ and 279 ↓) were detected. Downregulated DEGs, such as collagen subunits IV, V and VI; integrins 1, and 2; *SCD*, *SCD5*, *ELOVL6*, *ACLY*, *SLC27A2*, and *LPIN1*, were predicted to impair lipolysis or fatty acid oxidation and cause fat accumulation in the tail. Additionally, upregulated DEGs, such as *IL6*, *RBP4*, *LEPR*, *PAI-1*, *EPHX1*, *HSD11B1*, and *FMO2*, were predicted to control fat accumulation in the tail through mediating adipogenesis and fatty acid biosynthesis. The expression pattern of the *IL6* gene indicated in this study is inconsistent with that previously reported [237].	[236]
**Transcriptome study digs out *BMP2* involved in adipogenesis in sheep tails**	Jin et al. 2022 conducted a transcriptomic analysis using tail adipose tissues isolated from Chinese Hu sheep and Tibetan sheep. A total of 2,108 DEGs (861 ↑ and 1,247 ↓) were identified. The study highlighted the potential of *BMP2*, *HOXA11*, *PPP1CC*, and *LPIN1* in the regulation of adipogenesis and fat metabolism. Further molecular analyses emphasized the importance of the *BMP2* gene in regulation of sheep tail formation.	[241]

Supplementary table S3. Sheep tails from a transcriptomic perspective: microRNA profiles.

Article titles	Main highlights	References
**Genome-wide analysis of microRNAs identifies the lipid metabolism pathway to be a defining factor in adipose tissue from different sheep**	Miao et al. 2015 first utilized RNA sequencing technology to perform a genome-wide miRNA expression analysis on adipose tissue samples obtained from the backfat of Small-tailed Han and Dorset. 3,132 miRNAs from the adipose tissues of the Small-tailed Han and Dorset were identified, including 2,893 that were defined as potential new miRNAs. Additionally, 54 miRNAs were differentially expressed (DE) between the two sheep breeds. In Small-tailed Han, of these 54 DE miRNAs, 35 were downregulated and 19 were upregulated. The study also identified 12 downregulated target genes (*AACS*, *THRSP*, *LEP*, *ACACA*, *INSIG1*, *ELOVL6*, *HSD17B12*, *MOGAT2*, *CYP11A1*, *HPGD*, *IDI1*, and *GDE1*) that were negatively correlated with 10 upregulated miRNAs in the Small-tail Han and were enriched in lipid metabolic processes. These results suggested less active lipid metabolism in the adipose tissues of Small-tailed Han.	[242]
**Integrating miRNA and mRNA expression profiling uncovers miRNAs underlying fat deposition in sheep**	Zhou et al. 2017 identified the identity and abundance of miRNAs involved in fat deposition in adipose tissues from fat-tailed Kazakhstan sheep and thin-tailed Tibetan sheep. 815 miRNAs were found in abundance in sheep adipose tissues. Of these 815 miRNAs, 539 miRNAs were commonly shared in both breeds, while 179 and 97 miRNAs were uniquely expressed in each breed, respectively. Additionally, 35 miRNAs were considered putative novel miRNAs, of which 18 and 10 miRNAs were specifically expressed in each breed, respectively. At the mRNA level, 5,162 known genes and 459 novel genes in Kazakhstan sheep, as well as 15,330 known genes and 458 novel genes in Tibetan sheep were identified. Compared to Kazakhstan sheep, 1,931 DEGs were identified (766 ↑ and 1,165 ↓). Furthermore, by performing the GO analysis, the study also indicated that the identified miRNAs potentially play key roles in fat deposition through their ability to regulate essential pathways.	[243]
**MicroRNA expression patterns in tail fat of different breeds of sheep**	Pan et al. 2018 detected the expression profile of miRNAs in two breeds of fat-tailed sheep with different levels of tail fat deposition (Large-tailed Guangling and Small-tailed Han). 113 and 131 conserved miRNAs were detected in the two breeds, respectively, and 40 of these detected miRNAs were DE. Additionally, 208 and 215 novel miRNAs were obtained and 150 of these miRNAs were DE. Of the nine selected miRNAs to validate the sequencing accuracy in 10-	[244]

	month-old individuals, the expression of *miR-29a*, *miR-30c*, *miR-155*, *miR-10b*, *miR-206*, and *novel-miR-36* was higher, while the expression of *miR-192*, *novel-miR-102*, and *novel-miR-63* was lower in Small-tailed Han than Large-tailed Guangling. The pathway analysis of this study also showed that many predicted target genes for the identified miRNAs are involved in lipid metabolism pathways.	
**Identification of the genetic basis for the large-tailed phenotypic trait in Han sheep through integrated mRNA and miRNA analysis of tail fat tissue samples**	Yang et al. 2020 identified mRNAs and microRNAs that were DE in tail adipose tissues from Large-tailed Han and Small-tailed Han. In total, 521 DE mRNAs (237 ↑ and 284 ↓) and 14 DE miRNAs (6 ↑ and 8 ↓) were identified. Additionally, 2,409 putative targets of these DE miRNAs were detected, including 65 targets that were DE. Additionally, the study identified *DIRF*, *HSD17B12*, *LPL*, *APOBR*, *INSIGI*, *THRSP*, *ACSL5*, *FAAH*, *ACSS2*, *APOA1*, *ACLY*, and *ACSM3* using the mRNA data and *ACSL4*, *FTO*, *FGF8*, *IGF2*, *GNPDA2*, *LIPG*, *PRKAA2*, *ELOVL7*, *SOAT2*, and *SIRT1* using the miRNA data as potential genes that regulate fat deposition and fatty acid metabolism in tail adipose tissues.	[245]
**Transcriptome reveals key microRNAs involved in fat deposition between different tail sheep breeds**	Fei et al. 2022 detected 155 DE miRNAs (78 ↑ and 77 ↓) in the tail fat tissues between short fat-tailed Hu and short thin-tailed Tibetan sheep. Specifically, *miR-379-5p* was determined to have an association with tail fat deposition. Additionally, a relationship between *miR-379-5p* and *HOXC9* was proposed.	[246]
***Oar-miR-432* regulates fat differentiation and promotes the expression of *BMP2* in ovine preadipocytes**	Jin et al. 2022 used the mRNA and miRNA data of tail adipose tissues from three short fat-tailed Hu and three short thin-tailed Tibetan sheep. 2,108 DEGs (1,247 ↑ and 861 ↓) and 105 DE miRNAs (43 ↑ and 62 ↓) were detected. Of these miRNAs, *miR-432* showed downregulation between Hu and Tibetan sheep. Additionally, a regulatory relationship was proposed between *miR-432* and the *BMP2* gene.	[247]
**Identification of key miRNAs regulating fat metabolism based on RNA-seq from fat-tailed sheep and F2 of wild Argali**	Wang et al. 2022 performed a miRNA analysis between fat-tailed Bashby sheep and small-tailed F2 crossed sheep (Argali × Bashby). A total of 125 DE miRNAs (76 ↑ and 49 ↓) were detected. Three candidate DE miRNAs (*miR-320d*, *miR-151b*, and *miR-6715*) were highlighted. Additionally, *miR-320d* and *miR-151b* were shown to potentially regulate the tail fat deposition level by targeting *SCD* and *ACACA*, respectively.	[248]
**Further investigations**	Furthermore, other miRNAs were investigated in other types of adipose tissues rather than tail adipose tissues in sheep. These include the investigation of diet influence on	Not applicable

	the expression profile of 15 selected miRNAs in sheep subcutaneous and visceral adipose tissues [249], the influence of *miR-27a* in the differential lipid accumulation between sheep intramuscular and subcutaneous adipose tissues [250,251], the influence of *microRNA-148a* [252] and *microRNA-200b* [253] on the regulation of sheep preadipocyte proliferation and differentiation, and the influence of *miR-193a-5p* in the proliferation and differentiation of 3T3-L1 cells [254]. Taken together, mining different regulatory elements (such as miRNAs) within the transcriptomes of tail adipose tissues of phenotypically distinct sheep breeds and the integrative analyses using the throughput of both mRNA and miRNA data may reveal key regulatory factors influencing the configuration of the tail shape and the accompanying level of fat deposition.

Supplementary table S4. Sheep tails from a transcriptomic perspective: lncRNA profiles.

Article titles	Main highlights	References
**Comparative transcriptome profiling of mRNA and lncRNA related to tail adipose tissues of sheep**	Ma et al. 2018 performed a comparative transcriptome profiling of mRNA and lncRNA using Lanzhou fat-tailed, Small-tailed Han, and Tibetan sheep. The analysis revealed 10 DEGs and 37 DE lncRNAs between the Lanzhou fat-tailed and Small-tailed Han, 390 DEGs and 59 DE lncRNAs between the Lanzhou fat-tailed and Tibetan sheep, and 80 DEGs and 16 DE lncRNAs between the Small-tailed Han and Tibetan sheep, respectively. Among the identified DE lncRNAs, *TCONS_00372767*, *TCONS_00171926*, *TCONS_00054953*, and *TCONS_00373007* were suggested to play potential roles in tail fat deposition. Additionally, GO pathway analyses of DEGs and target genes of DE lncRNAs showed fat deposition-related pathways such as enrichment in fatty acid metabolism-related pathways and fatty acid elongation-related pathways.	[255]
**Identification and expression analysis of long noncoding RNAs in fat-tail of sheep breeds**	Bakhtiarizadeh et al. 2019 reported a lncRNA investigation using the fat-tailed Lori-Bakhtiari and thin-tailed Zel sheep breeds. Among all identified lncRNA candidates, 358 and 66 transcripts were classified as novel intergenic and intronic RNAs, corresponding to 302 and 58 genes, respectively. Additionally, seven DE lncRNAs were identified between the two examined breeds. Furthermore, the lncRNA-mRNA interaction analysis revealed three significant modules with genes related to lipid metabolism, insulin, and calcium signaling pathways.	[256]
**Transcriptome analysis of messenger RNA and long noncoding RNA related to different developmental stages of tail adipose tissues of sunite sheep**	He et al. 2021 performed high-throughput RNA sequencing to identify the expression profiles of lncRNAs and mRNAs in the tail adipose tissues of Chinese Sunite sheep at different developmental stages (six, 18, and 30 months). A total of 223 DEGs and 148 DE lncRNAs were identified in the three developmental stages. The target gene prediction analysis revealed that the majority of the identified lncRNAs target ~20-53 mRNAs as their transregulators. Additionally, fat-related target DEGs were obtained and it was suggested that these target DEGs interact with various DE lncRNAs at different growth stages, playing potential roles in tail fat development. According to the GO and pathway analyses, several fat-related DGEs were identified that were mainly expressed in six-month-old individuals and gradually decreased in 18- and 30-month-old individuals. This suggested that the sheep tail may be expressionally active during the early growth stages and this activity gradually decreases with increasing age.	[257]

**Integrative analysis of Iso-Seq and RNA-seq data reveals transcriptome complexity and differentially expressed transcripts in sheep tail fat**	An additional set of DE lncRNAs between backcross [(Dorper × Hu) × Hu sheep] and grading-up [Dorper × (Dorper × Hu sheep], including *ENSOART00020036299*, *ENSOART00020033641*, *ENSOART00020024562*, *ENSOART00020003848*, and *9.53.1*, were proposed to likely regulate the tail fat deposition.	[258]
**Transcriptome profiling of LncRNAs in sheep tail fat deposition**	LncRNA analysis was also performed between fat-tailed Bashby sheep and small-tailed F2 crossed sheep (Argali × Bashby). 728 DE lncRNAs (458 ↑ and 270 ↓) were identified. LncRNAs, such as *MSTRG.36913*, *MSTRG.24995*, *MSTRG.37980*, *MSTRG.38164*, *MSTRG.36912*, *MSTRG.8169*, and *MSTRG.31389*, were suggested to potentially play a role in fat deposition through the regulation of genes, such as *THRSP*, *FASN*, *SCD*, *GPAM*, *NDUFC2*, *WDTC1*, and *PIK3R1*.	[259]

Supplementary table S5. Sheep tails from a proteomic perspective.

Article titles	Main highlights	References
**Comparative proteome analysis reveals lipid metabolism-related protein networks in response to rump fat mobilization**	Wang et al. 2018 performed a proteomic analysis of the rump of the fat-rumped Altay sheep to investigate proteins controlling the rump fat mobilization. Altay sheep is a breed that has the ability to rapidly mobilize fat in the rump to maintain survival in lengthy, harsh, and cold winter conditions. The authors subjected sheep to persistent starvation to mimic the environmental conditions that induce rump fat mobilization. Then, the isobaric tags for relative or absolute quantitation (iTRAQ) approach was used to perform a quantitative comparison of protein abundance between two groups, the persistent starvation group and a free-feeding group. 112 DE proteins (DEPs) were screened. Increased secretion of *LEP* and *APN* (*ADIPOQ*) was noted to activate the key fat mobilization signaling pathways under persistent starvation conditions. It was suggested that the upregulation of *RETN*, *HSP72*, and *CFD* promoted lipolysis, while the downregulation of *CIDEC* inhibited lipid droplet fusion, and the increase in *HSP72* and *APOA1* levels activated the body's stress mechanisms. This study revealed potential mechanisms for the association of tail fat deposition in the adaptability of Altay sheep to extreme and cold environments.	[260]
**Quantitative proteomic analysis identified differentially expressed proteins with tail/rump fat deposition in Chinese thin- and fat-tailed lambs**	Han et al. 2021 used the label-free proteomic approach to quantitatively analyze the protein abundance in tail/rump adipose tissues of Chinese fat-tailed Kazakh, Hu, and Lanzhou *vs.* thin-tailed Alpine Merino and Tibetan. 3,400 proteins were identified in the five sheep breeds, of which 804 were DEPs (638 ↑ and 83 ↓). Among these, DEPs, FABP4, ACSL1, ACACA, ACLY, FASN, and HSD17β4, were highly expressed in the tail adipose tissues of fat-tailed breeds, suggesting potential roles in tail fat deposition. Specifically, the upregulated proteins that are highly expressed in the fat tail, ACSL1, HSD17β4, and particularly FABP4, were suggested to contribute to tail fat deposition by facilitating the proliferation of adipocytes and tail fat accumulation. These DEPs were also mainly associated with metabolism pathways and the peroxisome proliferator-activated receptor (PPAR) signaling pathway. The PPAR signaling pathway genes, including *FABP4*, *FABP5*, *ACSL1*, *ACSL6*, *PLIN1*, *PLIN4*, *SCD*, *LPL*, *ACAA1*, *ADIPOQ*, etc., were previously suggested and, in this study, they were validated to be associated with tail fat formation and could serve as genetic biomarkers for fat tail traits.	[261]

**Isobaric tags for relative and absolute quantification-based proteomics reveals candidate proteins of fat deposition in Chinese indigenous sheep with morphologically different tails**	Furthermore, a proteomic comparison between fat-rumped Altay and thin-tailed Tibetan sheep identified 3,248 proteins (44 ↑ and 40 ↓). Genes of these DEPs, including *APOA2*, *GALK1*, *ADIPOQ*, and *NDUFS4*, were also highlighted to be associated with fat formation and metabolism.	[262]

Supplementary table S6. Gene expression and association studies of sheep tails.

Article titles	Main highlights	References
**Underlying functional genomics of fat deposition in adipose tissue**	By using express sequence tag (EST) analysis, Bakhtiarizadeh et al. 2013 analyzed the gene expression profiles of adipose tissues in humans, pigs, and cattle. Subsequently, the authors selected the more abundant and common genes in the investigated mammalian adipose tissues for further research in two sheep breeds (fat-tailed Lori-Bakhtiari and thin-tailed Zel). These candidate genes include *FABP4*, *FASN*, *SCD*, and *LPL*. Among these genes, *FABP4* showed a higher expression level than other genes. The *FABP4* expression was also significantly higher in the fat tail tissues of Lori-Bakhtiari sheep than in the fat tail and visceral adipose tissues of Zel sheep. These results highlighted the *FABP4* gene as potentially having an important association with fat deposition.	[263]
***PPARγ*, *FAS*, *HSL* mRNA and protein expression during Tan sheep fat-tail development**	Xu et al. 2015 investigated fat-related genes, including *PPARγ*, *FAS*, and *HSL* at mRNA and protein expressional levels in the fat tails of Chinese Tan sheep. The results of the gene expression analysis showed an oscillation throughout different developmental stages (3, 6, 9, 12, 15, and 18 months).	[264]
**Regulation of lipid metabolism in adipose depots of fat-tailed and thin-tailed lambs during negative and positive energy balances**	Bahnamiri et al. 2017 investigated the expression of a group of fat-related genes, including *PPARγ*, *SREBF1*, *LPL*, *FABP4*, *ACACA*, *SCD*, *ALCY*, and *HSL* in the muscle and adipose depots of pure fat-tailed Lori-Bakhtiari and crossbred thin-tailed Lori-Bakhtiari × Romanov during negative and positive energy balances. The results showed the influence of negative and positive energy balances on the expression profiles of these fat-related genes.	[265]
**Expression of genes related to liver fatty acid metabolism in fat-tailed and thin-tailed lambs during negative and positive energy balances**	Additionally, the transcription regulation of lipogenesis and lipolysis during negative and positive energy balances were shown to occur differently in fat- and thin-tailed lambs. Nonetheless, different energy balances also showed an influence on the expression of genes related to liver fatty acid metabolism in fat- and thin-tailed sheep.	[266]
**Role of *OXCT1* in ovine adipose and preadipocyte differentiation**	Zeng et al. 2019 investigated the functional role of *OXCT1* in the tail adipose tissues of Chinese fat-tailed Tan sheep. *OXCT1* showed higher expressional levels in perirenal and tail adipose tissues than in subcutaneous adipose tissue. In addition, the *OXCT1* knockdown in sheep adipocytes	[267]

	promoted lipid accumulation, suggesting a potential role in adipogenesis.	
**mRNA expression of genes related to fat deposition during *in vitro* ovine adipogenesis**	Li et al. 2019 examined the expression of *PPARγ*, *FABP4*, *FABP5*, and *CRABP2* during *in vitro* sheep adipogenesis. The expression of *PPARγ*, *FABP4*, and *FABP5* was increased upon preadipocyte differentiation. Additionally, *CRABP2* expression was highly increased from days 0 to 2 after induced differentiation and was subsequently decreased. The expression of *PPARγ*, *FABP4*, and *FABP5* was similar when compared with human and mice gene expression retrieved from the Gene Expression Omnibus (GEO) repository; however, the *CRABP2* expression was varied. This indicates that the expression of fat deposition-related genes varies among various species.	[268]
**Verification and analysis of sheep tail type-associated *PDGF-D* gene polymorphisms**	Li et al. 2020 used a lentivirus overexpressing *PDGFD* in sheep tail adipose cells. The qPCR results showed that *PDGFD* was expressed at significantly higher levels in the transduced group than in the control group. Additionally, the expression of *PPARγ* and *LPL* as marker genes of adipogenesis was detected. Based on the obtained results, the authors proposed that *PDGFD* overexpression could upregulate the expression of these adipogenesis marker genes. Furthermore, the Oil Red O staining of sheep preadipocytes on day 7 after the induction of differentiation showed that the number of lipid drops in the *PDGFD*-overexpressing group was higher than that in the control group. The authors proposed that the *PDGFD* could promote the formation of lipid drops in adipocytes *in vitro*.	[269]
**Association between *BMP2* functional polymorphisms and sheep tail type**	Additionally, the expression of the two adipogenesis marker genes (*PPARγ* and *LPL*) was also upregulated when the *BMP2* gene was overexpressed, suggesting the participation of *BMP2* in sheep tail formation.	[270]
**Effect of dietary nutrition on tail fat deposition and evaluation of tail-related genes in fat-tailed sheep**	Zeng et al. 2020 showed the influence of nutrition and growth stage on the expression of fat-related genes (*C/EBPα*, *FAS*, *LPL*, and *HSL*) in the tail adipose tissues of Chinese fat-tailed Tan sheep. The study suggested that tail fat deposition can, to some extent, be regulated by manipulating the diet.	[271]
**Regulatory roles of *SREBF1* and *SREBF2* in lipid metabolism and deposition in two Chinese**	Liang et al. 2020 investigated the expression profiles of *SREBF1* and *SREBF2* in the liver and adipose tissues of Large-tailed Guangling and Small-tailed Han at different stages (4, 6, 8, 10, and 12 months). Although the mRNA expression of the investigated genes was breed-specific, gender-specific, and had temporal and spatial expression	[272]

**representative fat-tailed sheep breeds**	differences, the study proposed a potential role of these genes in the regulation of lipid metabolism during growth and development.	
**Effect of the *ACAA1* gene on preadipocyte differentiation in sheep**	In another study, the lipid metabolism regulator, *ACAA1*, was also investigated. It was found that *ACAA1* was significantly expressed in the early stage of ovine adipocyte differentiation. Furthermore, the *ACAA1* inactivation promoted lipid accumulation, triglyceride content, and preadipocyte differentiation through the upregulation of adipogenic marker genes (*PPARγ* and *C/EBPα*). Additionally, the overexpression of this gene degraded adipogenesis, lipid accumulation, and triglyceride content, suggesting its association with preadipocyte differentiation in sheep.	[273]
**Ovine *ELOVL5* and *FASN* genes polymorphisms and their correlations with sheep tail fat deposition**	In a recent investigation, the expression levels of *ELOVL5* and *FASN* were measured in two breeds with varied tail phenotypes. The *ELOVL5* expression was significantly higher in the large-tailed sheep breed, while *FASN* was significantly higher in the small-tailed sheep breed.	[274]
**Expression features of the ovine *FTO* gene and association between *FTO* polymorphism and tail fat deposition related-traits in Hu sheep**	The expression of *FTO* was also examined in different sheep tissues and the expression was evidently higher in tail fat tissues compared with other tissues. The *FTO* expression was evidently higher in 3-month-old lambs compared to newborns and 6-month-old lambs. Additionally, the *FTO* expression was higher in small-tailed sheep compared to large-tailed sheep.	[275]
**Identification of *TRAPPC9* and *BAIAP2* gene polymorphisms and their association with fat deposition-related traits in Hu sheep**	The expression of *TRAPPC9* and *BAIAP2* was investigated in Chinese Hu sheep. It was shown that the expression levels of these two genes were significantly higher in small-tailed Hu sheep than those in big-tailed Hu sheep.	[276]
**Transcriptome study digs out *BMP2* involved in adipogenesis in sheep tails**	The results of western blotting of the tail fat issues of fat-tailed Hu sheep and thin-tailed Tibetan sheep showed the high expression of *BMP2* in Hu sheep. Additionally, *BMP2* overexpression increased the mRNA levels of *PPAR-γ* and *LOX*. It was predicted that *BMP2* potentially induces adipogenesis through *LOX* in preadipocytes. Furthermore,	[241]

	the number of lipid drops in the *BMP2* overexpression group was greater than that in the negative control group. These results emphasized the involvement of *BMP2* in the formation of the sheep fat-tail phenotype.	
**Further gene expression investigations**	Generally, the expression of other fat-related genes was also investigated in different types of adipose tissues and other body tissues in sheep, showing their potential associations with fat deposition and metabolism in different parts of the body or under specific nutritional conditions. These include *LEP* [277]; *LPL* [278]; *ADIPOQ*, *ADIPOR1*, *ADIPOR2*, *LEP*, *VISFATIN*, *INSR*, and *GPR41* [279]; *ANGPTL4* [280]; *LPL*, *ACACA*, *FASN*, *FABP4*, *DGAT1*, *SCD*, *CPT1B*, *PRKAA2*, *LEP*, *SREBP1*, *PPARG*, *PPARA*, and *CEBPB* [281]; *UCP1* [282]; *H-FABP* [283]; *LPIN2* and *LPIN3* [284]; *RUNX1T1* [285]; *ADIPOR1* and *ADIPOR2* [286]; *ACSL1* [287]; *INSR*, *GLUT4*, *FABP4*, *LPL*, *PPARγ*, *AMPKα*, and *mTOR* [288]; and *HOXC10* [289]. All of the previously indicated gene expression studies have shown the exerting efforts to elucidate the functional mechanisms of fat deposition and metabolism. Most of these gene expression studies are preliminary and still require further large-scale investigations. Examining the expressional profiles of candidate genes in adipose tissues of various sheep breeds with different tail phenotypes at different developmental stages is of importance to reveal their association with fat-related traits.	Not applicable
**SNP association investigations**	Additionally, a number of SNP association studies have also been conducted in an attempt to find potential SNPs that can be used as molecular markers for marker-assisted selection of sheep tail traits. These include genes like *DGAT1* [290], *EDG1*, *AKIRIN2* [291], *FTO* [275,292], *PDGFD* [269], *BMP2* [270], *LIPE* [293], *ELOVL5*, *FASN* [274], *HMGA1* [294] *TRAPPC9*, and *BAIAP2* [276]. It is noticeable that some SNP associations and differentiation can only be found within regional populations and are probably not fully sufficient to be used globally. Thus, the research using this approach seems relatively neglected.	Not applicable

Supplementary table S7. Additional genetic investigations on sheep horn phenotype.

Article titles	Main highlights	References
**A single nucleotide polymorphism on chromosome 10 is highly predictive for the polled phenotype in Australian Merino sheep**	Dominik et al. 2012 fine mapped the genomic location of the horn locus in the Australian Merino sheep to identify significant markers that can be utilized to predict the horn phenotype. The study identified an associated SNP (OAR10_29389966_X.1A>G) using linkage disequilibrium analysis. The SNP was not located within a gene, but it was close to *RXFP2* and *EEF1DP3*.	[295]
**Discovery of SNPs in *RXFP2* related to horn types in sheep**	Later, by resequencing *RXFP2* in Chinese Tan sheep, Wang et al. 2014 reported 20 SNPs (one in the 5'-UTR, four in exons, and 15 in introns). Of these SNPs, a synonymous SNP (c.1125A>G) in exon 14 was highlighted as a potential marker SNP for horn presence/absence in the investigated breed.	[296]
**Genomic prediction of the polled and horned phenotypes in Merino sheep**	Duijvesteijn et al. 2018 attempted to optimize selection for polledness using genomic prediction in Merino sheep. The authors proposed that the elimination of horns can be effective by selecting genotypes *GG* of the OAR10_29458450 SNP or *TT* of the OAR10_29546872.1 SNP since all sheep with these genotypes will be without horns.	[297,298]
**iTRAQ-based quantitative proteomic analysis reveals key pathways responsible for scurs in sheep (*Ovis aries*)**	Furthermore, the horn trait was investigated using a proteomic approach. The iTRAQ-based quantitative proteomic analysis was carried out on horn tissues from both scurred, normal two-horned, and four-horned individuals of Chinese Altay sheep. The study revealed 232 proteins that showed significant differential expression, indicating the variety of proteins that contribute to the generation of different patterns of sheep horns.	[299]

Supplementary table S8. Additional genetic studies on sheep coat color phenotype: The *ASIP* gene.

Article titles	Main highlights	References
**The genetic basis of recessive self-colour pattern in a wild sheep population**	Gratten et al. 2009 investigated the association and expression patterns of the previously reported D_5 in exon 2 and a nonsynonymous mutation (g.5172T>A; p.126Cys>Ser) in exon 4 of *ASIP* between wild-type and self-type coat patterns in Soay sheep (six individuals per group). All the self-type group were homozygous for the D_5 and g.5172A mutations (*n*=6), whereas the wild-type group were either homozygous for the non-deleted allele (N_5) and the g.5172T mutation (*n*=4) or were heterozygous at these positions (*n*=2). Analysis of these variants in data from a larger group (*n*=691) suggested a strong linkage between the two polymorphisms and coat color phenotype. The majority of the self-type group was homozygous for the D_5/g.5172A genotype, whereas most of the wild-type group was homozygous for the N_5/g.5172T genotype. A proportion of the examined individuals were carriers of varied and heterozygous genotypes. The proposed model is that the self-type is generated as the result of a homozygous D_5/g.5172A genotype (non-functional *ASIP*), whereas the wild-type is generated as the result of at least one functional *ASIP*, either a homozygous N_5/g.5172T genotype or a heterozygous D_5/g.5172A-N_5/g.5172T genotype [300]. By analyzing the *ASIP* expression of wild-type (*n*=6) and self-type (*n*=6) groups, the *ASIP* mRNA transcripts were shown in all the wild-type group, whereas five of the six individuals from the self-type group showed no evidence for *ASIP* transcription. An individual with the D_5/g.5172A-N_5/g.5172A was the exception. The study also proposed the potential of a third (putative) *cis*-regulatory mutation in the promoter region to affect the examined coat color patterns.	[301]
**Selection and microevolution of coat pattern are cryptic in a wild population of sheep**	Gratten et al. 2012 also studied the influence of the coat color pattern on the lifetime fitness of Soay sheep from an evolutionary aspect. In the investigated population, the frequency of self-type individuals has declined from 1985 to 2008, and this coat color pattern was shown to be associated with reduced lifetime fitness. This study suggested that the *ASIP* genotype is significantly associated with lifetime fitness.	[300]
**Coat colours in the Massese sheep breed are**	Fontanesi et al. 2011 analyzed the *ASIP* gene in 161 Massese sheep with two coat color types (120 black and 41 grey). It has shown that the presence of one duplicated	[302]

**associated with mutations in the agouti signalling protein (*ASIP*) and melanocortin 1 receptor (*MC1R*) genes**	copy allele, including the *ASIP* gene, was significantly associated with the grey coat color (37 out of 41), whereas animals without a duplicated allele were completely black (117 out of 120). A few exceptions were also observed, where three grey sheep did not carry any duplicated copy alleles and four black animals carried a duplicated copy allele. The study proposed the implication of *MC1R* in patterning the coat color of the examined breed.	
**Analysis of polymorphisms in the agouti signalling protein (*ASIP*) and melanocortin 1 receptor (*MC1R*) genes and association with coat colours in two Pramenka sheep types**	Again, Fontanesi et al. 2012 analyzed the *ASIP* gene in two Pramenka sheep populations (Dubian and Privorian) with no fixed coat color and pattern traits. The absence of duplicated *ASIP* copy allele and almost full dark (black/grey) coat color was significantly associated; however, the association was not complete. One Dubian sheep with the same coat color phenotype was heterozygous with a duplicated and a non-duplicated allele. Additionally, spotted black regions were present in all animals carrying at least a duplicated *ASIP* allele. Hence, it is suggested that additional uncharacterized *ASIP* alleles or interactions with other genes may be involved in the generation of these coat color patterns. The study also observed no association between *MC1R* haplotypes and the investigated coat color patterns.	[303]
**Epistatic interaction of the melanocortin 1 receptor and agouti signaling protein genes modulates wool color in the Brazilian Creole sheep**	Hepp et al. 2016 investigated the interaction of coat-color-related genes in Brazilian Creole sheep, a breed with varied coat colors (ranging from black to white with several intermediate patterns; *n*=410; 148 white and 262 colored individuals). The colored individuals showed dominant mutational patterns in *MC1R*. On the other hand, the white individuals were homozygous for the *MC1R* recessive allele (E^+) and carriers for the duplicated copy of *ASIP*. Additionally, carriers of the duplicated copy of *ASIP* showed increased levels of *ASIP* expression in the skin compared to the not detectable expression in the single homozygous copy. It has also shown that the deletions in *ASIP* exon 2 and the c.5172T>A mutation did not show a significant association with the color groups.	[304]
**Mutations in *ASIP* and *MC1R*: dominant black and recessive black alleles segregate in native Swedish sheep populations**	Furthermore, Rochus et al. 2019 investigated the coat color patterns in native Swedish sheep populations, revealing the segregation of dominant black and recessive black alleles. The black coat color in Klövsjö and Roslag sheep breeds was shown to be associated with the recessive black allele (D_5/g.5172A) in *ASIP*, and the dominant black allele of *MC1R* was associated with black coat color in Swedish Finewool. The explanation of the genetic association with coat color in other investigated sheep breeds like Gotland,	[305]

	Gute, Värmland, and Helsinge was more complex. In these breeds, the genotypes of the black-coated individuals could explain their black color, and the color of grey-coated individuals was proposed to be a result of the *ASIP* duplication.

Supplementary table S9. Additional genetic studies on sheep coat color phenotype: The *MC1R* gene.

Article titles	Main highlights	References
**Sequence characterization of the melanocortin 1 receptor (*MC1R*) gene in sheep with different coat colours and identification of the putative e allele at the ovine *Extension* locus**	Fontanesi et al. 2010 identified five novel SNPs (c.-31G>A, c.199C>T, c.429C>T, c.600T>G, and c.735T>C) composing three haplotypes in *MC1R* in nine Italian sheep breeds, and some of these haplotypes were proposed to have a potential influence on the coat color patterning (e.g., haplotypes 2 and 4). Of these novel SNPs, the c.199C>T was shown to be a nonsynonymous mutation (p.67Arg>Cys; haplotype 2; representing the *e* allele at the ovine *Extension* locus and identified only in a proportion of the Valle del Belice sheep breed). Additionally, the E^D allele was identified in a few Massese sheep. Of the few cases of the black-coated Massese sheep that were carriers for an *ASIP* duplicated copy allele (*n*=4), the presence of the E^D allele in two individuals was proposed to be the cause of their black coat color [302].	[306]
**Mutations in *MC1R* gene determine black coat color phenotype in Chinese sheep**	Additionally, other articles reported previously published SNPs and haplotypes of *MC1R* to be associated with the coat color patterns in different sheep populations. Yang et al. 2013 confirmed the presence of five previously published SNPs of *MC1R* in Chinese sheep breeds (white Large-tailed Han, black-fur Minxian, and brown fat-rumped Kazakh). These include the two mutations of the E^D allele (c.218T>A and c.361G>A) and the three synonymous mutations (c.429C>T, c.600T>G, and c.735C>T). The black-coated breed was a carrier of the E^D allele; however, this allele was absent in the investigated white-coated breeds (Large-tailed Han, Small-tailed Han, Gansu Alpine Merino, and Chinese Merino).	[307]
**Allelic variation of melanocortin-1 receptor locus in Saudi indigenous sheep exhibiting different color coats**	Furthermore, these five mutations were investigated in Saudi Arabian sheep breeds (Najdi, Naeimi, and Herri) and a Sudanese sheep breed (Sawaknee). In this study, the E^D allele was associated with either dark coat color, black or brown, in Najdi and Sawaknee sheep, respectively.	[308]
**Genetic analysis of melanocortin 1 receptor gene in endangered Greek sheep breeds**	The two previously reported nonsynonymous mutations (c.218T>A and c.361G>A), one synonymous mutation (c.735C>T), and one new nonsynonymous mutation (c.789T>C; p.263Leu>Pro) of *MC1R* were also detected in Greek sheep breeds. These reports support the proposed association of the two E^D mutations with the dark coat color in sheep.	[309]

**Epistatic interaction of the melanocortin 1 receptor and agouti signaling protein genes modulates wool color in the Brazilian Creole sheep**	In the Brazilian Creole sheep, the dominant E^D allele was also present only in colored individuals; whereas all white individuals were homozygous for the recessive allele E^+ and their white coat color was shown to be associated with the *ASIP* duplication.	[304]
**Mutations in *ASIP* and *MC1R*: dominant black and recessive black alleles segregate in native Swedish sheep populations**	The black coat color of the Swedish Finewool sheep breed was also shown to be associated with mutations in both *ASIP* and *MC1R* (the dominant E^D allele). Furthermore, two novel missense mutations in *MC1R*, c.452G>A in native Swedish sheep breeds (Gotland, Gute, and Varmland) and c.785C>T in Texel, Gute, Helsinge, Klövsjö, and Värmland, and a probable duplication of the *MC1R* gene in the Gotland sheep breed were also indicated.	[305]
**Genomic mapping identifies two genetic variants in the *MC1R* gene for coat colour variation in Chinese Tan sheep**	Two significant SNPs (rs409651063 and rs408511664) in *MC1R* were identified to be associated with coat color. Of these two SNPs, one was a missense mutation (g.14231948G>A; Oar_v3.1; p.105Asp>Asn), whereas the second was a synonymous mutation (g.14228343G>A). Notably, the genotypes of these two mutations in white-coated individuals were homozygous *GG* and *GG*, whereas in black-headed individuals, they were heterozygous *GA* and *GA*, respectively. Furthermore, by analyzing the g.14231948G>A missense mutation at the skin cDNA level in black-headed sheep, skin samples from the white-coated area showed only the *G* allele, and skin samples from the black-coated area showed both the *G* and *A* alleles. Additionally, the qPCR results showed that the *MC1R* expression is significantly higher in black-coated sheep compared to white-coated sheep.	[89]

Supplementary table S10. Additional genetic studies on sheep coat color variation.

Article titles	Main highlights	References
**Using regulatory and epistatic networks to extend the findings of a genome scan: Identifying the gene drivers of pigmentation in merino sheep**	In Australian Merino sheep, the piebald phenotype is characterized by the presence of one or more asymmetric pigmented spots. This pigmentation phenotype has an economic influence on wool production and industry. García-Gámez et al. 2011 analyzed this trait and identified thirteen genes, including *IGFBP7*, *PDGFRA*, and *CD9*, with a potential association. The study also reported a number of DEGs in potential positions containing highly associated SNPs, including *ATRN*, *DOCK7*, *FGFR1OP*, *GLI3*, *SILV*, and *TBX15*.	[310]
**Genome-wide analysis identifies potentially causative genes explaininga the phenotypic variability in Pinzirita sheep**	By performing a GWAS analysis using Italian Pinzirita and Pinzirita Rocche del Castro sheep genotyped with the Ovine SNP50K BeadChip array, a single strongly associated SNP (rs414461460) on OAR14 was determined. This SNP marker was located within the *CDH13* gene, suggesting a potential role for this gene in coat pigmentation.	[311]
**A stop-gain mutation within *MLPH* is responsible for the lilac dilution observed in Jacob sheep**	In Jacob sheep, a lilac dilution of the black coat color was observed and investigated by WGS in dilute and non-dilute individuals. In this study, three genes were investigated (*MLPH*, *MYO5A*, and *RAB27A*), and a nonsynonymous mutation (g.3451931C>A) within *MLPH* was shown to result in a premature stop codon that potentially causes a gene loss of function.	[312]
**Genetic status of lowland-type Racka sheep colour variants**	By using the Ovine SNP50K BeadChip in the Hungarian lowland-type Racka sheep, several candidate genes were highlighted between the examined black and white individuals, including *HTR5A*, *INSIG*, *CDK5*, and the previously reported *MC1R* gene. These studies highlight the potential of new genes, in addition to the previously reported candidates, to likely be associated with coat pigmentation in sheep.	[313]
**Gene expression analysis identifies new candidate genes associated with the development of black skin spots in Corriedale sheep**	The black skin spots in Corriedale sheep were also investigated [314–316]. A microarray and gene expression analysis of black spots (with and without pigmented fibers) and white skin showed no significant expression difference for coat pigmentation-related genes, such as *ASIP*, *MC1R*, and *C-KIT*, between white skin and black spots. Other genes, such as *C-FOS*, *KLF4*, and *UFC1*, were proposed to potentially be involved in the formation of black spots.	[316]
**Skin transcriptome**	A skin transcriptomic study was performed on sheep with white and black coat colors to identify additional genes	[317]

**profiles associated with coat color in sheep**	involved in coat color formation. 2,235 known DEGs (479 ↑ and 1,756 ↓) and 845 novel DEGs (107 ↑ and 738 ↓) were determined. Of the 49 known coat color genes expressed in the skin, 13 genes showed higher expression in the skin of black sheep, and many of these upregulated genes, such as *DCT*, *MATP*, *TYR*, and *TYRP1*, are members of the components of melanosomes.	
**Transcriptome profiling analysis reveals key genes of different coat color in sheep skin**	In a transcriptomic study using Chinese Bashibai, Yemule white, and Tulufan black, a number of DEGs were revealed. These include 183 DEGs (91 ↑ and 92 ↓) in the white and black comparison, 210 DEGs (56 ↑ and 154 ↓) in the white and cyan grey comparison, and 885 DEGs (584 ↑ and 301 ↓) in the white and light brown comparison. Additionally, pathways including 'melanogenesis synthetic' and 'tyrosine metabolism' were revealed and genes, such as *DCT*, *TYR*, *TYRP1*, *PMEL*, *SLC45A2*, and *MLANA*, were highlighted to be associated with sheep coat pigmentation.	[318]
**Comparative transcriptome and histological analyses provide insights into the skin pigmentation in Minxian black fur sheep (*Ovis aries*)**	In a transcriptomic study between Chinese Minxian black fur and white Hu, 133 DEGs (78 ↑ and 55 ↓) were determined and pigmentation-related pathways such as 'tyrosine metabolism' and 'melanogenesis' were highlighted. Additionally, a set of melanin biosynthesis-associated candidate genes, including *TYR*, *TYRP1*, *DCT*, *DDC*, *MC1R*, *COA2*, and *FZD2*, were detected as DEGs in skin samples of Minxian black fur sheep.	[319]
**Molecular characterization of two candidate genes associated with coat color in Tibetan sheep (*Ovis arise*)**	Further coat color-related genes such as *KIT* and *MITF* were investigated in Tibetan white and black sheep. Two mutations were identified, including a previously detected missense mutation (g.2316G/C) in *KIT* and a synonymous mutation (g.1548C/T) in *MITF* (the *C* allele of *MITF* was predominant in white-coated individuals). Additionally, *MITF* expression at both the mRNA and protein levels was shown to be significantly higher in the skin tissues of black-coated individuals than in white-coated individuals.	[320]
**Distribution and expression of *SLC45A2* in the skin of sheep with different coat colors**	In another report, the *SLC45A2* gene was investigated in black, black spots of piebald, white spots of piebald, and white skins. The expression of *SLC45A2* at both the mRNA and protein levels was increasing in order from white, to white spots of piebald, to black spots of piebald, and black skins.	[321]
***TRP-2* mediates coat color pigmentation in sheep skin**	*TRP-2*, as a member of the tyrosinase family and a key enzyme involved in melanin biosynthesis, was also studied in black, black spots of piebald, white spots of piebald, and white skins. Generally, *TRP-2* expression was higher in	[322]

	black and black spots of piebald skins than in white spots of piebald and white skins. Furthermore, in the ovine melanocytes, *TRP-2* overexpression significantly increased *MITF* expression, suggesting a potential role of *TRP-2* in the regulation of *MITF*.	
**Identification of differentially expressed *Gnαs* and *Gnα11* in sheep (*Ovis aries*) skins associated with white and black coat colors; Expression and tissue distribution analysis of Angiotensin II in sheep (*Ovis aries*) skins associated with white and black coat colors**	Nevertheless, the expression of other genes, including *Gnαs*, *Gnα11*, and *AngII*, was significantly higher in the skin of black-coated sheep compared to the skin of white-coated sheep.	[323,324]
**Expression and distribution of bone morphogenetic protein 4 and its antagonist Noggin in the skin of Kazakh sheep (*Ovis aries*) with a white and brown coat color**	The expression of *BMP4* and its antagonist Noggin were shown to be significantly higher in the skin of brown Chinese Kazakh sheep than in white Chinese Kazakh sheep.	[325]
**Production of brown/yellow patches in the *SLC7A11* transgenic sheep via testicular injection of transgene**	In a transgenic experiment using the testicular injection approach, the overexpression of *SLC7A11*, a gene known to be involved in multiple processes, including pheomelanin production, generated brown/yellow patches in the wool of Chinese Merino sheep. The study showed that the overexpression of *SLC7A11* could promote pheomelanin production in the sheep coat, confirming its involvement in coat color formation.	[326]
**The role of *KLF4* in melanogenesis and homeostasis in sheep melanocytes**	*KLF4* was highly expressed in the black skin of sheep compared with white skin. Additionally, *KLF4* overexpression significantly elevated melanin production and pigment-related gene expression.	[327]

Supplementary table S11. Additional studies on the curly fleece phenotype in sheep.

Article titles	Main highlights	References
**Genome-wide association study for wool production traits in a Chinese Merino sheep population**	Using the GWAS approach, fifteen significant SNPs were identified to be associated with crimp trait, of which seven SNPs were located within known or predicted genes, including *PTPN3*, *TCF9*, *GPRC5A*, *DDX47*, *EPHA5*, *TPTE2*, and *NBEA*.	[146]
**Analysis of lncRNAs expression profiles in hair follicle of Hu sheep lambskin**	The expression profiles of mRNAs and lncRNAs of hair follicles in Hu sheep (straight wool *vs.* small waves) were also investigated. 25 (12 ↑ and 13 ↓) and 75 (16 ↑ and 59 ↓) DE mRNAs and lncRNAs were detected, respectively, and *FGF12*, *ATP1B4*, and *TCONS_00279168* were highlighted to be potentially associated with hair follicle development.	[328]
**Transcriptome analysis reveals candidate genes regulating the skin and hair diversity of Xinji Fine-Wool sheep and Tan sheep**	Bai et al., 2021 revealed a set of candidate genes (*LAMA5*, *OVOL1*, *SRF*, *DHCR24*, *NGFR*, *SMO*, *CDSN*, *HOXC13*, and *KDF1*) to be associated with hair follicle development. Furthermore, members of the zf-C2H2 and homeobox transcription factor families were identified to be associated with the production of finer and denser wool. Their expression was mainly upregulated in 12-month-old Xinji fine-wool sheep compared to 12-month-old Tan sheep and 3-month-old Xinji fine-wool sheep.	[329]
**Wool fiber curvature is correlated with abundance of K38 and specific keratin associated proteins**	Additional proteomic findings also showed that crimp mutant fibers (straight fibers) were linked with a reduced abundance of orthocortex-associated proteins from the KAP6, KAP7, and KAP8 families, and an increased abundance of proteins from KAP16 and KAP19. The orthocortex-associated type I keratin, K38, showed lower abundance, and proteins from the paracortex-associated KAP4 and KAP9 families showed higher abundance.	[330]
**A study of the phosphorylation proteomic skin characteristics of Tan sheep during the newborn and er-mao stages**	In Chinese Tan sheep, a phosphoproteomic analysis of the skin of newborn and 35-day-old (traditionally called Er-mao) stages showed 2,806 phosphorylated proteins and 8,184 phosphorylation sites. Additionally, the phosphorylation levels of KAP4.7 and KAP13.1 were varied between the two stages.	[331]
**Integrated hair follicle profiles of microRNAs and mRNAs to reveal the pattern**	In a miRNA-mRNA co-expression analysis between Hu sheep with hair follicles with small waves and straight hair, miRNAs (such as *oar-miR-143*, *oar-miR-200b*, *oar-miR-10a*, *oar-miR-181a*, *oar-miR-10b*, and *oar-miR-125b*) and miRNA-mRNA pairs (such as *miR-125b* targets *CD34*,	[332]

**formation of Hu sheep lambskin**	*miR-181a* targets *FGF12*, *LMO3*, and *miR-200b* targets *ZNF536*) were identified to potentially influence wool curvature.	
**Further potential genes**	Nevertheless, variants and expression patterns of other potential genes were also highlighted to be associated with wool curliness, crimp, and curvature. These include *KRT1.2*, *KAP1.3* [132], *FST* [333,334], *KRT27*, *KRT31*, *KRT35*, *KRT85* [335], *TCHH* [335,336], *KAP1-2* [337], *KAP8-2* [338], *KRT83* [339], *KAP22-1* [340], *KAP26-1* [341], *DICKKOPF-1 (DKK1)* [342], *KAP20-2* [343], *KRTAP8-1* [344], *BMP7* [345], and *EGR1* [346].	Not applicable

Supplementary table S12. Additional studies of the fine *vs.* coarse fleece phenotype in sheep.

Article titles	Main highlights	References
**Transcriptome analysis reveals skin lipid metabolism related to wool diameter in sheep**	High-throughput RNA sequencing of the skin samples of three Chinese Inner Mongolian fine-wool and three coarse-wool sheep was performed. 19,914 mRNA transcripts were expressed and among them, there were genes with known fiber-related associations, including *KRTAP7-1*, *KRT14*, *WNT10B*, *WNT2B*, *β-catenin*, and *FGF5*. 467 DEGs (21 ↑ and 446 ↓) were determined in sheep with smaller fiber diameters. Additionally, GO and Kyoto Encyclopedia of Genes and Genomes (KEGG) analyses revealed pathways related to lipid metabolism, suggesting a potential influencing role related to wool diameter.	[347]
**A comparison of transcriptomic patterns measured in the skin of Chinese fine and coarse wool sheep breeds**	Furthermore, a transcriptomic analysis between fine-wool Super Merino and coarse-wool Small-tailed Han sheep was also performed. 435 genes were highly expressed in the examined two breeds (127 in Super Merino and 308 in Small-tailed Han). In Super Merino, various genes were highly expressed, including lipid metabolism-related genes, keratins, keratin-associated proteins, and wool follicle stem cell markers. On the other hand, in Small-tailed Han, the mammalian epidermal development complex genes, and skin cornification and muscle function-related genes were highly expressed.	[348]
**Discovery of genes and proteins possibly regulating mean wool fibre diameter using cDNA microarray and proteomic approaches**	It was also indicated that, at the anagen phase, the wool follicle in the skin of Chinese fine-wool Aohan shows a distinct expression pattern when compared to that of coarse-wool Small-tailed Han.	[349]
**Comparative investigation of coarse and fine wool sheep skin indicates the early regulators for skin and wool diversity**	Another transcriptomic study using embryonic dorsal skin (~65 days) from fine-wool and coarse-wool sheep revealed 1,994 DEGs (952 ↑ and 1,042 ↓). These DEGs include marker genes highly correlated with skin and wool follicle morphological differences, e.g., the epithelium (six genes), dermal condensate (38 genes), and dermal fibroblast (58 genes). In the investigated developmental stages, the DEGs were enriched in GO terms, including 'epithelial cell migration and differentiation', 'regulation of hair follicle development', and 'ectodermal placode formation'. Additionally, the KEGG analysis showed enrichment in WNT and Hedgehog signaling pathways that are likely	[350]

	responsible for the differences in skin structure. Additionally, during the early morphogenesis of primer wool follicles, potential effectors, including *APCDD1*, *FGF20*, *DKK1*, *IGFBP3*, and *SFRP4*, were proposed to play a role in the regulation of skin compartments, leading to variations in wool diameter in later developmental stages.	
**Influence of feed restriction on the wool proteome: A combined iTRAQ and fiber structural study**	Diet restriction-induced experimental simulation of seasonal weight loss showed a significant reduction in wool fiber diameter in animals on a restricted diet over a 42-day period. Additionally, the proteomic analysis revealed that the experimental diet restriction significantly increased the expression of *KAP13.1* protein and other proteins from the *KAP6* family in the wool of the examined animals. Although the diet restriction could lead to finer wool, it likely negatively influences other desirable wool characteristics. Further understanding of fineness-related genes is required to control wool production without compromising desirable wool characteristics.	[351]
**Comparative proteomic analyses using iTRAQ-labeling provides insights into fiber diversity in sheep and goats**	In a proteomic investigation of various fibers from sheep (fine & coarse) and goats (cashmere & mohair), proteins with different abundances were revealed. This includes keratin or keratin-associated proteins (*KRTAP11-1*, *KRT6A*, and *KRT38*), hair growth-related proteins (*DSC2, DS*G3, *EEF2*, *CALML5*, *TCHH*, and *SELENBP1*), and proteins related to fatty acid synthesis (*FABP4* and *FABP5*). Additionally, it was shown that the abundance of the *FABP4* protein was higher in wool samples than in fine fiber samples of cashmere, whereas the abundance of *KRTAP11-1* protein was higher in fine fiber samples of cashmere than in wool samples. These two proteins were proposed to play a role in the determination of fiber diameter.	[138]
**Further potential genes**	Furthermore, other genes and variants were also highlighted to be associated with wool diameter. These include *PROP1* [352], *FST* [333,334], *KAP6-1* [353,354], *KRTAP6-3* [355], *KRTAP28-1* [356], *IGFBP2*, and *IGFBP4* [357,358].	Not applicable

Supplementary table S13. Potential genes linked to the occurrence of the number of thoracic and lumbar vertebrae in sheep.

Article title	Main highlights	References
**Whole-genome resequencing of Ujumqin sheep to investigate the determinants of the multi-vertebral trait**	A genomic-based investigation was performed on five multi-vertebral (*n*=14) and three non-multi-vertebral (*n*=13) Chinese Ujumqin sheep. Based on the differential analysis, 17 candidate genes (*LOC101108019*, *CORO1A*, *LOC106991848*, *MFSD4B*, *CTU2*, *LOC101114063*, *IL3RA*, *MAOB*, *BSX*, *CAMK1D*, *MLLT6*, *FOXJ1*, *CAPN12*, *ADAMTSL2*, *GGA1*, *GUSB*, and *TNRC18*) were proposed. However, no exact gene(s) or variant(s) were determined to specifically cause the varied phenotype.	[359]
**Effects of vertebral number variations on carcass traits and genotyping of Vertnin candidate gene in Kazakh sheep**	SNP polymorphisms were found within the *VRTN* gene of Chinese Kazakh sheep. Nine SNPs were determined and the rs426367238 SNP (SNP1259) was suggested to be associated with the thoracic vertebral number. For this SNP marker, sheep carriers of the *CC* genotype have more thoracic vertebrae than *CG* and *GG* genotypes.	[172]
**Expression analysis and single-nucleotide polymorphisms of *SYNDIG1L* and *UNC13C* genes associated with thoracic vertebral numbers in sheep (*Ovis aries*)**	Additionally, an SNP (g.82573325C>A) within *SYNDIG1L* was indicated to be associated with the number of thoracic vertebrae in Chinese Small-tailed Han and Sunite sheep.	[360]
**Association analysis of polymorphism in the *NR6A1* gene with the lumbar vertebrae number traits in sheep**	A polymorphism association analysis in 130 Kazakh sheep was performed for the investigation of the number of lumbar vertebrae. The study showed the association of an SNP (rs414302710A>C) in the exon 8 of *NR6A1* with lumbar vertebrae number, suggesting its use in selection programs for increased lumbar vertebrae number.	[361]
**Analysis of the eighth intron polymorphism of *NR6A1* gene in sheep and its correlation with lumbar spine number**	A potential SNP (IVS8-281G>A) in intron 8 of the *NR6A1* gene was also indicated in an investigation that included two groups of Kazakh sheep with six and seven lumbar vertebrae, respectively.	[362]

Supplementary table S14. Credits and courtesies of photos used in the current review article.

**(1) Creative Commons (CC) photos**				
**Photo description (location within the article)**	**Author(s)**	**Link**	**CC license**	**The photo used**
Polled sheep (Figure 4a)	Kirsty Gilmore	https://en.wikipedia.org/wiki/File:Australian_White_Stud.jpg	CC BY-SA 4.0	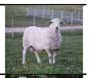
Polled sheep (Figure 4a)	Cgoodwin	https://commons.wikimedia.org/wiki/File:Poll_dorset.jpg	CC BY-SA 3.0	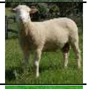
Polled sheep (Figure 4a)	Jackhynes	https://commons.wikimedia.org/wiki/File:Lleyn_sheep.jpg	Public domain	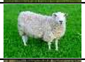
Polled sheep (Figure 4a)	Anton Hornbläser	https://commons.wikimedia.org/wiki/File:Weißköpfiges_Fleischschaf.jpg	CC BY-SA 3.0	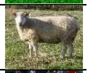
Horned sheep (circular; Figure 4c)	Bwiesem	https://commons.wikimedia.org/wiki/File:Skudde_Bock.jpg	CC BY-SA 3.0	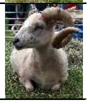
Horned sheep (circular; Figure 4c)	Sheepishly	https://commons.wikimedia.org/wiki/File:Borris_the_ram_shorn.jpg	CC BY 2.0	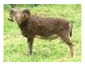
Horned sheep (circular; Figure 4c)	Gailhampshire	https://commons.m.wikimedia.org/wiki/File:Super_Sheep_Portland_on_left_%3F_(24942999407).jpg	CC BY 2.0	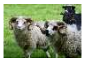
Horned sheep (circular; Figure 4c)	Michael Palmer	https://commons.wikimedia.org/wiki/File:A_black_hebridian_sheep_front_horns.jpg	CC BY-SA 4.0	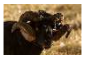
Horned sheep (circular; Figure 4c)	NicePics	https://commons.wikimedia.org/wiki/File:Sheep_with_interesting_horns.jpg	CC BY-SA 2.0	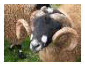
Horned sheep (circular; Figure 4c)	Oskari Löytynoja	https://en.wikipedia.org/wiki/File:Gutebagge.jpg	CC BY 3.0	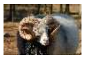
Horned sheep (circular; Figure 4c)	John Haslam	https://commons.wikimedia.org/wiki/File:Horny_Sheep.jpg	CC BY 2.0	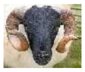
Horned (multi-spiral; Figure 4e)	Francesco Veronesi	https://search.creativecommons.org/photos/01e1c0c0-e82b-4f49-9d1b-af925fbe3603	CC BY-NC-SA 2.0	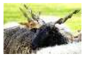
Horned (multi-spiral; Figure 4e)	Francesco Veronesi	https://search.creativecommons.org/photos/c7669d31-c3ad-4ade-86a3-7af5df0303f3	CC BY-NC-SA 2.0	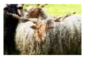
Horned (multi-spiral; Figure 4e)	Pellinger Attila	https://en.m.wikipedia.org/wiki/File:Magyar_racka_juh.jpg	CC BY 3.0	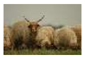
Horned (multi-spiral; Figure 4e)	Bas Bloemsaat	https://search.creativecommons.org/photos/fbb673f1-6b11-4e22-965d-5ccfd0ec7565	CC BY-NC-SA 2.0	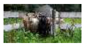
Horned (multi-spiral; Figure 4e)	Tragopan	https://en.wikipedia.org/wiki/File:Zackelschafe_Tiergarten_Bernburg_06-03-2008.jpg	CC BY-SA 3.0	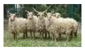
Horned (multi-spiral; Figure 4e)	Tobias Nordhausen	https://search.creativecommons.org/photos/f38f3906-1ba2-433e-a49e-d33827e84ca7	CC BY-NC-SA 2.0	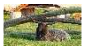
Horned (multi-spiral; Figure 4e)	Maret Hosemann	https://search.creativecommons.org/photos/283d9afd-8b0f-42d2-9480-9c0a4666f4f2	CC BY-NC 2.0	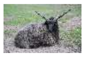

Soay sheep (Figure 5)	Velmc	https://search.creativecommons.org/photos/bd1393e6-7e4c-469b-92dd-e57f7de4f32e	CC BY-NC 2.0	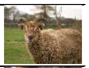
Soay sheep (Figure 5)	Mark Antiquary	https://commons.wikimedia.org/wiki/File:Soay_sheep_at_Sakeham_Farm_1.jpg	CC BY 4.0	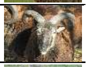
Soay sheep (Figure 5)	Guisnovus	https://search.creativecommons.org/photos/ba550827-6cd7-4dc1-a547-0e5cc002bf35	CC BY-NC 2.0	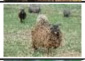
Soay sheep (Figure 5)	UncleBucko	https://search.creativecommons.org/photos/9d81d599-967a-4c91-8026-7e379b25c3a9	CC BY-NC 2.0	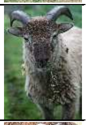
Soay sheep (Figure 5)	Tamsin Cooper	https://search.creativecommons.org/photos/06aed0dc-748f-4be8-9c57-79a8ac333b5a	CC BY-SA 2.0	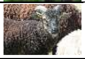
Soay sheep (Figure 5)	Arjecahn	https://commons.wikimedia.org/wiki/File:Soay-sheep-arjecahn.jpg	CC BY 2.0	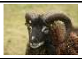
Soay sheep (Figure 5)	Stephen Jones	https://commons.wikimedia.org/wiki/File:Horned_Soay_ram_close-up.jpg	CC BY 2.0	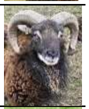
Soay sheep (Figure 5)	Tim Ellis	https://search.creativecommons.org/photos/63d29bb2-9406-4808-bf44-084c0b87a2fa	CC BY-NC 2.0	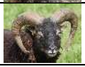
Soay sheep (Figure 5)	Owen Jones	https://search.creativecommons.org/photos/ac97b278-17ae-4a2e-b103-b38205263cc7	CC BY-NC-SA 2.0	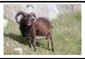
Soay sheep (Figure 5)	Jwh	https://commons.wikimedia.org/wiki/File:Soayschof,_Naturschoul-103.jpg	CC BY-SA 3.0 LU	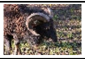
Soay sheep (Figure 5)	Marie and Alistair Knock	https://search.creativecommons.org/photos/ac1be901-78ed-47a5-bef3-46902da30428	CC BY-NC-SA 2.0	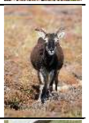
Soay sheep (Figure 5)	Owen Jones	https://search.creativecommons.org/photos/dae834ad-01e0-4c30-917e-a2390ed89228	CC BY-NC-SA 2.0	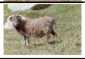
Soay sheep (Figure 5)	Jamain	https://search.creativecommons.org/photos/8d9936bc-47ed-4cfb-9de9-f55927b98d7b	CC BY-SA 3.0	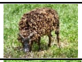
Soay sheep (Figure 5)	Evelyn Simak	https://commons.wikimedia.org/wiki/File:Discarding_the_winter_woollies_-_geograph.org.uk_-_829594.jpg	CC BY-SA 2.0	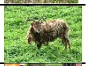
Soay sheep (Figure 5)	Barbara Bresnahan	https://search.creativecommons.org/photos/1578f478-ae4d-409d-94d8-fe831fd01296	CC BY-SA 2.0	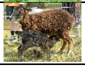
Soay sheep (Figure 5)	Tomek Augustyn	https://search.creativecommons.org/photos/6effaebf-3474-4e4f-a51a-337003200461	CC BY-SA 2.0	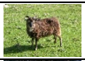
Soay sheep (Figure 5)	Slave2TehTink	https://search.creativecommons.org/photos/7334efec-8408-4493-aae2-b90433233ff4	CC BY-NC 2.0	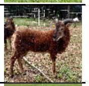
Soay sheep (Figure 5)	Eileen Henderson	https://search.creativecommons.org/photos/f543aeac-acec-4c10-bc6e-f4a9ec8f9c05	CC BY-SA 2.0	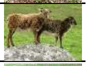
Soay sheep (Figure 5)	Jwh	https://commons.wikimedia.org/wiki/File:Geessen_am_%E2%80%9CM%C3%BChlendelt%E2%80%9D,_Bungeref-101.jpg	CC BY-SA 3.0 LU	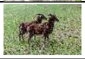
Soay sheep (Figure 5)	Liza Gross	https://journals.plos.org/plosbiology/article?id=10.1371/journal.pbio.0040236	CC BY 4.0	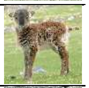
Soay sheep (Figure 5)	Owen Jones	https://search.creativecommons.org/photos/b132c112-b07e-45d9-a50e-f869b6b98f62	CC BY-NC-SA 2.0	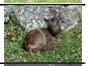
Polycerate (Figure 6)	David Merrett	https://en.wikipedia.org/wiki/File:Jacob_Ram_at_Royal_Show.jpg	CC BY 2.0	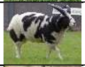
Polycerate (Figure 6)	Malcolm Manners	https://commons.wikimedia.org/wiki/File:Polycerate_sheep_in_Karakol.jpg	CC BY 2.0	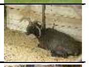
Polycerate (Figure 6)	Crosa	https://en.wikipedia.org/wiki/File:Wildpark_Tambach.jpg	CC BY-SA 2.0	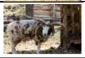
Polycerate (Figure 6)	Jim Champion	https://en.wikipedia.org/wiki/File:The_three-horned_one_again.jpg	CC BY-SA 2.0	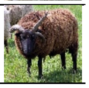

Polycerate (Figure 6)	Rob Hille	https://commons.wikimedia.org/wiki/File:Sheep04.jpg	Public domain	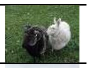
Polycerate (Figure 6)	Mark Dumont	https://commons.wikimedia.org/wiki/File:Jacob_Sheep_at_the_Cinci_Zoo.jpg	CC BY 2.0	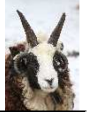
Polycerate (Figure 6)	Jno.skinner	https://en.wikipedia.org/wiki/File:Manx_Loaghtan_sheep_near_Kirkbymoorside_in_Yorkshire.jpg	CC BY-SA 4.0	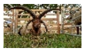
Polycerate (Figure 6)	Sergei S. Scurfield	https://en.wikipedia.org/wiki/File:Jacob_sheep.jpg	CC BY-SA 3.0	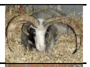
Polycerate (Figure 6)	Chris Bramhall	https://en.wikipedia.org/wiki/File:Manx_loaghtan.jpg	Public domain	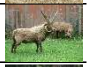
Polycerate (Figure 6)	Tylwyth Eldar	https://commons.wikimedia.org/wiki/File:Zoo_des_3_vall%C3%A9es_-_Mouton_de_Jacob_02.jpg	CC BY-SA 4.0	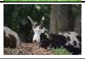
Polycerate (Figure 6)	Frank Vincentz	https://commons.wikimedia.org/wiki/File:Saerbeck_-_Wildfreigehege_N%C3%B6ttler_Berg_-_Jacob_sheep_01_ies.jpg	CC BY-SA 3.0	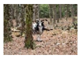
Polycerate (Figure 6)	Geni	https://en.wikipedia.org/wiki/File:Manx_Loaghtan_Butser_Ancient_Farm.JPG	CC BY-SA 4.0	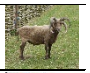
Polycerate (Figure 6)	Adam Jones	https://commons.wikimedia.org/wiki/File:Ram_in_Profile_-_Germiyan_Province_-_Kurdistan_-_Iraq.jpg	CC BY-SA 3.0	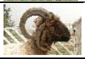
Polycerate (Figure 6)	Acad Ronin	https://en.wikipedia.org/wiki/File:Loaghtan_sheep_-_Jersey.JPG	CC BY-SA 4.0	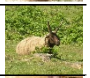
Drop ears (Figure 8b)	Xocolatl	https://en.wikipedia.org/wiki/File:Landwirtschaftliches_Hauptfest_Alpines_Steinschaf_Portrait.jpg	Public domain	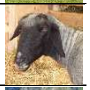
Drop ears (Figure 8b)	Christian Gazzarin	https://en.wikipedia.org/wiki/File:Engadinerschaf_in_den_zwei_Farbschlägen_Braun_und_Schwarz,_Schweiz.jpg	CC BY-SA 4.0	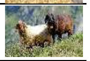
Prick ears (Figure 8c)	Xabier Cid	https://en.wikipedia.org/wiki/File:Border_Leicester_02.jpg	CC BY-SA 2.0	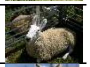
Prick ears (Figure 8c)	Donald Macleod	https://commons.wikimedia.org/wiki/File:Cheviot_ewe_and_lamb.jpg	CC BY 2.0	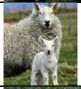
Prick ears (Figure 8c)	Azuschlag	https://en.wikipedia.org/wiki/File:2000_UK_Ewe_of_the_Year_(Clun_Forest_breed,_Court_Llaca_flock).jpg	Public domain	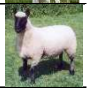
Long-eared (Figure 8d)	Sheharyar Rashid	https://en.wikipedia.org/wiki/File:Kajla_Lamb.jpg	CC BY-SA 4.0	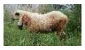
Long-eared (Figure 8d)	Ayazan57	https://commons.wikimedia.org/wiki/File:Posing_Sheep.jpg	CC BY-SA 4.0	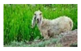
Short-eared (Figure 8e)	CabrioleFarm	https://commons.wikimedia.org/wiki/File:Finnsheep_ewe_being_shorn_by_hand.jpg	CC BY-SA 4.0	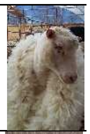
Short-eared (Figure 8e)	Jeffz	https://commons.wikimedia.org/wiki/File:Barbados_Blackbelly.JPG	Public domain	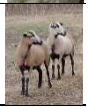
Short-eared (Figure 8e)	Joe Loong	https://commons.wikimedia.org/wiki/File:Mottled_sheep.jpg	CC BY-SA 2.0	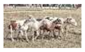
Roman nose (Figure 8f)	Xabier Cid	https://en.wikipedia.org/wiki/File:Border_Leicester_portrait.jpg	CC BY-SA 2.0	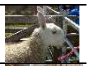
Roman nose (Figure 8f)	BlueLeicester	https://commons.wikimedia.org/wiki/File:Bluefaced_Leicester_Elisabeth_I.jpg	CC BY-SA 4.0	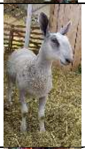
Roman nose (Figure 8f)	Jane Cooper Orkney	https://commons.wikimedia.org/wiki/File:Bluefaced_Leicester.JPG	CC BY-SA 4.0	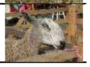

(2) Photo courtesies

Photo description (location within the article)	Author(s)	The photo used
Horned (horizontal; Figure 4d)	Yujing Wu (Tibet Academy of Agricultural and Animal Husbandry Sciences, Lhasa, China)	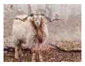
Horned (horizontal; Figure 4d)	Yujing Wu (Tibet Academy of Agricultural and Animal Husbandry Sciences, Lhasa, China)	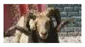
Horned (horizontal; Figure 4d)	Yujing Wu (Tibet Academy of Agricultural and Animal Husbandry Sciences, Lhasa, China)	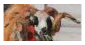
Horned (horizontal; Figure 4d)	Yujing Wu (Tibet Academy of Agricultural and Animal Husbandry Sciences, Lhasa, China)	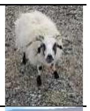
Horned (horizontal; Figure 4d)	Yujing Wu (Tibet Academy of Agricultural and Animal Husbandry Sciences, Lhasa, China)	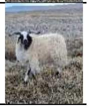
Horned (horizontal; Figure 4d)	Yujing Wu (Tibet Academy of Agricultural and Animal Husbandry Sciences, Lhasa, China)	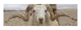
Dewlap (Figure 8a)	Colin Walker (Coolibah Persian Sheep Stud, Australia)	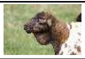
Dewlap (Figure 8a)	Colin Walker (Coolibah Persian Sheep Stud, Australia)	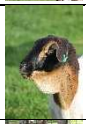
Mane (Figure 8h)	Becky Lannon (breeder) & Lyn Brown (owner)	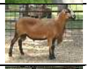
Mane (Figure 8h)	Becky Lannon (breeder) & Lyn Brown (owner)	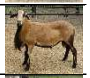
Mane (Figure 8h)	Becky Lannon (Lone Star Farm, Hockley, Texas, USA)	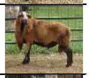
Mane (Figure 8h)	Becky Lannon (Lone Star Farm, Hockley, Texas, USA)	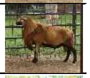
Wattles (Figure 8i)	Venkataramanan Ragothaman (Tamil Nadu Veterinary and Animal Sciences University, Chennai, Tamil Nadu, India)	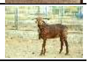
Wattles (Figure 8i)	Venkataramanan Ragothaman (Tamil Nadu Veterinary and Animal Sciences University, Chennai, Tamil Nadu, India)	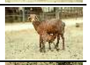
Wattles (Figure 8i)	Venkataramanan Ragothaman (Tamil Nadu Veterinary and Animal Sciences University, Chennai, Tamil Nadu, India)	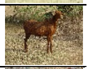
Wattles (Figure 8i)	Venkataramanan Ragothaman (Tamil Nadu Veterinary and Animal Sciences University, Chennai, Tamil Nadu, India)	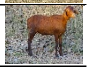
